# Microarray Comparative Genomic Hybridisation Analysis Incorporating Genomic Organisation, and Application to Enterobacterial Plant Pathogens

**DOI:** 10.1371/journal.pcbi.1000473

**Published:** 2009-08-21

**Authors:** Leighton Pritchard, Hui Liu, Clare Booth, Emma Douglas, Patrice François, Jacques Schrenzel, Peter E. Hedley, Paul R. J. Birch, Ian K. Toth

**Affiliations:** 1 Plant Pathology Programme, SCRI, Dundee, Scotland, United Kingdom; 2 Genetics Programme, SCRI, Dundee, Scotland, United Kingdom; 3 Genomic Research Laboratory, Infectious Diseases Service, Geneva University Hospitals and the University of Geneva, Geneva, Switzerland; 4 Division of Plant Science, College of Life Sciences, University of Dundee at SCRI, Dundee, Scotland, United Kingdom; Washington University in Saint Louis, United States of America

## Abstract

Microarray comparative genomic hybridisation (aCGH) provides an estimate of the relative abundance of genomic DNA (gDNA) taken from comparator and reference organisms by hybridisation to a microarray containing probes that represent sequences from the reference organism. The experimental method is used in a number of biological applications, including the detection of human chromosomal aberrations, and in comparative genomic analysis of bacterial strains, but optimisation of the analysis is desirable in each problem domain.

We present a method for analysis of bacterial aCGH data that encodes spatial information from the reference genome in a hidden Markov model. This technique is the first such method to be validated in comparisons of sequenced bacteria that diverge at the strain and at the genus level: *Pectobacterium atrosepticum* SCRI1043 (*Pba*1043) and *Dickeya dadantii* 3937 (*Dda*3937); and *Lactococcus lactis* subsp. *lactis* IL1403 and *L. lactis* subsp. *cremoris* MG1363. In all cases our method is found to outperform common and widely used aCGH analysis methods that do not incorporate spatial information. This analysis is applied to comparisons between commercially important plant pathogenic soft-rotting enterobacteria (SRE) *Pba*1043, *P. atrosepticum* SCRI1039, *P. carotovorum* 193, and *Dda*3937.

Our analysis indicates that it should not be assumed that hybridisation strength is a reliable proxy for sequence identity in aCGH experiments, and robustly extends the applicability of aCGH to bacterial comparisons at the genus level. Our results in the SRE further provide evidence for a dynamic, plastic ‘accessory’ genome, revealing major genomic islands encoding gene products that provide insight into, and may play a direct role in determining, variation amongst the SRE in terms of their environmental survival, host range and aetiology, such as phytotoxin synthesis, multidrug resistance, and nitrogen fixation.

## Author Summary

We describe the first use of a method for the analysis of bacterial microarray comparative genomic hybridisation (aCGH) that includes information about the spatial organisation of genes in the reference bacterium. We demonstrate that using this information improves predictive performance over standard bacterial aCGH methods in discriminating between genes from the reference organism that either do or do not have putative orthologues in the comparator organism. Our approach produces good results on more distantly related bacteria than can successfully be analysed by the standard methods. We apply our analysis to comparisons between four commercially-significant plant pathogenic bacteria, and identify several regions of the genome that are likely to contribute to their ability to cause disease, and to proliferate in the environment, generating hypotheses for future experiments.

## Introduction

Microarray comparative genomic hybridisation (aCGH) provides an estimate of the relative abundance of genomic DNA (gDNA) taken from comparator and reference organisms by hybridisation to a microarray containing probes that represent sequences from the reference organism. This method has been used in a number of biological applications, including the detection of human chromosomal aberrations [1],[2]; comparisons of bacterial human pathogens [3]–[10]; bacterial plant pathogens [11],[12]; industrially-important bacteria [13]; and comparative transcriptomics of *Xenopus laevis* [14].

Numerous algorithms and software packages have been applied to the analysis of this aCGH data in prokaryotes. The majority of these partition reference organism sequences into two mutually exclusive classes: sequences that are ‘present’ and sequences that are ‘absent or divergent’ in the comparator organism [e.g. 5],[12],[15],[16]. Observed hybridisation data are, in each case, assumed to be reliable proxies for these classes.

In this manuscript we describe and apply an improved method for analysis of aCGH data from bacterial genome comparisons. This method incorporates spatial information about CDS location on the reference genome in a hidden Markov model (HMM). This spatial information is expected to capture pertinent biological and evolutionary information, such as operon structure, and regions of lateral gene transfer. Our approach differs from previously proposed, and widely-used, methods applied to bacterial aCGH, such as GACK and MPP, that consider hybridisation intensities of each reference probe as measurements that are independent of their genomic location [15],[16], and is thus more similar to methods such as ArrayLeaRNA [17], which incorporates predicted operon structure into interpretations of microarray expression data, for a restricted set of organisms. We compare the relative performance of our method to commonly used bacterial aCGH analysis algorithms and software.

We demonstrate that several assumptions of common bacterial aCGH analysis methods concerning the relationship between observed hybridisation scores and ratios and the presence or absence of a reference CDS in the comparator organism do not always hold strongly, and that this is particularly the case for more distantly-related organisms. Our data in particular do not support a distinction between ‘present’ and ‘absent or divergent’ classes of sequence, but rather between those sequences in the reference organism that do, and those that do not, have putative orthologues in the comparator genome. We find that the HMM is a better predictor of reference sequences that do not have a putative orthologue in the comparator organism than the other methods tested.

Spatial organisation of sequences on the reference genome has previously been incorporated into methods applied to aCGH analyses of copy number variation in human genomes. This has been represented using HMM [18] and segmentation methods [19]. Simple smoothing methods have also been used to identify breakpoints in this data [20]. However, the problem domain of human copy number aCGH (detecting copy number variation in a known genome sequence) differs from the problem domain of bacterial comparative genomic aCGH (identifying the presence or absence of putative orthologues of known genes in a genome of unknown sequence). To the best of our knowledge, this study describes the first application of a method incorporating such spatial information to aCGH for comparative genomics of unsequenced bacteria, and the first demonstration of the applicability of the technique as a whole across bacterial genera.

*Pectobacterium atrosepticum* (*Pba*), *Pectobacterium carotovorum* (*Pcc*), and *Dickeya* spp. are plant pathogenic soft-rotting enterobacteria (SRE) that share a common ancestor. Despite their many similarities, these commercially significant pathogens differ in their host range, geographical distribution, aetiology and environmental persistence [21]. The molecular origins of these differences are not well understood, but this ecological flexibility is likely indicative of a dynamic, plastic genome with ‘core’ and ‘accessory’ components. There are currently two publicly available annotated genomes for these organisms: *Pba* strain SCRI1043 (*Pba*1043) [22], and *Dickeya dadantii* strain 3937 (*Dda*3937; https://asap.ahabs.wisc.edu/; v6b). The availability of these sequences has rapidly advanced our understanding of these organisms, but broader comparisons are expected to deliver greater insight into the evolution and function of the SRE.

The major common virulence factors of the SRE are plant cell wall degrading enzymes (PCWDE) that degrade the plant cell wall to release nutrients in a so-called ‘brute force’ attack [23]–[25]. Other virulence factors include virulence-associated secretion systems, siderophores, cell-surface polysaccharides and agglutinins [26],[27].

By contrast, bacterial plant pathogens such as *Pseudomonas* spp. are associated with a biotrophic ‘stealth’ interaction with the host. These ‘stealth’ pathogens employ mechanisms such as the type III secretion system (T3SS) to translocate effectors into host cells. The effectors modulate the host plant's biochemical responses, implementing a wide array of strategies to circumvent host immunity [28]–[31]. However, *Pba*1043, *Dda*3937, and other SREs also encode a functioning T3SS and other gene products associated with this ‘stealth’ interaction, indicating a more complex relationship with their hosts than simple ‘brute-force’ necrotrophy [22], [32]–[36]. Key factors with a confirmed role in virulence include type IV and type VI secretion systems, and the phytotoxin coronafacic acid (CFA), which is synthesised by the *cfa* gene cluster [22],[37]. Other factors associated with persistence in, and adaptation to, the wider environment have been identified, such as genes associated with opine uptake, biofilm formation, antibiotic production, and nitrogen fixation [22].

In many bacteria, such genes associated with pathogenicity, and other phenotypically-distinguishing characters, are frequently associated with islands of horizontal gene transfer (HGT). This gene complement is often variable between strains and species, and is sometimes termed the ‘accessory genome’, in order to distinguish it from the ‘core genome’ that provides functionality presumed to be essential to all related organisms [5], [9], [22], [38]–[40]. We expect that observed differences between the gene complements of SRE will reflect differences in their phenotypes, and adaptations to their distinct environments, and that these differences will be preferentially located in islands of genes in their genomes. We use aCGH and apply our analysis method to identify genomic islands in *Pba*1043 that do not have putative orthologues in the unsequenced *Pba* strain SCRI1039 (*Pba*1039) and *Pcc* strain SCRI193 (*Pcc*193), and in the sequenced *Dda*3937. In this study, coding sequences (CDS) from *Pba*1043 that are predicted by aCGH to be absent or divergent in *Pba*1039, *Pcc*193 or *Dda*3937 are of interest because they may potentially contribute to *Pba*1043-specific phenotypes, including host interactions. Pairwise comparisons between *Pba*1043 and these three organisms span a range of evolutionary distances since their most recent common ancestor with *Pba*1043, and represent variation at strain, species and genus levels.

Our results for the SRE support a hypothesis that the genomes of SRE continue to be modified by the acquisition of genomic islands, and the model of an ‘accessory genome’ of niche-specific functionality that is composed, at least in part, of horizontally-acquired genomic islands. We identify major differences in the CDS carried within the accessory genomes of SRE and, while these recapitulate previous observations of major genomic islands made using alternative approaches [22],[38], we also find a number of unexpected differences that provide insight into, and may play a direct role in determining, variation amongst the SRE in terms of their environmental survival, host range and aetiology.

## Materials and Methods

### Genome Sequences and Annotations

Annotated genome sequences were obtained from GenBank for *Pba*1043 (accession: NC_004547), *Lactococcus lactis* subsp. *lactis* Il1403 (accession: NC_002662), and *L. lactis* subsp. *cremoris* MG1363 (accession: NC_009004). Equivalent data for *Dda*3937 was obtained from ASAP (https://asap.ahabs.wisc.edu/; v6b). CDS annotations from these sources were not modified for this study.

### Identification of Putative Orthologues

Putative orthologues of bacterial CDS were identified using reciprocal best hit (RBH) analyses. RBH were identified by using each annotated CDS from the reference genome as the query in a sequence search against the comparator genome, and *vice versa*. A RBH was called when the best match to a query sequence had the query sequence as its own best match in the reciprocal comparison [see also 22],[38]. For protein comparisons, FASTA 3.4t25 was used, and BLASTN 2.2.11 was used for nucleotide comparisons. Reciprocal best hits were interpreted as putative orthologues, and converted to Boolean ‘present’ and ‘absent’ states for model development and training. We would usually employ a threshold for RBH of a minimum of 30% identity over a minimum of 80% of the sequence length for protein comparisons. However, for this analysis we relaxed both criteria completely, and considered the best hit in each direction without such a filter. The division of CDS into ‘present’/‘absent’ classes on the basis of RBH without these thresholds corresponds to a strict classifier for allocating CDS to the ‘absent’ class.

Under the usual circumstances in which we perform these comparative analyses, we wish to exclude weak reciprocal matches from the ‘present’ set in order to avoid inappropriate attribute transfer or assignment. In those cases, we would implement this filter to minimise misallocation of CDS to the ‘present/putative orthologue’ class.

However, in the case of this aCGH analysis, as we note that probes to reference organism sequences that have little or no sequence identity to the comparator may still give very high hybridisation strengths/ratios, we wish preferentially to avoid misallocation of CDS to the ‘absent’ class. Therefore, we aim in effect to give each reference sequence every possible opportunity to be classified as ‘present’ as a putative orthologue in the comparator on the basis of RBH. Any remaining reference CDS that are classified as ‘absent’ - even though no restrictions are made on the basis of sequence identity or match overlap – have no reciprocal similarity by BLAST to any sequence in the comparator.

### Microarray Data: Acquisition

Genomic DNA was extracted from bacterial cell cultures (∼10^10^ cells) using the QIAGEN Genomic-tip 100/G (Qiagen) as recommended and labelling was performed using modified Bioprime DNA Labelling System (Invitrogen). Briefly, 2 µg gDNA in 21 µl was added to 20 µl random primer reaction buffer mix and denatured at boiling for 5 min prior to cooling on ice. To this, 5 µl modified 10× dNTP mix (1.2 mM each of dATP, dGTP, dTTP; 0.6 mM dCTP; 10 mM Tris pH 8.0; 1 mM EDTA), 3 µl of either Cy3 or Cy5 dCTP (1 mM) and 1 µl Klenow enzyme was added and incubated for 16 h at 37°C. Labelled samples for each array were combined (if applicable) and unincorporated dyes removed using Qiaquick PCR Purification Kit (Qiagen) as recommended, eluting twice with 1×50 µl sterile water. Hybridisations and washing were performed as recommended (Agilent Protocol v5.5). Genomic DNA from *Dda*3937 was hybridised to a *Pba*1043-specific microarray (ArrayExpress: E-TABM-600; manufactured by Agilent, AMADID 012663) carrying 5219 unique probes that represent 4450/4472 annotated CDS from the *Pba*1043 genome [37],[41]. Hybridisations were carried out in the presence of *Pba*1043 reference gDNA, *Pcc*193 reference gDNA and in the absence of a reference sample, and all hybridisations were replicated three times. Scanning was performed with an Agilent G2505B scanner using default settings and data extracted using Agilent FE (AGFE) software v9.5.3.

### Microarray Data: Processing

Raw hybridisation data was imported using MatLab (http://www.mathworks.com) from AGFE format output (*Pba*1043 array), and from GEO (*Lactococcus* comparison data, entries: GSM229601, GSM229602, GSM229603, GSM229604) [13]. GEO entries 229602 and 229604 were found to have the labels for channel 1 and 2 inverted, and this was corrected in processing. Raw hybridisation data was corrected for background signal, log-transformed in base 2, then quantile-normalised. Median values were calculated for replicate probes on each array, and then between replicate arrays. Normalised hybridisation scores were associated with a RBH result for each CDS.

### Gaussian Mixture Models

Two-dimensional Gaussian mixture models were fitted in MatLab to the paired hybridisation and RBH data using the gmdistribution.fit function. The optimal number of fitted Gaussians was estimated by the Bayesian Information Criterion (BIC), considering a maximum of ten Gaussians.

### Threshold Models

Threshold models were implemented such that each CDS with a normalised array hybridisation score (or ratio) that fell below the threshold was classified as ‘absent’; those with a normalised hybridisation score above that value were classified as ‘present’. These Boolean states were used for validation of threshold models, and for training of HMMs. Threshold scores were taken at 100 evenly-spaced values between the lowest and highest observed values of hybridisation score (or ratio) for data exploration, and at all observed normalised threshold values (exhaustively to explore all partitions of the data) for rigorous comparisons with alternative models.

### Hidden Markov Model (HMM) Construction

First-order hidden Markov models were trained using MatLab's hmmestimate function, given the Boolean ‘present’/‘absent’ states derived from reciprocal best hit analysis ordered naturally along the reference genome as a ground truth, and Boolean ‘present’/‘absent’ states derived from the threshold models as observed emission states. The derived models represent the presence or absence of a putative orthologue in the comparator sequence as hidden states, in conjunction with the observed hybridisation score being above or below the corresponding normalised hybridisation score threshold, as the emitted states. The resulting models were used to obtain predicted hidden states from hybridisation data using the Viterbi algorithm implemented in MatLab's hmmviterbi function, where input data were again ordered naturally according to probe location on the reference genome.

HMMs used in this study were trained separately on the RBH and hybridisation data for two comparisons: *Pba*1043 and *Dda*3937; and *Lactococcus lactis* subspecies *lactis* IL1403 and *cremoris* MG1363 [13].

### GACK and MPP

The packages GACK and MPP were obtained from their homepages (http://falkow.stanford.edu/whatwedo/software/software.html; http://cbr.jic.ac.uk/dicks/software/mpp/index.html), and used as recommended in their documentation [15],[16]. Array hybridisation data was converted to the appropriate input format in each case using Python scripts. GACK binary and trinary data used in this study was obtained at %EPP cutoffs of 0%, 50%, and 100%, with all other settings at default values. MPP data used in this study was obtained with default settings.

### Model Validation

All predictive models were validated against the ground truth of reciprocal best hit results for *Pba*1043 *vs Dda*3937 or the two *Lactococcus* strains, as appropriate. All model output was obtained as Boolean ‘present’/‘absent’ states, and validation statistics were obtained for consistency tests using MatLab's classperf function (http://www.mathworks.com/access/helpdesk/help/toolbox/bioinfo/ref/classperf.html). Predictions of the absence of a reference CDS in the comparator organism were taken to be ‘positive’ for statistical classification purposes. The optimal HMM and threshold models identified by the validation process were used for subsequent predictions on *Pba*1039 and *Pcc*193 hybridisation data.

### Identification of Regions of Divergent Genome Composition

The software package alien_hunter was downloaded from http://www.sanger.ac.uk/Software/analysis/alien_hunter/ and used to identify regions of divergent genome composition, with recommended settings. This application implements an interpolated variable order motif method derived from the base composition of the chromosome to detect regions of nucleotide bias, and a second-order HMM for change-point detection [42].

### Empirical Statistical Tests

Empirical statistical testing of the association of predicted genomic islands with regions of divergent genome composition as predicted by alien_hunter, and with regions of manually-annotated HGT, was carried out using the following procedure, implemented in a Python script.

The locations of genomic islands predicted by HMM, by alien_hunter, and detailed in the NC_004547 annotation were obtained. These were each considered to represent independent, non-overlapping genomic regions. The location of each of the alien_hunter and NC_004547 regions was shuffled one thousand times, to produce two sets of non-overlapping arrangements of each, representing a random distribution of the predicted islands. A count of the number of HMM-predicted genomic islands that overlapped with each of the shuffled sets was taken, as a measure of the expected number of overlaps that would be obtained if the islands were randomly placed on the genome. The observed count overlap count of the HMM predictions with the alien_hunter and annotated islands was tested for significance using a Z-statistic.

A similar procedure was followed for determining whether individual genes were located preferentially within predicted islands. In this case, the gene locations were taken as static, and genomic island predictions shuffled as non-overlapping regions 1000 times. A Z-statistic was again used to calculate significance of the count of genes observed to be coincident with predicted genomic islands.

## Results

### Reciprocal Best Hit Analysis Indicates That Approximately One Third of All *Pba*1043 CDS Are Absent in *Dda*3937

The genomes of *Pba*1043 and *Dda*3937 have been sequenced and annotated [22] (https://asap.ahabs.wisc.edu/; v6b). CDS were defined to be common to both bacteria if a putative orthologue to a *Pba*1043 CDS could be found in the *Dda*3937 annotation. This was determined for each CDS at the amino acid level by reciprocal best FASTA protein match, and at the nucleotide level by reciprocal best BLASTN match [22]. Each reciprocal best hit (RBH) result was considered to be a putative orthologue (hereafter used interchangeably with ‘orthologue’) and, as a direct and exhaustive sequence comparison, to be the best estimate of the presence or absence of *Pba*1043 CDS in *Dda*3937 available for method validation. The results were used as both reference and training data for aCGH analysis algorithms, in a consistency test approach similar to that used in [4].

Of 4450 *Pba*1043 CDS represented by probes on the microarray, 451 were found to have RBH to *Dda*3937 at both nucleotide and amino acid sequence levels. In addition, 2369/4450 *Pba*1043 CDS made RBH at the amino acid level only, and 7/4450 CDS only at the nucleotide level. For *Pba*1043 1623/4450 CDS therefore have no putative orthologue in *Dda*3937, and it may be considered that approximately one third of the *Pba*1043 genome is not common with *Dda*3937 (Figure S1). Very few *Pba*1043 CDS were found to be orthologous at the nucleotide, but not the protein level (a pattern suggestive of positive selection); however, many were orthologous at the protein, but not at the nucleotide, level (suggestive of neutral drift). The ‘core’ of CDS with both protein and nucleotide-level orthologues was found to comprise only around 10% of the *Pba*1043 genome.

### Array Hybridisation Intensities Have a Complex Relationship with Sequence Identity for *Pba*1043 and *Dda*3937 Orthologues

**Figure 1:**
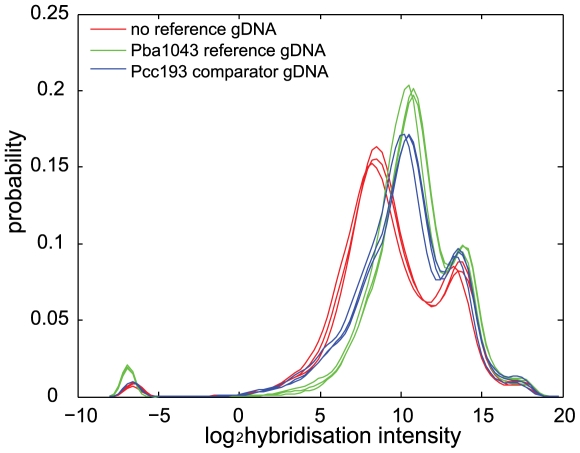
Probability density function curves of log-transformed raw probe hybridisation intensities for the hybridisation of *Dda*3937 gDNA to a *Pba*1043 microarray. Curves are plotted for hybridisation of *Dda*3937 gDNA in the absence of reference gDNA (red), and cohybridised with *Pba*1043 reference gDNA (green), or cohybridised with *Pcc*193 comparator gDNA (blue). Three major populations of probe intensities are seen: strong hybridisation (intensity peak at approximately 14 log units), weak hybridisation (peaks at 8–10 log units), and very weak to no hybridisation (less than −5 log units). Three replicates are indicated for each experiment.

Genomic DNA from *Dda*3937 was hybridised to a *Pba*1043-specific microarray in the presence, independently, of *Pba*1043 reference gDNA and *Pcc*193 reference gDNA, and also in the absence of a reference sample. Three overlapping populations of raw hybridisation strengths were observed in each experiment (Figure 1). This pattern was similar to that observed in similar experiments [12], and comprised: a strongly-binding population of *Pba*1043 probes that bind to *Dda*3937 gDNA with hybridisation strength comparable to their binding to *Pba*1043 gDNA; a weakly-binding population of probes with lower hybridisation strength to *Dda*3937 than to *Pba*1043 gDNA; and a population with either no detectable, or very weak, hybridisation to *Dda*3937 gDNA (Figure 1).

This observation does not support the assumption commonly made in aCGH analysis methods that there are two populations of probes in a typical experiment: ‘present’ and ‘absent or divergent’ [e.g. 3],[8],[11],[15],[16]. Notably, there is no *a priori* indication that any of the three observed populations in Figure 1 comprise ‘present’, ‘absent’ or ‘divergent’ sequences.

**Figure 2:**
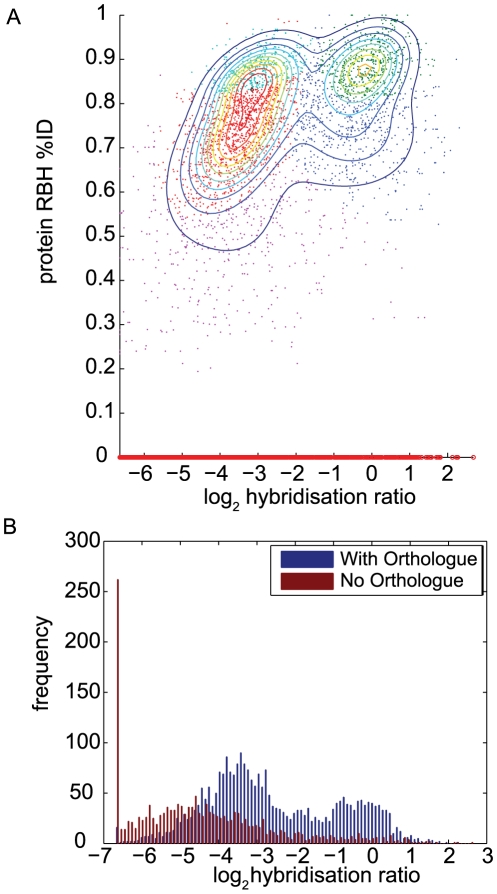
Scatterplot of RBH sequence identity and CDS count against hybridisation ratio. A) Scatterplot of putative orthologue (RBH) protein sequence identity against log-transformed probe hybridisation ratio for *Dda*3937 gDNA cohybridised with *Pba*1043 reference gDNA, for all probes. Sequences with no orthologue are allocated zero sequence identity. Probe population density contours derived from Gaussian mixture modelling are superimposed, and the corresponding Gaussian mixture components are distinguished by coloured points. Three major populations of probes are seen: a strongly hybridising population centred at hybridisation ratio 0 and %ID 0.87; a weakly-hybridising population at hybridisation ratio −3 and %ID 0.82; and *Pba*1043 sequences that have no putative orthologue (red points along the *x*-axis). Strongly- and weakly-hybridising probe sets each cover a range of sequence identities from 30% to 100%, and the probes representing sequences with no orthologues cover a range of hybridisation ratios from −7 to 3. B) Bar plot of the count of CDS by hybridisation ratio, where CDS are divided into two classes: those with a putative orthologue (blue), and those without a putative orthologue (red), as found by RBH analysis. Both classes of CDS span a similar range of observed hybridisation ratios and overlap significantly. The set of CDS with no putative orthologue includes the majority of CDS in the lowest bin of hybridisation ratio.

A linear, or at least monotonic, relationship between the observed hybridisation score and the sequence identity of CDS in the reference and comparator organisms has previously been proposed or observed for aCGH experiments [13],[43],[44]. We did not observe such a relationship. Our data indicated a complex relationship between sequence identity and probe hybridisation affinity (or log ratio), from which three major populations of probes could readily be distinguished (Figure 2). Those probes representing *Pba*1043 sequences that made RBH with greater than 30% amino acid sequence identity in *Dda*3937 were considered here to be putative orthologues and therefore may only be classed as either ‘present’ or ‘divergent’, according to the scheme commonly used in aCGH analyses [e.g. 5],[12],[15],[16]. The ‘absent’ sequence set in that scheme corresponds to *Pba*1043 CDS with no putative orthologue in the comparator organism.

In the *Pba*1043:*Dda*3937 comparison, probes matching orthologous sequences could be resolved into two distinct populations on the basis of hybridisation strength using Gaussian mixture models, but not on the basis of their sequence identities (Figure 2A). In particular, the distribution of putative orthologues was bimodal with respect to hybridisation score or ratio, but was unimodal with respect to sequence identity. Sequence divergence was measured in terms of sequence identity, and it was not possible to distinguish between ‘present’ and ‘divergent’ orthologues using hybridisation data. The commonly-used ‘absent or divergent’ classification is the union of the sets of ‘absent’ and ‘divergent’ sequences; our data does not support this distinction between ‘present’ and ‘absent or divergent’ probe sets.

Those probes corresponding to *Pba*1043 CDS that were not found to have putative orthologues in *Dda*3937 (i.e. that are ‘absent’) were observed to have hybridisation ratios that ranged from no measurable hybridisation to very strong hybridisation, and to take values on the full range of hybridisation ratios spanned by both ‘present’ and ‘divergent’ CDS. The distribution of hybridisation ratios for probes representing putative orthologues overlapped to a great extent that of probes corresponding to CDS with no orthologue (Figure 2B).

Similar results were obtained for nucleotide sequence comparisons, and for raw hybridisation scores (Figure S2). As can be seen from Figure S2, the observed relationship between sequence identity and hybridisation affinity is qualitatively almost identical whether obtained using hybridisation intensity (univariate) data, or hybridisation ratio (bivariate) data. The complexity of this relationship is therefore not due to the use of a log-ratio summary of the hybridisation signal.

Taken together, these results indicated that a distinction might reasonably be drawn between ‘putatively orthologous’ and ‘putatively non-orthologous’ CDS on the basis of aCGH, but not between ‘present’ and ‘absent or divergent’ CDS.

### An Optimal HMM-Based Predictive Model Predicts Which *Pba*1043 CDS Have No Orthologue in *Dda*3937 Better Than an Optimal Threshold Model

Analytical models for aCGH based on a single threshold that partitions CDS into ‘present’ and ‘absent or divergent’ classes have previously been shown to perform acceptably well under some circumstances [e.g. 5], [44]–[46]. However, while the data obtained in this study did not support that particular interpretation of the partitioning of sequences, a threshold approach may still distinguish successfully between reference CDS that do and do not have a putative orthologue in the comparator organism.

The influence of horizontal gene transfer has been in many cases to introduce islands of genes whose collective function distinguishes the recipient organism from its close relatives, as part of the ‘accessory’ genome [5],[9],[22],[38],[39],[47]. One notable influence of HGT on the reference genome is to confer collocation of transferred genes in that genome; such transferred genes may additionally be expected not to have an orthologue in a given comparator genome. In particular, it would be expected that, where a reference genome CDS has been acquired by HGT of a genomic island, it and its neighbours are less likely to have an orthologue in a comparator genome than another CDS randomly selected from the reference genome. Similarly, prokaryotic genes are frequently collected into operons, collocated groups of sequences that often work towards a common function. Loss of function may thus entail loss of a collocated set of genes. We implemented a HMM that exploits this anticipated collocation of sequences on the reference genome, particularly if they have no orthologue in the comparator, in the expectation that taking into account this spatial bias would improve predictive performance in the presence of data noise, and in marginal cases that are difficult to resolve with only a single threshold-based predictor. Such cases might include genes with an unexpected level of redundancy in the comparator organism, such as those with variable copy number due to representation on plasmids [5].

Threshold and HMM models (as defined in Materials and Methods) were constructed for all hybridisation scores and ratios observed in each array experiment, exhaustively enumerating all such models that could be constructed from the data. All possible outcomes of each method were thus obtained, facilitating general claims concerning their performance on this data. In each experiment, a threshold model could be obtained that performed acceptably well when distinguishing between *Pba*1043 CDS that do and do not have putative orthologues in *Dda*3937. However, the optimally performing HMM outperformed the optimally performing threshold model on measures of correct prediction rate and specificity, in consistency tests for all such experiments (Table 1).

**Table 1:** Consistency test validation statistics for *Pba*1043:*Dda*3937 aCGH comparisons.

Prediction method	Data used	Reference gDNA	Correct Positive Rate	Sensitivity	Specificity
Threshold	hybridisation intensity	None	0.6672	0.9592	0.1620
Threshold	hybridisation intensity	*Pba*1043	0.7587	0.8943	0.5239
Threshold	log hybridisation ratio	*Pba*1043	0.7715	0.8975	0.5534
HMM	hybridisation intensity	None	0.6952	0.9585	0.2399
HMM	hybridisation intensity	*Pba*1043	0.7715	0.9078	0.5355
HMM	log hybridisation ratio	*Pba*1043	0.7796	0.9060	0.5607

**Figure 3:**
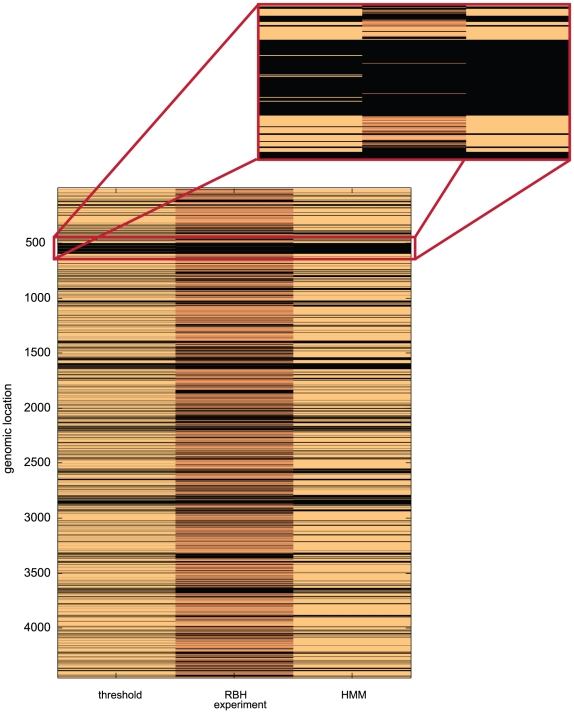
Predictions of putative orthology for aCGH comparisons. Predictions of putative orthology for the *Pba*1043:*Dda*3937 aCGH comparison using log-transformed hybridisation ratios, for both threshold (left) and HMM (right) methods, compared to the locations of known orthologues obtained by *in silico* comparison (centre). CDS are in genomic order from top to bottom of the figure, and black bars indicate CDS from *Pba*1043 with no orthologue in *Dda*3937. For the *in silico* comparison, a brighter copper tone indicates greater sequence identity for that orthologue. The threshold and HMM results are similar, and broadly consistent with each other and the *in silico* analysis. The two prediction methods differ qualitatively in that the HMM method tends to predict larger contiguous islands of CDS with no orthologue than the threshold method, which predicts a greater number of CDS ‘orphans’, as illustrated by the inset that expands the region surrounding HAI2. This region contains the coronafacic acid synthesis *cfa* gene cluster. The RBH comparisons for this island indicate two putative orthologues in *Pba*1043 and *Dda*3937, effectively breaking the island into three smaller islands. The threshold method identifies five larger contiguous putative shared CDS, dividing the island into six smaller regions. The HMM method identifies the island as a single large contiguous region, with no breaks.

It was observed that HMMs and threshold models constructed from experiments involving reference gDNA performed significantly better than those constructed from experiments where no reference gDNA was used. Also, models built using log hybridisation ratios performed better than those derived from single-channel raw hybridisation scores (Table 1). Using log-transformed ratio data, the threshold and HMM predictors predicted that similar total numbers of CDS from *Pba*1043 did not have a putative orthologue in *Dda*3937 (HMM: 1179; threshold: 1191; *in silico* analysis: 1630) but differed in their classification of 372 (approximately 30%) of these CDS. The predictions made by the two approaches differ qualitatively, rather than quantitatively (Figure 3). The HMM predictions appear to form larger contiguous islands of CDS on the genome, while the threshold method predicts a greater number of ‘orphan’ CDS with no orthologue whose immediate neighbours are predicted to have orthologues, and splits several large islands (confirmed as single islands by *in silico* sequence comparison) into several smaller fragments.

Additionally, the behaviour of each model is seen to differ as the hybridisation ratio threshold varies from the minimum to maximum observed value. Both models predict a mixture of CDS with and without orthologues in the comparator at low hybridisation ratios, but at high ratios the threshold model predicts that all *Pba*1043 CDS are without an orthologue in *Dda*3937. At high hybridisation ratio thresholds, the HMM assigns the majority state for the data to all CDS (Figure S3). Also, ‘blocks’ of contiguous sequences with no orthologue in the comparator persist to higher hybridisation ratios, using the HMM approach.

### HMM-Based Predictors Validated on *Pba*1043:*Dda*3937 and *Lactococcus lactis* aCGH Data Perform Better Than GACK or MPP

Two packages for analysis of bacterial aCGH data are GACK (perhaps the most widely-used such application) and MPP, amongst a wide range of proposed alternative aCGH analysis algorithms [10], [15], [16], [48]–[52]. Nearly all of these methods make the assumption that array probes partition into ‘present’ and ‘absent or divergent’ classes, and that these classes are unimodal. It was seen that this assumption is not met in the *Pba*1043:*Dda*3937 comparison but, as for the threshold-based classification, it is likely that these applications are able to segregate CDS from *Pba*1043 that do have orthologues in *Dda*3937 from those that do not.

We applied GACK and MPP to the same log hybridisation ratio data for the *Pba*1043:*Dda*3937 comparison that was most informative for both the HMM and threshold methods above. In GACK it is possible to modify the required stringency of the prediction by varying a parameter representing “estimated probability of presence” (EPP). This may be set at values ranging from 0% - indicating an expectation of statistical ‘certainty’ that CDS predicted to have no orthologue in the comparator organism truly have no such orthologue - to 100% - indicating an expectation of statistical ‘certainty’ that CDS predicted to have an orthologue in *Dda*3937 truly do have such an orthologue. GACK was applied with EPP values of 0%, 50% and 100%, in binary prediction mode. With these settings, GACK predicted that 84 (0% EPP), 344 (50% EPP) or 595 (100% EPP) CDS from *Pba*1043 have no putative orthologue in *Dda*3937 (Figure S4).

MPP with default settings predicted that no *Pba*1043 CDS were without a putative orthologue in *Dda*3937 – the majority state - and thereby achieved a correct prediction rate of 0.65. Although GACK obtained a correct prediction rate of 0.75 at 100% EPP, its sensitivity was very low and, unlike the threshold and HMM methods, neither GACK nor MPP identified a substantial proportion of the 1630 *Pba*1043 CDS that do not have an orthologue in *Dda*3937. Validation statistics for these analyses are shown in Table 2, and indicate that the HMM outperformed both GACK and MPP on the *Pba*1043:*Dda*3937 comparison in terms of sensitivity and total number of correct predictions, although GACK obtained better positive predictive rates at the expense of much reduced sensitivity.

**Table 2:** Statistics for validation of aCGH analytical methods on *Pba*1043:*Dda*3937 and *Lactococcus* comparisons.

Analysis Method	Correct Prediction Rate	Positive Prediction Rate	Sensitivity	Count
*Pba*1043:*Dda*3937				1630
HMM (*Pba*1043:*Dda*3937)	0.7796	0.7752	0.5607	1179
GACK (0% EPP)	0.6512	0.9642	0.0497	84
GACK (50% EPP)	0.7011	0.9360	0.1975	344
GACK (100%EPP)	0.7355	0.8807	0.3214	595
MPP (BPP)	0.6337	0.0000	0.0000	0
MPP (EPP)	0.6337	0.0000	0.0000	0
*Lactococcus lactis*				379
HMM (*Lactococcus*)	0.8404	0.7253	0.1741	91
GACK (0% EPP)	0.8210	0.0000	0.0000	0
GACK (50% EPP)	0.7616	0.2418	0.1557	244
GACK (100%EPP)	0.7616	0.2418	0.1557	244
MPP (BPP)	0.8220	1.0000	0.0053	2
MPP (EPP)	0.8210	0.5000	0.0053	4

It is possible that the less impressive performance of GACK and MPP observed for the *Pba*1043:*Dda*3937 comparison was due to the relatively large evolutionary distance between these organisms, or to the particular array configuration used in these experiments (see Discussion). Most aCGH studies have hitherto focused on variation at the subspecies level, and this is the domain on which GACK and MPP have previously been and, it was assumed, were intended to be, applied [5],[9],[13],[15],[16]. In order to compare the performance of the HMM to GACK and MPP on a comparison of sequenced bacteria with a more recent common ancestor, data for aCGH between *Lactococcus lactis* subspecies *lactis* IL1403 and *cremoris* MG1363 [13], employing an alternative array platform, was obtained from the GEO public repository. The HMM approach again outperformed both GACK and MPP in terms of sensitivity, correct positive rate, and positive predictive rate on this comparison data (Table 2). Although GACK more closely approximated the number of non-orthologous sequences in its predictions, its false positive rate was found to be rather high.

### Comparison of HMM-Based, GACK and MPP Model Performance on *Pba*1043:*Pcc*193 and *Pba*1043:*Pba*1039 Data

*Pcc*193 and *Pba*1039 gDNA was hybridised to the *Pba*1043-specific microarray, in separate experiments, using *Pba*1043 gDNA as the reference in each. The distribution of log hybridisation ratios was found to be approximately unimodal in both cases, reflecting the relatively close evolutionary relationship between these organisms (data not shown).

MPP, with default settings, was unable to fit curves to the hybridisation data from the *Pba*1043:*Pcc*193 experiment, and so its performance was not further assessed. The HMM trained on *Pba*1043:*Dd*a3937 comparison data predicted that 440 *Pba*1043 CDS have no orthologue in *Pcc*193. GACK predicted that between 1187 (EPP: 0%) and 1846 (EPP: 100%) *Pba*1043 CDS have no orthologue in *Pcc*193. As noted earlier, *in silico* sequence comparisons indicated that 1643 *Pba*1043 CDS have no orthologue in *Dda*3937, whose most recent common ancestor with *Pba*1043 is more ancient than that of *Pba*1043 and *Pcc*193. It would therefore be expected that more *Pba*1043 CDS would have orthologues in *Pcc*193, than in *Dda*3937. This implies that the GACK prediction for the *Pcc*193 comparison at 100% EPP is an overprediction. There was also a large discrepancy between the prediction count from HMM and the most conservative GACK prediction at 0% EPP, in that GACK predicted nearly three times as many CDS to be without an orthologue in *Pcc*193 than did the HMM.

The *Pba*1043:*Pba*1039 comparison experiment was a comparison between reference and comparator organisms at the strain level. The HMM built from the *Pba*1043:*Dda*3937 comparison data may be inappropriate for analysis of more closely-related organisms, and so the second HMM, trained separately on the *Lactococcus* comparison data, was also tested. MPP predicted that 299 *Pba*1043 CDS have no orthologue in *Pba*1039, and GACK predicted between 335 (EPP: 0%) and 637 (EPP: 100%) such CDS. The HMM built on the more divergent *Pba*1039:*Dda*3937 comparison predicted 198, and the HMM built on the more recently-diverged *Lactococcus* comparison predicted 255 such CDS. The variation in prediction totals between the HMMs built on the two distinct comparisons is not as great as the variation between the HMM predictions and those made by GACK and MPP, and the predictions made by the HMMs are each in close agreement, implying that the HMM approach is reasonably robust to training set variation, independent of the organism on which it was trained (Figure S5).

While no genome sequences were publicly available at the time of submission to validate these particular predictions, some trends may be inferred from this data. GACK appeared to predict a greater number of CDS to be absent than did the HMM. This behaviour, which potentially results in an increase in sensitivity at the expense of specificity, has previously been reported by other groups [e.g. 5]. Qualitatively, both GACK and MPP predicted a greater proportion of ‘orphan’ CDS, while the HMM favoured prediction of islands of CDS with no orthologue in the comparator (Figure S5). This may be a more biologically appropriate prediction mode.

We observed apparent overprediction, combined with reduced sensitivity and diminished correct positive prediction rates for the GACK and MPP methods, in comparison to the HMM approach. We also found that variation in results between HMMs built on alternative training sets is minor. Thus we proceeded to consider the biological implications of aCGH results obtained for the *Pectobacterium* and *Dickeya* species investigated, using only results obtained using the HMM analysis model built from the *Pba*1043:*Dda*3937 comparison.

### The HMM-Based Predictive Model Predicts Genomic Islands in *Pba*1043 That Correspond to Putative *Pectobacterium atrosepticum*-Specific and *Pectobacterium-*Specific ‘Accessory’ Genomes

**Figure 4:**
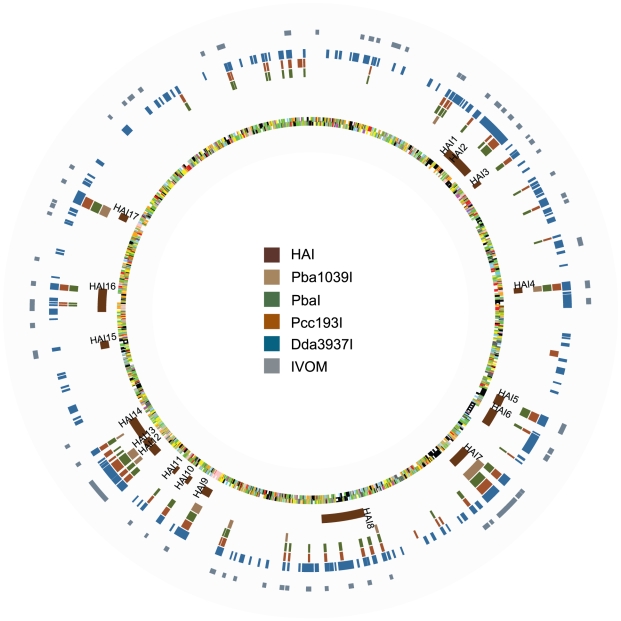
Circular diagram of *Pba*1043, indicating predicted genomic islands. Circular diagram indicating, on the chromosome of *Pba*1043, the locations of annotated horizontally-acquired islands (HAI); aCGH predictions of *Pba*1043 genomic islands that do not have orthologues in *Pba*1039 (Pba1039I), *Pcc*193 (PccI), or *Dda*3937 (DdaI), or that are present only in *Pba* strains (PbaI); and predictions of divergent base composition made by alien_hunter (labelled as IVOM). Chromosome features are coloured by functional classification. Predicted HAIs coincide with many of the aCGH-predicted islands that themselves coincide with the predictions of divergent base composition made by alien_hunter. The *Dda*I islands are numerous and distributed in clusters around the chromosome. Most islands not predicted to be found in *Pcc*193 are also not predicted to be found in *Dda*3937. The diagram was constructed using GenomeDiagram [71].

HMM analysis predicted 165 islands (1179 CDS) from *Pba*1043 to have no orthologues in *Dda*3937, 60 islands (440 CDS) to have no orthologues in *Pcc*193, and 17 islands (198 CDS) to have no orthologues in *Pba*1039. This method also identified 16 islands (169 CDS) that were unique to *Pba*1043 only, and a further 40 islands (231 CDS) to be present only in *Pba*1043 and *Pba*1039. The count of genomic islands and CDS with no orthologue in the comparator diminished as the evolutionary distance from the last common ancestor of *Pba*1043 to the comparator decreased. These islands are illustrated in Figure 4 and Figure S6, and described in detail in Tables S1, S2, S3, S4 and S5.

We considered those CDS that are present in *Pba*1043 but that do not have orthologues in the most recently diverged organism in this study: *Pba*1039, to reflect either recent acquisitions in *Pba*1043 or recent losses in *Pba*1039. These CDS are a putative *Pba*1043-specific ‘accessory’ genome, and mostly comprise hypothetical proteins and phage-related sequences, located in 17 islands on the *Pba*1043 genome (Table S1; islands prefixed Pba1039I).

Fifty-six islands of *Pba*1043 CDS were predicted to be present only in *Pba*1043, or to be common to both *Pba* strains, but not to have orthologues in either *Dda*3937 or *Pcc*193. These are likely to represent genes encoding functions that biochemically distinguish *Pba* from its near evolutionary relatives. Such sequences included CDS encoding coronafacic acid synthesis (*cfa*), phenazine antibiotic synthesis (*ehp*), and various multidrug resistance genes. Several of these CDS, in particular those for the synthesis of coronafacic acids (CFA) have also previously been shown experimentally to contribute to virulence in *Pba*1043 [22]. These CDS were predicted to be components of the putative *Pba*-specific ‘accessory’ genome, and some examples are summarised in Table 3. A substantial minority of these CDS were annotated only as hypothetical proteins in their public sequence database submissions (Table S2).

**Table 3:** Predicted genomic islands of potential functional importance.

Island	Locus tags	Genomic location	Putative phenotype, and representative genes
PbaI5	*ECA0487*-*ECA0491*	563151-567396	phosphonate metabolism (*fom1*, *fom2*, *phnG*)
PbaI7	*ECA0600*-*ECA0610*	659712-682903	coronafacic acid synthesis (*cfa1-8B*, *cfl*)
PbaI13	*ECA1420*-*ECA1441*	1611940-1636204	polysaccharide and O-antigen synthesis (*rfb*, *nah*)
PbaI14	*ECA1477*-*ECA1481*	1673637-1678411	transcriptional regulators
PbaI22	*ECA2068*-*ECA2073*	2355814-2362568	permease and transporter
PbaI27	*ECA2294*-*ECA2295*	2599459-2602654	glycosyl transferase
PbaI33	*ECA2693*-*ECA2705*	3028797-3040751	polyketide synthase, phenazine synthesis (*ehpA-G*, *ehpR*)
PbaI39	*ECA2933*-*ECA2936*	3278434-3281122	nitrogen fixation (*nifQ*)
PbaI41	*ECA2972*-*ECA2982*	3322878-3332514	multidrug efflux (*emrE*)
PbaI44	*ECA3446*-*ECA3450*	3865038-3872587	multidrug efflux (*oprJ*, *mexBC*, *nfxB*)
PbaI55	*ECA4452*-*ECA4455*	4993334-5000237	hemin storage (*hms*)
PectoI7	*ECA0149*-*ECA0163*	169392-186544	lipopolysaccharide synthesis (*waa*)
PectoI25-27	*ECA0516*-*ECA0615*	590843-689677	mobile element (SPI7-like)
PectoI50	*ECA1089*-*ECA1107*	1217226-1255583	cell wall enzymes (*pel3, pehA*), Type I secretion
PectoI61	*ECA1485*-*ECA1490*	1680732-1681461	syringomycin-like non-ribosomal peptide synthase (*syrE*)
PectoI82	*ECA2111*-*ECA2118*	2398202-2426503	type III effectors (*dspE, hrpW*), agglutinins (*hecAB*)
PectoI96	*ECA2430*-*ECA2438*	2744003-2745227	virulence regultors (*rdgAB*)
PectoI122	*ECA3116*-*ECA3122*	3478243-3490068	type I restriction
PectoI129	*ECA3370*-*ECA3387*	3786824-3801913	pyoverdine biosynthesis (*pvc*)
PectoI151	*ECA4078*-*ECA4084*	4546706-4554057	octopine transport (*occQMP*)
PectoI153	*ECA4109*-*ECA4119*	4579773-4594107	siderophore synthesis / receptor

One-hundred and sixty-eight islands of *Pba*1043 CDS were predicted to have orthologues in both *Pcc*193 and *Pba*1039 but not in *Dda*3937, and thus represent a putative *Pectobacterium*-specific accessory genome. These islands are expected to include genes encoding functions that distinguish pectobacteria from *Dickeya* spp., and were found to contain CDS encoding PCWDE (*pel* and *peh*), a syringomycin-like NRPS (*syr*), siderophore biosynthesis (*pvc*) and octopine transport (*occ*).

### Sixteen of Seventeen Previously Annotated, Horizontally Acquired Islands from *Pba*1043 Are Coincident with Islands of CDS with No Predicted Orthologue in *Pcc*193 and/or *Dda*3937

Seventeen putative horizontally acquired islands (HAI1-HAI17) were identified in manual curation of the *Pba*1043 genome on the basis of evidence such as divergent base composition and the presence of flanking insertion sequences [22]. Of these, all but HAI1 coincided with at least one island identified by aCGH, and most include genes with putative or demonstrated roles in pathogenesis and niche adaptation [38] (Table 4).

**Table 4:** Horizontally acquired islands (HAI) previously identified in *Pba*1043.

HAI	Locus tags	*Pba*1039 Islands	*Pcc*193 Islands	*Dda*3937 Islands	Putative Phenotypes
HAI1	*ECA0499-ECA0510*	-	-	-	Capsular polysaccharide biosynthesis
HAI2	*ECA0516-ECA0614*	-	PccI6-PccI7	DdaI24	Polyketide phytotoxin *cfa*, SPI7
HAI3	*ECA0665-ECA0678*	-	PccI8	DdaI27	Phage genes
HAI4	*ECA1054-ECA1067*	Pba1039I2-Pba1039I3	PccI12	DdaI44	Phage genes/integrases
HAI5	*ECA1417-ECA1443*	-	PccI14	DdaI55	Exopolysaccharide and O-antigen biosynthesis (*rfb*, *nah*)
HAI6	*ECA1446-ECA1488*	-	PccI15	DdaI56-DdaI58	Syringomycin-like NRPS (*syrE*)
HAI7	*ECA1598-ECA1679*	Pba1039I4-Pba1039I6	PccI18	DdaI62	Integrases, type IV secretion, arsenate resistance (*ars*)
HAI8	*ECA2045-ECA2182*	-	PccI24-PccI28	DdaI76-DdaI84	Type III secretion (*hrp*), agglutinins (*hecAB*)
HAI9	*ECA2598-ECA2637*	Pba1039I10-Pba1039I11	PccI34-PccI35	DdaI100-DdaI101	P2 family prophage
HAI10	*ECA2694-ECA2705*	-	PccI36	DdaI103	Phenazine antibiotic biosynthesis (*ehp*)
HAI11	*ECA2750-ECA2759*	-	PccI37	DdaI104	Phage genes
HAI12	*ECA2850-ECA2879*	Pba1039I12	PccI38-PccI40	DdaI107-DdaI108	Hypothetical, putative type VI substrate (*vgrG*)
HAI13	*ECA2889-ECA2921*	Pba1039I13	PccI41	DdaI109	Putative integrated plasmid
HAI14	*ECA2922-ECA3000*	Pba1039I14	PccI42-PccI44	DdaI109-DdaI113	Nitrogen fixation (*nif*)
HAI15	*ECA3262-ECA3270*	-	-	DdaI122	Agglutination/adhesion (*aggA*)
HAI16	*ECA3378-ECA3460*	-	PccI45-PccI48	DdaI124-DdaI128	Multidrug resistance (*mex*-*opr*-*nfxB*)
HAI17	*ECA3695-ECA3742*	Pba1039I15	PccI49-PccI51	DdaI137-DdaI140	Prophage

Two of these islands, HAI1 (capsular polysaccharide biosynthesis) and HAI15 (type I secretion) were predicted to be entirely or substantially conserved in all organisms examined in this study. If the shared presence of each of these two islands is the result of horizontal gene transfer, then the most parsimonious inference is that acquisition occurred in a common ancestor of all three species, rather than as independent transfer events in each organism.

Two HAIs were predicted to have substantial orthologues only within the pectobacteria: the portion of HAI2 that is homologous with the SPI-7 pathogenicity island (PAI) flanking the coronafacic acid synthesis genes (the *cfa* genes themselves have no orthologues in *Pcc*193), and HAI6, which encodes a syringomycin-like NRPS. A parsimonious explanation for this distribution might be that these islands were acquired after the divergence of *Dickeya* and *Pectobacterium* spp. but before the divergence of *Pcc* and *Pba* species; alternatively, there may have been loss of these islands in the *Dickeya* lineage. However, the PAI itself has been observed in several unrelated bacterial genomes, and found to contain multiple alternative functional ‘payloads’ in those cases [53],[54]. As the PAI genes, but not their *cfa* ‘cargo’ were predicted to be present in *Pcc*193, it may be that there has been independent acquisition of this sequence in *Pba* and *Pcc*, carrying alternative payloads in each case. This may be determined by sequencing of that region in *Pcc*193.

Similarly, five HAIs (HAI3, HAI5, HAI10, HAI11 and HAI12) were found only in the two *Pba* strains either substantially, or in their entirety (Table 4; Table S2). These are expected to have been acquired after the divergence of *Pba* from *Pcc*. Amongst the gene functions carried by these HAIs are lipopolysaccharide biosynthesis (*rfb*) and phenazine antibiotic synthesis (*ehp*).

A further five HAIs (HAI4, HAI7, HAI9, HAI13, and HAI17) appeared to be substantially or entirely unique to *Pba*1043, but these almost exclusively encode for phage-related sequences, and hypothetical proteins. These were presumably recently acquired, subsequent to the divergence of strain SCRI1043 from strain SCRI1039.

HAI14, which putatively encodes nitrogen fixation function, is anomalous in that it was predicted to have a substantial number of orthologues in both *Pba* strains, and in *Dda*3937, but to have far fewer orthologues in *Pcc*193. The most parsimonious explanation for this distribution is that the common ancestor of *Dickeya* and *Pectobacterium* possessed this capability for nitrogen fixation, and that this has been progressively lost in the *Pcc*193 lineage. Alternatively, nitrogen-fixing ability may have been acquired independently in both *Dickeya* and *Pba* lineages.

### Predicted *Pba*1043-Specific Genomic Islands Are Associated with Regions of Divergent Genome Composition

The software package alien_hunter [42] was used to identify regions of putative HGT in the *Pba*1043 chromosome. An empirical statistical method was used to determine whether there was a significant association between *Pba*1043 CDS without predicted orthologues in each comparator species and regions of putative HGT as predicted by alien_hunter.

In total, alien_hunter identified regions of putative HGT overlapping 731 CDS in *Pba*1043. These included 118/173 CDS that were predicted by aCGH to be specific to *Pba*1043 (Z-score 5.54; P<0.0001); 254/400 CDS predicted to be specific to *Pba* strains (Z-score: 9.08, P<0.0001); 256/440 *Pba*1043 CDS predicted to have no orthologue in *Pcc*193 (Z-score: 8.47, P<0.0001); and 463/1179 CDS predicted to be have no orthologue in *Dda*3937 (Z-score: 9.03, P<0.0001). This indicates a significant tendency for *Pba*1043 CDS that are predicted to have no orthologue in one or more comparator organisms to be located within the regions of divergent base composition predicted by alien_hunter. This is consistent with the hypothesis that the composition of the ‘accessory’ genome of *Pba*1043 is greatly influenced by horizontal gene transfer.

A similar statistically significant association of predicted islands of CDS in *Pba*1043 predicted to have no orthologue in at least one comparator organism was observed with predicted regions of putative HGT identified by alien_hunter. In total, 11/16 (Z-score: 3.86, P<0.0001) *Pba*1043-specific islands; 32/56 (Z-score: 6.29, P<0.0001) *Pba*-specific islands; 32/60 (Z-score: 5.72, P<0.0001) islands predicted to have no orthologue in *Pcc*193; and 50/165 (Z-score: 2.39, P<0.01) islands predicted to have no orthologue in *Dda*3937 were found to overlap with the regions of putative HGT identified by alien_hunter.

It is particularly notable that nearly three-quarters of all *Pba*1043-specific islands also overlapped at least one region of divergent base composition predicted by alien_hunter. This is consistent with the proposal that these islands have been acquired through lateral gene transfer subsequent to divergence of *Pba*1043 and *Pba*1039 from their most recent common ancestor, suggesting a dynamic genome plasticity that persists and distinguishes between *Pba* strains [22].

### Several Predicted Genomic Islands Are Enhanced for CDS with Reciprocal Best Hits in Plant-Associated Bacteria, But Not in Animal-Pathogenic Enterobacteria

Each CDS in the *Pba*1043 chromosome was classified according to whether a putative protein orthologue was found in completely sequenced plant-associated bacteria (PAB), or in completely sequenced animal-pathogenic enterobacteria (APE) [38]. Those CDS that have at least one such orthologue in PAB, but none in APE were considered potentially to encode a biochemical function that is useful to a plant-associated lifestyle, but likely not to an animal-associated lifestyle. A similar inference may be drawn for CDS for which the *Pba*1043 sequence shares significantly greater identity with its most similar PAB orthologue than it does with the APE orthologue. As *Pba*1043 shares a more recent common ancestor with APE such as *Yersinia* spp. and *E. coli* strains, such a distribution of orthologous sequences may also imply acquisition by HGT.

An empirical statistical test was performed to determine whether genomic islands in *Pba*1043 identified by aCGH were enhanced for such CDS. A significant enhancement was seen for 6/56 *Pba*-specific islands, 6/60 islands with no orthologue in *Pcc*193, and 9/165 islands with no orthologue in *Dda*3937 (all tests Z-score>3.0; P<0.001; Table S6). These islands may therefore represent functions that are not only likely to have been acquired by lateral gene transfer, but may also be specific to a plant-associated, and not a generalist or animal-associated, lifestyle. Islands identified in this way include PbaI7, which contains genes that encode for coronafacic acid synthesis, and also a number of hypothetical proteins (see Discussion).

This partitioning of sequences between ‘core’ and ‘accessory’ regions of the bacterial genome, such that variable regions are enhanced for strain- or niche-specific functions has also been observed for other pathogenic bacteria, including *P. syringae* [9],[39], and appears to be a common strategy for the evolution of these organisms.

## Discussion

### Array CGH Does Not Necessarily Distinguish between ‘Present’ and ‘Absent or Divergent’ Sequences in a Comparator Organism

Microarray comparative genomic hybridisation (aCGH) is a valuable technique for rapidly, and relatively inexpensively, obtaining comparative genomic data for bacterial strains in a high-throughput manner. However, aCGH has inherent limitations that restrict the applicability of the method, and the information that can be obtained. Foremost is that an aCGH experiment is only able to identify which reference probe sequences do or do not hybridise well to gDNA from a comparator organism. In particular, aCGH is unable positively to identify sequences that are present in the comparator gDNA but that are absent from the reference or otherwise unrepresented in the probe set. Thus aCGH is unable to reflect sequences that are unique to the comparator organism. This may be overcome to some degree by the use of arrays that contain probes not only to the reference organism, but also to other related organisms, as proposed in [5]. Here, the wider the scope of the probes beyond the reference organism alone, the greater is the theoretical coverage of sequences that may be present in the comparator, but not in the reference organism. However, sequences that are unique to the comparator still cannot be disclosed by this approach unless they are present on the array.

It is commonly assumed that aCGH cannot distinguish between sequences that are absent in the comparator gDNA, and those that are merely sufficiently divergent that they cannot hybridise to the array probe set [5],[8, etc]. However, to some degree these classifications are indistinguishable, as the statement that a sequence is ‘absent’ in a comparator can be equivalent to the statement that there no significant sequence similarity. The use of ‘divergent’ as a classifier is ambiguous and potentially misleading in these circumstances. It is also commonly assumed that the assessment of ‘absence or divergence’ reflects overall sequence similarity, and that a relationship between hybridisation and sequence similarity holds for intermediate levels of sequence identity, such that intermediate hybridisation strengths reflect an intermediate degree of sequence identity [13]. An important observation made in [44] was that, even for closely-related sequenced strains of *Camplylobacter jejuni*, the log ratio of each probe was not sufficient to make a positive prediction of percentage sequence identity. We confirm and extend this observation for SRE with Agilent arrays.

It is often intuitively expected that microarray probes will hybridise to comparator gDNA with a reduced signal, where the comparator sequence is not identical with its homologue in the reference. In all aCGH experiments hybridisation strength is a measurement taken at the reference probe and not across the full length of the sequence from either organism, unless the probe covers the full length of the sequence. Where sequence identity is not homogeneous across the full length of the sequence, or there is similarity between the probe and a non-homologous sequence, this expectation may break down. A comparable break down may occur if there is the possibility of a confounding interaction between hybridising reference and comparator gDNA to a probe. Circumstances in which sequence divergence at the probe hybridisation site is not representative of the overall divergence across the sequence are highly likely to occur, and even under the most favourable circumstances it is only possible to refer to the *apparent* absence or divergence of sequences in the comparator organism.

Most published approaches to interpretation of aCGH data assume that probes which hybridise strongly to comparator gDNA represent sequences that are present in the comparator organism, while those probes that do not hybridise well represent sequences that may be either absent or divergent in the comparator [e.g.13],[15],[16]. By careful analysis of aCGH data for bacteria with complete genome annotations, we have established that this reasoning, while intuitively plausible, may lead to erroneous conclusions. Our data support only a distinction between those sequences that are, and those that are not, orthologous in the comparator organism. In particular they do not support a distinction between putatively orthologous sequences in terms of their degree of sequence identity, using aCGH hybridisation data. That is, the two sets of putatively orthologous sequences that would be classified as ‘present’ or merely ‘divergent’ could not be distinguished by us in terms of their hybridisation scores or ratios, and therefore the two classes of ‘present’ and ‘divergent or absent’ sequences could also not be distinguished.

We recognise that the array platform itself may be a significant factor in the interpretation of hybridisation data. Our microarray spots were designed with probes of 60 nt in length, one for each CDS, and the *L. lactis* array data we studied was derived from arrays spotted with amplicons of variable length from 80–800 bp [13]. Previous aCGH studies have employed a number of alternative array constructions, including gene-length cDNA probes, cDNA probes of partial genes, but longer than 60 nt; Affymetrix arrays with multiple short (25 nt) probes per spot; and Agilent arrays with 60 nt probes [10],[12],[14],[55]. Our data demonstrate that conclusions about the relationship between sequence identity and array hybridisation drawn using a particular array technology do not necessarily hold for alternative technologies. Measurement and validation of this relationship is essential for correct interpretation of aCGH data, and should be performed for each array platform.

Hybridisation binding strength or ratio data may also be interpreted in terms of a thermodynamic model of probe binding to the comparator organism gDNA, as an alternative to our interpretation in terms of percentage sequence identity. This is a useful technique when applied to resequencing of strains that are very closely related to the reference, as deviations in hybridisation strength may be accommodated within the thermodynamic model, and sequence differences inferred from observed binding affinities, in terms of that model; it may thus be a better approximator to hybridisation strength than is sequence identity. However we do not use it here as our aim is to infer putative orthology, defined in terms of sequence identity, from hybridisation data. The appropriate measure of putative orthology in this case is sequence identity, and not inferred sequence composition based on a model of the thermodynamic properties of probe binding. An interpretation of measured hybridisation in terms of sequence identity, validated on known sequence data, is therefore the most direct and appropriate approach for this study. Also, a typical bacterial aCGH experiment may involve a comparator organism that displays considerably greater divergence than that which would normally be considered for resequencing or other circumstances in which a thermodynamic model would usually be applied. For example, in our genus-level method validation only 807/10280 (less than 10%) of *Pba* array probes make a best match (with BLASTN) to the *Dda* genome that covers the probe to within 5 bp of its length. This significant divergence is likely to induce significant uncertainty, and therefore additional error, in the relationship between base composition as inferred from a thermodynamic model, and the subsequent assignment of putative orthology.

### The HMM Predictor Robustly Extends the Working Range of aCGH Analysis to Comparison of Bacteria at the Genus Level

Array CGH has previously been applied, in the main, to closely related organisms; in bacteria, this has usually involved comparisons at the intra- or inter-species level [e.g.5],[6],[7],[9],[13],[45],[46],[48],[56]. In principle, as hybridisation affinity is expected to be influenced by sequence identity, and not by schemes of systematic classification, it should be possible to extend the technique with some success to comparisons between organisms with a more ancient last common ancestor. In particular, DNA-DNA hybridisation studies of *Pba*1043, *Pcc*193 and *Dda*3937, 16S rRNA analysis and phylogenetic considerations (data not shown) indicate genome-wide sequence similarity that justifies the use of aCGH to compare the genome complements of these organisms. In this study, we successfully applied our analysis method to comparison data for *Pba*1043 and *Dda*3937: bacteria that differed at the genus level.

### Normalisation Methods for aCGH

It has been noted by other groups that a high degree of sequence divergence between prokaryotes may obstruct aCGH approaches, on the grounds that no strong assumption may be made concerning the distribution of hybridisation ratios for a Lowess normalisation step. Extension of aCGH to more distant comparisons has previously been attempted by modification of the normalisation method used on the array data, such as supervised Lowess (S-LOWESS) [5],[13]. However, we note that Lowess and many other array normalisation methods employ a null hypothesis which assumes that, for a significant proportion of probes, the hybridisation strengths of reference and comparator sequences are random variables drawn from the same distribution. This is a reasonable assumption when applied to isogenic data, such as bacterial mutants, these normalisation operations preserve differences in transcriptional expression while reducing systematic error, as the applied correction of normalisation is valid for the great majority of probes. It is not such a reasonable assumption for aCGH.

Normalisation methods such as Lowess may be useful for aCGH, on the condition that the reference and comparator diverged sufficiently recently, as the proportion of probes that do not conform to the underlying assumptions is likely to be small. This restricts the applicability of aCGH when using these normalisation approaches. However, in cases where the reference and comparator organisms do not share such a recent common ancestor, as for the *Pba*1043:*Dda*3937 comparison in which a strict majority of CDS do not have identifiable nucleotide RBH between the organisms (Figure S1), the underlying assumptions of Lowess normalisation fail for the majority of probes.

Subset modifications of Lowess have proven effective on within-species strain comparisons, but require the prior identification of conserved genes, and the assumption that the derived correction is applicable even to the majority of divergent sequences [5],[13]. Therefore in this study we used the nonparametric normalisation method of quantile normalisation (QN) to correct for systematic errors. QN requires no prior assumptions concerning the relatedness of reference and comparator sequences, and specifically makes no assumptions relating to sequence conservation. QN asserts only that the distribution of probe strengths is comparable across replicate arrays, which was established for our data in Figure 1.

The results of our consistency test validation indicate that measures of prediction quality for the interspecies *Pba*1043:*Dda*3937 comparison, though lower than that for the interstrain *Lactococcus* comparison, remain acceptable.

### The HMM aCGH Analysis Method Performs Better than GACK, MPP and Threshold Methods

Table 2 demonstrates that the HMM analysis method described in this paper outperforms GACK, MPP and threshold methods in identifying correctly those CDS in *Pba*1043 that do not have orthologues in *Dda*3937, and also those CDS in *L. lactis* MG1363 that do not have orthologues in IL1403. The consistency test of performance on the *Pba*1043:*Dda*3937 comparison suggests that GACK has a tendency to overpredict the number of reference sequences that have no orthologue in the comparator, which supports previous observations made using this method [5].

The HMM approach applied herein makes one straightforward improvement to the naïve threshold cutoff classification in that it incorporates information about the state of neighbouring CDS on the genome. Spatial data has previously been incorporated into methods applied to human copy number variation aCGH [18],[19],[20], in which the reference and comparator sequences may be assumed, accounting for noise, to be either so similar as to be near-identical, variant in signal by whole-number ratios, or absent altogether. Bacterial comparative genomic aCGH data also represents sequences that may be nearly identical, occur as copy-number variations, or absent altogether. However, the observed degree of sequence variation in bacterial comparative genomics is very high, and bacterial comparative data also has the potential to include tens to thousands of sequences that may be orthologous or paralogous, and to vary in terms of sequence identity at 50% of their sequence or more. We have demonstrated that the relationship between sequence identity and array hybridisation is complex in this system, and while it was expected that using information about spatial organisation would improve predictive performance, as it has done in the human copy number aCGH problem domain, the magnitude of this improvement was not readily predictable.

The HMM applied here is first-order, and so is the simplest such adaptation that could be applied. Further refinements of the methodology may deliver enhanced predictive performance. Although the resulting improvement in performance over a naïve threshold metric is not as striking as the improvement in relation to GACK and MPP results, it is a consistently better predictor and demonstrates that the incorporation of spatial information about hybridisation scores improves predictive performance. The qualitatively different predictions of the threshold and HMM methods suggest that these approaches identify intersecting subsets of true positives, and that an ensemble approach may be a worthwhile progression of the method.

It is possible that a HMM with bivariate outcomes, or training data (representing cy3 and cy5 intensities) might improve predictive ability of the model. Other methods of identifying an optimal path through the HMM than the Viterbi algorithm are also available. However, our results demonstrate that a HMM with univariate outcome, and using the Viterbi algorithm, performs better than accepted and widely-used approaches on bivariate signal data, and is sufficient to demonstrate that the incorporation of spatial genomic information improves aCGH prediction on bacterial genome sequences.

It might also be interesting to predict membership of one of the populations (high, medium or low hybridisation putative orthologues) observed in Figure 1 for each probe. However, our interest in this study was potential improvements in the prediction of putative orthologues in comparator sequences using spatial information in a HMM, and not optimisation of the predictive HMM.

### Array CGH Results Obtained for *Pba*1043 Comparisons Support a Model of ‘Accessory’ Genome Acquisition by Continuing, Dynamic Genomic Island Transfer

The improvement over threshold-based prediction seen with the HMM suggests a detectable biological signal from the collocation of sequences in the reference that do not have an orthologue in the comparator, and supports the hypothesis that the ‘accessory’ genome is acquired in large part through genomic islands, rather than individual genes. Accordingly, the number of islands of *Pba*1043 CDS with no orthologue in the comparator organism was seen to increase with evolutionary distance to a common ancestor. At all evolutionary distances, the predicted islands showed a statistically significant association with regions of putative horizontal gene transfer, whether identified by manual annotation or by the alien_hunter software. Further statistically significant results were observed for the association of these islands with sequences that were putatively orthologous to sequences in plant-associated bacteria, but not in animal-pathogenic enterobacteria. Taken together, this evidence is strongly suggestive of the acquisition of functions specific to the niche of these plant-associated enterobacteria by horizontal transfer.

The identification of islands in *Pba*1043 that do not have orthologues in *Pba*1039 is evidence that this process of lateral gene transfer continues in the SRE. Most of the CDS in these islands appeared to be phage-related or to encode hypothetical proteins (Table 4). These may not themselves be critical to the phenotypic differences between strains, but nevertheless indicate a dynamic genome with the potential for acquisition of novel function. However, comparisons at the species and genus level reveal major differences in gene content that may reflect differences in the abilities of each organism to persist in the environment (particularly on plants), and to cause disease on susceptible host plants.

### Regions Identified as ‘Accessory’ Genome Are Associated with Virulence, Pathogenicity, and Persistence in Specific Environmental Niches

Perhaps the most notable of the accessory islands is that which includes the *cfa* genes encoding for coronafacic acid (CFA) synthesis. The ability to synthesise this compound appears to be limited to *Pba*, amongst the SRE. In the plant pathogen *Pseudomonas syringae*, CFA is coupled to coronamic acid (CMA), to produce the phytotoxin coronatine, which promotes disease through manipulation of plant defences [57]. CFA has also been demonstrated to be required for virulence in *Pba*1043, providing the first evidence for the involvement of phytotoxins in soft rot pathogenesis, although its precise role has yet to be determined [22],[36]. In *Pba*1043 the *cfa* gene cluster is carried on a pathogenicity island highly similar to PAIs found in *Pcc*193 and other bacterial pathogens. In other pathogens these lack the *cfa* cluster, but in its place carry other genes with a range of functions, some of which are known to contribute to disease development, such as SPI-7 in *Salmonella enterica* serovar Typhi that carries the Vi expolysaccharide cluster [53],[54].

Other putative phytotoxic PKS and NRPS, such as the PKS *ECA2694* and a syringomycin synthesis-like NRPS are observed to be components of the ‘accessory’ genomes of *Pba* and *Pectobacterium*, respectively. Syringomycin is produced by strains of *Pseudomonas syringae*, and is a virulence factor responsible for pore formation and nutrient leakage through the host cell membrane [57]. The structure of the compound produced by the putatively *Pectobacterium*-specific NRPS, and any role it may play in virulence are as yet unknown.

The type III secretion system (T3SS) and its translocated effectors promote virulence by the manipulation of host plant defence responses [29]. The T3SS structural apparatus encoded by the *hrp*/*hrc* gene cluster appears to be conserved in all four SRE tested. Our HMM predicts that a number of neighbouring effector and helper proteins (e.g. *dspEF* and *hrpW*), and agglutinins (*hecAB*) do not have orthologues in *Dda*3937 (island DdaI80). However, sequence comparisons between the genomes of *Pba*1043 and *Dda*3937 indicate that this result is a false positive, and the *Pba*1043 CDS do in fact have orthologues in *Dda*3937. Such false positives may be caused or exacerbated by a tendency to design microarray probes to divergent regions of the reference gene. The distribution of these effectors may be strain-dependent, as the EC16 strain of *Dickeya* appears not to possess *dspE* or *hrpW* in the region flanking the *hrp* cluster [58],[59].

Some putatively *Pectobacterium*-specific islands carry genes encoding PCWDE that are not present in *Dda*3937, such as pectate lyase (*pel3*) and polygalacturonase (*pehA*). These differences in PCWDE complement may reflect corresponding differences in environmental niche and/or host range [25]. Other islands carry siderophores similar to pyoverdine and aerobactin, which appear to be restricted to *Pectobacterium* spp. amongst the SRE tested. Neither of these siderophores yet have a demonstrated association with virulence in pectobacteria, but the siderophores chrysobactin and achromobactin, which are not produced by *Pba*1043, are known to be involved with virulence in *Dda*3937 [60],[61].

Several *Pba*-specific and *Pectobacterium*-specific islands contain CDS encoding functions that are known or appear to be associated with persistence in the environment, and particularly on plant roots. These functions include phenazine antibiotic production (*ehp*), multidrug resistance (*emr*, *opr*, *mex*, *nfx*), and octopine uptake (*occ*; see Table 3). Phenazine has been shown to target other microorganisms in competition for limited nutrient resources in the rhizosphere [62],[63]. Phenazine does not appear to be produced by *Pcc*193 or *Dda*3937, but other antibiotics, such as carbapenem, which is produced by some *Pcc* strains, may provide equivalent function [64]. The multidrug resistance proteins unique to *Pba* may provide protection against these compounds. Octopines are tumour-derived compounds produced by the plant using genes transferred during infection by *Agrobacterium tumefaciens*. The resulting opines, including octopine, are used as a source of nutrition by the bacterium. The octopine uptake CDS, which we find to be putatively *Pectobacterium*-specific, may reflect an ability to piggyback on *Agrobacterium* infection and tumour formation [65]. This is an intriguing possibility when taken in context with the observation of genes associated with nitrogen fixation in *Pba*.

The region encoding putative nitrogen fixation function in *Pba*1043 is unusual in that orthologues to key genes in this island are found in *Dda*3937, and predicted to be present in *Pba*1039, but not in *Pcc*193. This would appear to imply either that *Pcc*193 has lost the capacity to fix nitrogen, or that at least one independent acquisition event has occurred to confer this ability. Nitrogen fixation is critical to the nitrogen cycle, and to the soil and rhizosphere environments, in converting atmospheric nitrogen into ammonium compounds that can then be converted by other microorganisms into compounds that may be used by plants. The apparent absence of this capability in *Pcc*193 suggests that the ability to fix nitrogen is not essential for successful pathogenesis. However, the ability to fix nitrogen may promote establishment and persistence in the environment.

### High-Throughput Sequencing Studies Also Support the Broad Conclusions of Our aCGH Analysis

Our aCGH study indicated that HAIs in *Pba*1043 that were previously identified through manual curation [22] were found to be variably present in the SRE strains investigated (Table 4). A recent study that used 454 sequencing to compare the genome of *Pba*1043 to the pectobacteria *Pcc*WPP14 and *P. braziliensis* 1692 (*Pbr*1692) also observed variation in the presence of these HAIs amongst the pectobacteria [66]. HAI2, which in our analysis was found to be present in *Pba*1043 and *Pcc*193, was found to be entirely absent from *Pcc*WPP14 and *Pbr*1692, suggesting that its occurrence is sporadic among the pectobacteria. Similarly HAI17, which we predicted to be specific to *Pba*1043, was found to be present in both *Pcc*WPP14 and *Pbr*1692. These observations confirm the broad theme of our conclusions, that these organisms, though related, have a dynamic, plastic genome composition that results in large functional changes even at the strain level.

### What Are the Prospects for aCGH?

A recent study indicates that high-throughput sequencing (HTS; serial analysis of gene expression: SAGE) provides advantages over microarray technology for gene expression analysis, though the false discovery rate appeared to be greater for SAGE [67]. There are several additional disadvantages of aCGH that are overcome by modern HTS methods such as 454 or Solexa/Illumina sequencing. Aside from the question of whether probe hybridisation state is a reliable proxy of sequence identity, or even putative orthology - questions that can be answered directly by sequencing - unlike HTS, aCGH cannot disclose or describe novel sequences in the comparator organism [68],[69]. The cost of sequencing a bacterial genome by these methods is falling rapidly at the time of writing, and there may come a point where the cost of completely sequencing a comparator genome is less than that of carrying out the comparable aCGH experiment. Even before that point is reached, the additional information that HTS provides may be such that it justifies the additional cost of the technique. This area is still moving rapidly, but it has been argued that, for some approaches such as chromatin immunoprecipitation, microarray and HTS experiments complement each other, and the same may be true for aCGH [70].

## Supporting Information

Table S1 17 Pba1043 genomic islands predicted to have no orthologues in Pba1039.
(0.06 MB PDF)

Table S2 56 genomic islands predicted to have orthologues in Pba1043 and Pba1039, but no orthologues in either of Pcc193 or Dda3937.
(0.10 MB PDF)

Table S3 60 Pba1043 genomic islands predicted to have no orthologues in Pcc193, prefixed PccI.
(0.10 MB PDF)

Table S4 165 Pba1043 genomic islands predicted to have no orthologues in Dda3937, prefixed DdaI.
(0.23 MB PDF)

Table S5 168 Pba1043 genomic islands predicted to have no orthologues in Dda3937, but to have orthologues in both Pcc193 and Pba1039, prefixed PectoI.
(0.16 MB PDF)

Table S6 Islands of Pba1043 CDS predicted by aCGH to have be Pba-specific (PbaI), or to have no orthologues in either Pcc193 (PccI).
(0.05 MB PDF)

Figure S1 Scatter plot of percentage sequence identity for coding sequences in Pba1043 to Dda3937 by reciprocal best FASTA and BLASTN analyses.
(0.31 MB PDF)

Figure S2 Scatterplots of putative orthologue (RBH) nucleotide sequence identity.
(1.54 MB PDF)

Figure S3 Plot of predictions of the theshold and HMM methods as the threshold is varied in the aCGH experiment with Dda3937 against Pba1043 taking log transformed hybridisation ratios.
(0.23 MB PDF)

Figure S4 Plots of CDS from the reference organisms predicted to have or not to have a putative orthologue in the comparator organisms.
(0.10 MB PDF)

Figure S5 Plot of the Pba1043 CDS with and without a predicted orthologue in Pba1039 and Pcc193.
(0.09 MB PDF)

Figure S6 Linear diagram indicating, on the chromosome of Pba1043, locations of horizontally acquired islands, aCGH predictions of genomic islands and predicted divergent base composition by alien-hunter.
(19.93 MB AI)
