# Supplementary material for: Microarray Comparative Genomic Hybridisation Analysis Incorporating Genomic Organisation, and Application to Enterobacterial Plant Pathogens
Source: PLoS Comput Biol. 2009 Aug 21;5(8):e1000473. doi: 10.1371/journal.pcbi.1000473 (PMC2718846; doi:10.1371/journal.pcbi.1000473)
Supplement: Table S1 — 17 Pba1043 genomic islands predicted to have no orthologues in Pba1039. (0.06 MB PDF) [file pcbi.1000473.s001.pdf]

Supplementary Table 1: 17 *Pba*1043 genomic islands predicted to have no orthologues in *Pba*1039. These 17 islands, prefixed Pba1039I, represent a predicted *Pba*1043-specific accessory genome.

Pba1039I1
=========

ECA0410	467141	467654	YP_048535.1	-	putative kinase
ECA0413	469395	472884	YP_048536.1	-	hypothetical protein

Pba1039I2
=========

ECA1055	1182390	1182576	YP_049162.1	-	hypothetical protein
ECA1056	1182639	1183911	YP_049163.1	-	putative phage-related reverse transcriptase/maturase family protein
ECA1057	1183907	1185917	YP_049164.1	-	phage-related hypothetical protein
ECA1058	1186180	1186795	YP_049165.1	-	hypothetical protein
ECA1059	1187196	1188408	YP_049166.1	-	putative integrase
ECA1060	1188400	1189894	YP_049167.1	-	putative integrase
ECA1061	1189893	1191858	YP_049168.1	-	putative integrase

Pba1039I3
=========

ECA1066	1194709	1195108	YP_049173.1	-	hypothetical protein
ECA1067	1195300	1196644	YP_049174.1	-	hypothetical protein

Pba1039I4
=========

ECA1596	1851991	1853839	YP_049697.1	-	hypothetical protein
ECA1597	1853835	1854879	YP_049698.1	-	hypothetical protein
ECA1598	1855522	1856875	YP_049699.1	-	hypothetical protein
ECA1600	1857402	1858335	YP_049700.1	ardC	antirestriction protein
ECA1601	1858445	1858988	YP_049701.1	-	transposase (partial)
ECA1602	1859014	1859923	YP_049702.1	-	LysR-family transcriptional regulator
ECA1603	1860347	1860773	YP_049703.1	arsC	arsenate reductase
ECA1604	1860785	1862075	YP_049704.1	arsB	arsenical pump membrane protein
ECA1605	1862119	1862440	YP_049705.1	arsR	DNA-binding transcriptional repressor
ECA1606	1862526	1863225	YP_049706.1	arsH	arsenical resistance protein
ECA1607	1863342	1863630	YP_049707.1	-	hypothetical protein
ECA1608	1863737	1864484	YP_049708.1	mobC	mobilization protein
ECA1609	1864494	1866396	YP_049709.1	-	mobilization protein
ECA1610	1866853	1867036	YP_049710.1	-	hypothetical plasmid protein
ECA1611	1867159	1867465	YP_049711.1	-	hypothetical plasmid protein

Pba1039I5
=========

ECA1617	1871988	1873029	YP_049718.1	virB6	putative conjugal transfer protein
ECA1618	1873102	1873378	YP_049719.1	-	putative plasmid protein
ECA1619	1873389	1874118	YP_049720.1	virB5	putative conjugal transfer protein
ECA1620	1874141	1876889	YP_049721.1	virB4	putative conjugal transfer protein

Pba1039I6
=========

ECA1628	1881481	1882006	YP_049729.1	-	hypothetical protein
ECA1629	1883004	1883862	YP_049730.1	-	AraC-family transcriptional regulator
ECA1632	1886844	1888533	YP_049731.1	-	hypothetical protein
ECA1633	1888809	1889685	YP_049732.1	-	hypothetical protein
ECA1634	1890003	1890399	YP_049733.1	-	hypothetical protein
ECA1635	1890476	1890773	YP_049734.1	-	hypothetical protein
ECA1636	1890822	1891059	YP_049735.1	-	hypothetical protein
ECA1637	1891085	1891436	YP_049736.1	-	hypothetical protein
ECA1638	1891504	1891747	YP_049737.1	-	Hns-like DNA-binding protein
ECA1639	1891964	1893239	YP_049738.1	-	hypothetical protein
ECA1640	1893332	1893812	YP_049739.1	-	hypothetical protein
ECA1641	1893899	1894037	YP_049740.1	-	hypothetical protein
ECA1642	1894271	1894526	YP_049741.1	-	hypothetical protein
ECA1643	1894836	1895358	YP_049742.1	-	hypothetical protein
ECA1644	1895383	1896118	YP_049743.1	-	putative DNA-binding protein
ECA1645	1897720	1898485	YP_049744.1	-	hypothetical protein
ECA1646	1898491	1899034	YP_049745.1	-	hypothetical protein
ECA1647	1899337	1900024	YP_049746.1	-	hypothetical protein
ECA1648	1900097	1900667	YP_049747.1	-	hypothetical protein
ECA1649	1900958	1901330	YP_049748.1	-	hypothetical protein
ECA1650	1901395	1901848	YP_049749.1	-	hypothetical protein
ECA1651	1901912	1902167	YP_049750.1	-	hypothetical protein
ECA1652	1902247	1903087	YP_049751.1	-	transposase
ECA1653	1903089	1903356	YP_049752.1	-	transposase
ECA1654	1903581	1903959	YP_049753.1	-	hypothetical protein
ECA1655	1904591	1905143	YP_049754.1	-	putative lipoprotein
ECA1656	1905153	1905429	YP_049755.1	-	hypothetical protein
ECA1657	1905550	1907395	YP_049756.1	-	hypothetical protein
ECA1658	1908103	1909087	YP_049757.1	-	hypothetical protein
ECA1659	1909270	1910731	YP_049758.1	-	probable plasmid-related protein
ECA1660	1911407	1912160	YP_049759.1	-	hypothetical protein
ECA1661	1912379	1913045	YP_049760.1	-	hypothetical protein
ECA1662	1913611	1913905	YP_049761.1	-	hypothetical protein
ECA1663	1913933	1914218	YP_049762.1	-	hypothetical protein

ECA1664	1914323	1914683	YP_049763.1	-	putative lipoprotein
ECA1665	1914762	1915161	YP_049764.1	hns1	DNA-binding protein Hns
ECA1666	1915261	1915492	YP_049765.1	-	hypothetical protein
ECA1667	1915723	1916281	YP_049766.1	-	putative type IV pilin protein precursor
ECA1668	1916387	1917980	YP_049767.1	-	putative type IV prepilin
ECA1669	1918142	1919885	YP_049768.1	-	hypothetical protein
ECA1669A	1919881	1920076	YP_049769.1	-	hypothetical protein
ECA1670	1920491	1920980	YP_049770.1	-	hypothetical protein
ECA1671	1921647	1921920	YP_049771.1	-	hypothetical protein
ECA1672	1922528	1922717	YP_049772.1	-	hypothetical protein
ECA1672A	1922742	1922949	YP_049773.1	-	hypothetical protein
ECA1673	1923047	1923206	YP_049774.1	-	integrase (partial)
ECA1674	1923211	1923496	YP_049775.1	-	hypothetical protein
ECA1675	1923639	1923981	YP_049776.1	-	putative integrase (partial)
ECA1676	1923956	1924289	YP_049777.1	chpA	toxin ChpA
ECA1677	1924288	1924540	YP_049778.1	chpR	suppressor of growth inhibitory protein ChpA
ECA1678	1925153	1925486	YP_049779.1	-	hypothetical protein

Pba1039I7
=========

ECA2019	2291855	2294414	YP_050113.1	-	hypothetical protein
ECA2020	2294423	2297000	YP_050114.1	-	hypothetical protein

Pba1039I8
=========

ECA2307	2614332	2614647	YP_050402.1	-	putative phage-related protein
ECA2308	2614680	2615379	YP_050403.1	-	putative phage-related protein
ECA2309	2616137	2616857	YP_050404.1	-	putative phage-related protein
ECA2310	2616853	2617060	YP_050405.1	-	hypothetical protein
ECA2311	2617182	2617593	YP_050406.1	-	hypothetical protein

Pba1039I9
=========

ECA2499	2816170	2816962	YP_050591.1	-	hypothetical protein
ECA2500	2816961	2819196	YP_050592.1	-	putative DNA-binding protein
ECA2501	2819422	2820118	YP_050593.1	-	hypothetical protein
ECA2502	2820114	2820354	YP_050594.1	-	hypothetical protein
ECA2503	2820770	2820968	YP_050595.1	-	putative phage-related protein
ECA2504	2821763	2822402	YP_050596.1	-	resolvase
ECA2505	2823148	2823316	YP_050597.1	-	hypothetical protein
ECA2506	2823640	2824555	YP_050598.1	-	hypothetical protein

Pba1039I10
==========

ECA2603	2939281	2939590	YP_050694.1	-	putative phage tail protein E
ECA2604	2939610	2939751	YP_050695.1	-	putative phage tail protein E'
ECA2605	2939737	2942668	YP_050696.1	-	putative bacteriophage tail fiber protein T (tape measure)
ECA2606	2942680	2943172	YP_050697.1	-	putative bacteriophage tail fiber protein U
ECA2607	2943292	2943910	YP_050698.1	-	putative bacteriophage tail fiber assembly protein G
ECA2608	2943909	2945520	YP_050699.1	-	probable bacteriophage variable tail fiber protein H
ECA2609	2945516	2946122	YP_050700.1	-	putative phage tail protein I
ECA2610	2946114	2947011	YP_050701.1	-	phage baseplate assembly protein J
ECA2611	2946997	2947366	YP_050702.1	-	phage baseplate assembly protein W
ECA2612	2947362	2947944	YP_050703.1	-	baseplate assembly protein V
ECA2613	2947940	2948579	YP_050704.1	-	putative phage tail protein S
ECA2614	2948571	2949024	YP_050705.1	-	phage tail completion protein R
ECA2615	2949163	2949604	YP_050707.1	-	putative phage encoded host lysis, regulatory protein
ECA2616	2949600	2950143	YP_050708.1	-	hypothetical protein

Pba1039I11
==========

ECA2620	2951269	2952163	YP_050712.1	-	phage terminase, endonuclease small subunit M
ECA2621	2952214	2953264	YP_050713.1	-	major phage capsid protein N precursor
ECA2622	2953288	2954122	YP_050714.1	-	capsid scaffolding protein O
ECA2623	2954280	2956002	YP_050715.1	-	phage terminase, ATPase subunit P
ECA2624	2956003	2957050	YP_050716.1	-	phage capsid portal protein Q
ECA2625	2957425	2958115	YP_050717.1	-	phage DNA methylase
ECA2626	2958117	2958444	YP_050718.1	-	putative phage-related protein
ECA2627	2958536	2961047	YP_050719.1	-	putative phage replication protein A
ECA2628	2961046	2962075	YP_050720.1	-	conserved phage protein
ECA2629	2962067	2962916	YP_050721.1	-	phage DNA adenine methylase
ECA2630	2962912	2963812	YP_050722.1	-	hypothetical protein
ECA2631	2963808	2964042	YP_050723.1	-	hypothetical protein
ECA2632	2964112	2964448	YP_050724.1	-	hypothetical protein
ECA2633	2964528	2964654	YP_050725.1	-	hypothetical protein
ECA2634	2964650	2964851	YP_050726.1	-	phage-related hypothetical protein
ECA2635	2964847	2965210	YP_050727.1	-	phage regulatory protein

Pba1039I12
==========

ECA2874	3216323	3217214	YP_050965.1	-	hypothetical protein
ECA2875	3217206	3220539	YP_050966.1	-	putative ATP-binding protein
ECA2876	3220531	3221371	YP_050967.1	-	putative phage-related protein
ECA2877	3221521	3223375	YP_050968.1	-	hypothetical protein
ECA2878	3223377	3225771	YP_050969.1	-	putative signal transduction protein

Pba1039I13
==========

ECA2890	3237700	3238219	YP_050981.1	-	hypothetical protein
ECA2891	3238279	3239092	YP_050982.1	-	hypothetical protein

ECA2892	3239132	3239936	YP_050983.1	-	hypothetical protein
ECA2893	3241129	3241531	YP_050984.1	hns3	DNA-binding protein Hns
ECA2894	3241657	3242011	YP_050985.1	-	putative plasmid-related protein
ECA2895	3242010	3242289	YP_050986.1	-	hypothetical protein
ECA2896	3242491	3242674	YP_050987.1	-	hypothetical protein
ECA2897	3242707	3242992	YP_050988.1	-	hypothetical protein
ECA2898	3243721	3245125	YP_050989.1	-	putative plasmid mobilization protein
ECA2899	3245207	3246110	YP_050990.1	-	hypothetical protein
ECA2900	3246455	3247238	YP_050991.1	-	hypothetical protein
ECA2901	3248044	3248488	YP_050992.1	-	hypothetical protein
ECA2902	3248551	3248980	YP_050993.1	-	hypothetical protein
ECA2903	3249068	3249716	YP_050994.1	-	hypothetical protein
ECA2904	3249732	3250287	YP_050995.1	-	putative lipoprotein
ECA2905	3250921	3251299	YP_050996.1	-	hypothetical protein
ECA2906	3251566	3252769	YP_050997.1	-	hypothetical protein
ECA2907	3253050	3253389	YP_050998.1	-	hypothetical protein
ECA2908	3253485	3254250	YP_050999.1	-	putative plasmid replication protein
ECA2909	3255301	3255463	YP_051000.1	-	hypothetical protein
ECA2910	3255833	3256568	YP_051001.1	-	putative plasmid replication protein
ECA2911	3256753	3257866	YP_051002.1	-	putative DNA-binding protein
ECA2912	3257875	3258616	YP_051003.1	-	putative plasmid-related protein
ECA2913	3258797	3259049	YP_051004.1	-	putative DNA-binding protein
ECA2914	3259116	3259467	YP_051005.1	-	hypothetical protein
ECA2915	3259506	3259770	YP_051006.1	-	hypothetical protein
ECA2916	3259823	3260120	YP_051007.1	-	hypothetical protein
ECA2917	3260199	3260514	YP_051008.1	-	hypothetical protein
ECA2918	3260546	3260765	YP_051009.1	-	putative phage-related protein
ECA2919	3260914	3261778	YP_051010.1	-	hypothetical protein
ECA2920	3261770	3261941	YP_051011.1	-	hypothetical protein
ECA2921	3262079	3263492	YP_051012.1	-	hypothetical protein

Pba1039I14

==========

ECA2975	3325953	3326604	YP_051066.1	-	hypothetical protein
ECA2976	3326721	3327630	YP_051067.1	-	LysR-family transcriptional regulator
ECA2977	3327795	3328329	YP_051068.1	-	hypothetical protein

Pba1039I15

==========

ECA3696	4144979	4145402	YP_051784.1	-	putative phage regluatory protein
ECA3697	4145379	4145580	YP_051785.1	-	hypothetical protein
ECA3698	4145576	4146017	YP_051786.1	-	hypothetical protein
ECA3699	4146027	4146327	YP_051787.1	-	conserved hypothetical phage-related protein
ECA3700	4146417	4147026	YP_051788.1	-	conserved hypothetical phage-related protein
ECA3701	4147038	4147308	YP_051789.1	-	conserved hypothetical phage-related protein
ECA3702	4147335	4147593	YP_051790.1	-	hypothetical phage-related protein
ECA3703	4147595	4148747	YP_051791.1	-	conserved hypothetical phage-related protein
ECA3704	4148756	4150526	YP_051792.1	-	conserved hypothetical phage-related protein
ECA3705	4150535	4151444	YP_051793.1	-	conserved hypothetical phage-related protein
ECA3706	4151453	4151759	YP_051794.1	-	putative phage-related DNA-binding protein
ECA3707	4151811	4152000	YP_051795.1	-	putative phage-related DNA-binding protein
ECA3708	4152091	4152508	YP_051796.1	-	putative phage-related DNA-binding protein
ECA3709	4152526	4153063	YP_051797.1	-	putative phage-related membrane protein
ECA3710	4153097	4154042	YP_051798.1	-	putative phage-related lipoprotein
ECA3711	4154161	4155169	YP_051799.1	-	conserved hypothetical phage-related protein
ECA3712	4155171	4156353	YP_051800.1	-	putative phage-related membrane protein

Pba1039I16

==========

ECA3719	4159329	4160865	YP_051807.1	-	putative phage-related protein
ECA3720	4160864	4162358	YP_051808.1	-	putative phage-related protein
ECA3721	4162338	4163160	YP_051809.1	-	putative phage-related protein
ECA3722	4163156	4163606	YP_051810.1	-	putative phage-related protein
ECA3723	4163801	4164911	YP_051811.1	-	conserved phage-related protein
ECA3724	4164947	4165883	YP_051812.1	-	conserved phage-related protein

Pba1039I17

==========

ECA3730	4168785	4169310	YP_051818.1	-	major tail sheath protein
ECA3731	4169316	4169517	YP_051819.1	-	hypothetical protein
ECA3731A	4169657	4169960	YP_051820.1	-	putative phage-related protein
ECA3732	4170346	4172818	YP_051821.1	-	putative bacteriophage tail protein (Tape-measure)
ECA3733	4172817	4173702	YP_051822.1	-	putative phage-related protein
ECA3734	4173698	4173914	YP_051823.1	-	putative bacteriophage tail fibre protein
ECA3735	4173901	4175047	YP_051824.1	-	putative bacteriophage protein
ECA3736	4175043	4175637	YP_051825.1	-	putative bacteriophage baseplate protein
ECA3737	4175663	4176512	YP_051826.1	-	putative phage-related protein
