# Supplementary material for: Microarray Comparative Genomic Hybridisation Analysis Incorporating Genomic Organisation, and Application to Enterobacterial Plant Pathogens
Source: PLoS Comput Biol. 2009 Aug 21;5(8):e1000473. doi: 10.1371/journal.pcbi.1000473 (PMC2718846; doi:10.1371/journal.pcbi.1000473)
Supplement: Table S3 — 60 Pba1043 genomic islands predicted to have no orthologues in Pcc193, prefixed PccI. (0.10 MB PDF) [file pcbi.1000473.s003.pdf]

Supplementary Table 3: 60 *Pba*1043 genomic islands predicted to have no orthologues in *Pcc*193, prefixed PccI.

PccI1
=====

ECA0176	196153	196633	YP_048303.1	-	hypothetical protein
ECA0177	196649	197366	YP_048304.1	-	hypothetical protein

PccI2
=====

ECA0394	451995	452394	YP_048518.1	-	hypothetical protein
ECA0395	452394	452937	YP_048519.1	kptA	RNA 2'-phosphotransferase
ECA0396	453260	454961	YP_048520.1	-	putative haemolysin-like protein
ECA0397	454962	455337	YP_048521.1	-	hypothetical protein
ECA0398	455618	456002	YP_048522.1	-	hypothetical protein
ECA0399	456047	456269	YP_048523.1	-	hypothetical protein
ECA0399A	456304	456442	YP_048524.1	-	putative hemagglutinin-like protein (partial)
ECA0400	456425	456719	YP_048525.1	-	putative phage-related protein
ECA0401	457210	457861	YP_048526.1	-	hypothetical protein

PccI3
=====

ECA0410	467141	467654	YP_048535.1	-	putative kinase
ECA0413	469395	472884	YP_048536.1	-	hypothetical protein

PccI4
=====

ECA0423	479800	480919	YP_048541.1	-	hypothetical protein
ECA0424	480918	481917	YP_048542.1	-	hypothetical protein

PccI5
=====

ECA0487	563151	564084	YP_048605.1	fom1	phosphoenolpyruvate phosphomutase
ECA0488	564263	565418	YP_048606.1	fom2	phosphonopyruvate decarboxylase
ECA0489	565470	566364	YP_048607.1	-	putative 2-hydroxy-3-oxopropionate reductase
ECA0490	566364	566808	YP_048608.1	phnG	putative phosphonate metabolism protein
ECA0491	566823	567396	YP_048609.1	phnH	carbon-phosphorus lyase complex subunit

PccI6
=====

ECA0583	644957	645260	YP_048699.1	-	putative plasmid-related protein
ECA0584	645557	647429	YP_048700.1	-	restriction enzyme alpha subunit
ECA0585	647418	648405	YP_048701.1	-	restriction enzyme beta subunit

PccI7
=====

ECA0600	659712	661026	YP_048717.1	cfa8B	putative oxidoreductase
ECA0601	661109	661541	YP_048718.1	cfa8A	putative oxidoreductase
ECA0602	661604	667991	YP_048719.1	cfa7	type I polyketide synthase
ECA0603	667987	676129	YP_048720.1	cfa6	type I polyketide synthase
ECA0604	676142	677582	YP_048721.1	cfa5	coronafacic acid synthetase, ligase component
ECA0605	677578	678112	YP_048722.1	cfa4	coronafacic acid synthetase component
ECA0606	678108	679254	YP_048723.1	cfa3	Cfa-beta-ketoacylsynthase
ECA0607	679247	679745	YP_048724.1	cfa2	coronafacic acid dehydratase
ECA0608	679741	680017	YP_048725.1	cfa1	Cfa-acyl carrier protein
ECA0609	680118	681681	YP_048726.1	cfl	coronafacate ligase
ECA0610	681940	682903	YP_048727.1	-	LysR-family transcriptional regulator

PccI8
=====

ECA0665	739282	740689	YP_048780.1	-	putative phage integrase
ECA0666	740666	741473	YP_048781.1	-	hypothetical protein
ECA0667	741624	741816	YP_048782.1	-	hypothetical protein
ECA0668	741881	742790	YP_048783.1	-	hypothetical protein
ECA0669	742779	743127	YP_048784.1	-	hypothetical protein
ECA0670	743119	743425	YP_048785.1	-	hypothetical protein
ECA0671	743497	743797	YP_048786.1	-	putative bacteriophage derepression protein
ECA0672	743860	746539	YP_048787.1	-	putative phage-related protein
ECA0673	747114	747489	YP_048788.1	-	putative phage-related DNA binding protein
ECA0674	747552	747822	YP_048789.1	-	putative phage-related protein
ECA0675	748134	748305	YP_048790.1	-	hypothetical protein

PccI9
=====

ECA0777	848522	849212	YP_048889.1	-	hypothetical protein
ECA0778	849287	851009	YP_048890.1	-	hypothetical protein

PccI10
======

ECA0830	921447	921897	YP_048940.1	-	hypothetical protein
ECA0831	921905	922199	YP_048941.1	-	putative transposase (partial)
ECA0832	922239	922566	YP_048942.1	-	putative phage capsid protein (partial)
ECA0833	922584	922755	YP_048943.1	-	phage regulatory protein
ECA0834	923067	925512	YP_048944.1	-	hypothetical protein

PccI11
======

ECA0922	1029896	1030829	YP_049031.1	-	LysR-family transcriptional regulator
ECA0923	1030958	1031849	YP_049032.1	-	hypothetical protein

PccI12
======

ECA1054	1180861	1182331	YP_049161.1	-	putative integrase
ECA1055	1182390	1182576	YP_049162.1	-	hypothetical protein
ECA1056	1182639	1183911	YP_049163.1	-	putative phage-related reverse transcriptase/maturase family protein
ECA1057	1183907	1185917	YP_049164.1	-	phage-related hypothetical protein
ECA1058	1186180	1186795	YP_049165.1	-	hypothetical protein
ECA1059	1187196	1188408	YP_049166.1	-	putative integrase
ECA1060	1188400	1189894	YP_049167.1	-	putative integrase
ECA1061	1189893	1191858	YP_049168.1	-	putative integrase
ECA1062	1191838	1192258	YP_049169.1	-	hypothetical protein
ECA1063	1192338	1192806	YP_049170.1	-	hypothetical protein
ECA1064	1192860	1193283	YP_049171.1	-	hypothetical protein
ECA1065	1193408	1194704	YP_049172.1	-	hypothetical protein
ECA1066	1194709	1195108	YP_049173.1	-	hypothetical protein
ECA1067	1195300	1196644	YP_049174.1	-	hypothetical protein
ECA1068	1197375	1198215	YP_049175.1	-	hypothetical protein

PccI13
======

ECA1228	1399080	1400775	YP_049334.1	hybC	hydrogenase 2 large subunit
ECA1229	1400771	1401275	YP_049335.1	hybD	predicted maturation element for hydrogenase 2
ECA1230	1401258	1401732	YP_049336.1	hybE	hydrogenase 2-specific chaperone
ECA1231	1401791	1402802	YP_049337.1	hypE	hydrogenase isoenzyme formation protein
ECA1232	1402801	1403920	YP_049338.1	hypD	hydrogenase isoenzyme formation protein
ECA1233	1403919	1404198	YP_049339.1	hypC	hydrogenase isoenzymes formation protein
ECA1234	1404188	1405064	YP_049340.1	hypB	hydrogenase nickel incorporation protein HypB
ECA1235	1405065	1405419	YP_049341.1	hypA	hydrogenase nickel incorporation protein
ECA1236	1406109	1408089	YP_049342.1	hyfR	hydrogenase-4 transcriptional activator
ECA1237	1408140	1408623	YP_049343.1	hycI	hydrogenase 3 maturation protease
ECA1238	1408627	1409050	YP_049344.1	hyfJ	hydrogenase-4 component J
ECA1239	1409042	1409822	YP_049345.1	hyfI	hydrogenase-4 component I
ECA1240	1409818	1410370	YP_049346.1	hyfH	hydrogenase 4 subunit H
ECA1241	1410377	1412114	YP_049347.1	hyfG	hydrogenase-4 component G
ECA1242	1412100	1413714	YP_049348.1	hyfF	hydrogenase 4 subunit F

PccI14
======

ECA1420	1611940	1613374	YP_049526.1	rfbP	undecaprenyl-phosphate galactosephosphotransferase
ECA1421	1613497	1614457	YP_049527.1	rfbI	CDP-6-deoxy-delta-3,4-glucoseen reductase
ECA1422	1614551	1615325	YP_049528.1	rfbF	glucose-1-phosphate cytidylyltransferase
ECA1423	1615315	1616422	YP_049529.1	rfbG	CDP-glucose 4,6-dehydratase
ECA1424	1616421	1617735	YP_049530.1	rfbH	CDP-4-keto-6-deoxy-D-glucose-3-dehydratase
ECA1425	1617929	1618712	YP_049531.1	hpcH	putative 2,4-dihydroxyhept-2-ene-1,7-dioic acid aldolase
ECA1426	1618721	1620518	YP_049532.1	-	putative thiamine pyrophosphate enzyme
ECA1427	1620523	1621459	YP_049533.1	-	putative dTDP-glucose 4,6-dehydratase
ECA1428	1621486	1622377	YP_049534.1	nahO	acetaldehyde dehydrogenase
ECA1429	1622381	1623413	YP_049535.1	nahM	4-hydroxy-2-ketovalerate aldolase
ECA1430	1623415	1624471	YP_049536.1	-	putative dTDP-glucose 4-6-dehydratase
ECA1431	1624708	1626028	YP_049537.1	rfbX	putative O-antigen transporter
ECA1432	1625990	1626770	YP_049538.1	-	putative glycosyl transferase
ECA1433	1626766	1627393	YP_049539.1	-	putative acyl transferase
ECA1434	1627408	1628554	YP_049540.1	-	putative glycosyl transferase
ECA1435	1628550	1629753	YP_049541.1	rfc	putative O-antigen polymerase
ECA1436	1629785	1630895	YP_049542.1	rfbU	putative glycosyl transferase
ECA1437	1631013	1631931	YP_049543.1	rfbN	O antigen biosynthesis rhamnosyltransferase rfbn
ECA1438	1631951	1633361	YP_049544.1	rfbM	mannose-1-phosphate guanylyltransferase
ECA1439	1633373	1634744	YP_049545.1	rfbK	phosphomannomutase
ECA1440	1634819	1635671	YP_049546.1	rfbD	dTDP-4-dehydrorhamnose reductase
ECA1441	1635667	1636204	YP_049547.1	rfbC	dTDP-6-deoxy-D-glucose-3,5 epimerase

PccI15
======

ECA1477	1673637	1673904	YP_049582.1	-	hypothetical protein
ECA1478	1674048	1674948	YP_049583.1	-	AraC-family transcriptional regulator
ECA1479	1675100	1676003	YP_049584.1	-	LysR-family transcriptional regulator
ECA1480	1676094	1677111	YP_049585.1	-	putative zinc-binding oxidoreductase
ECA1481	1677772	1678411	YP_049586.1	-	hypothetical protein

PccI16
======

ECA1521	1770115	1771012	YP_049623.1	-	hypothetical protein
ECA1522	1771276	1771549	YP_049624.1	-	hypothetical protein

PccI17
======

ECA1584	1837756	1837906	YP_049685.1	-	hypothetical protein
ECA1585	1837902	1839441	YP_049686.1	-	hypothetical protein
ECA1586	1840175	1840934	YP_049687.1	-	probable short chain dehydrogenase
ECA1587	1840981	1841674	YP_049688.1	-	probable glutathione-S-transferase
ECA1588	1841726	1842110	YP_049689.1	-	hypothetical protein
ECA1589	1842240	1847037	YP_049690.1	lhr	putative ATP-dependent helicase Lhr
ECA1590	1847044	1847386	YP_049691.1	-	hypothetical protein
ECA1591	1847461	1848211	YP_049692.1	-	hypothetical protein
ECA1592	1848238	1849141	YP_049693.1	-	putative acetyl-hydrolase
ECA1593	1849383	1850334	YP_049694.1	-	AraC-family transcriptional regulator
ECA1594	1850489	1850948	YP_049695.1	-	hypothetical protein
ECA1595	1851096	1851453	YP_049696.1	-	hypothetical protein
ECA1596	1851991	1853839	YP_049697.1	-	hypothetical protein

ECA1597 1853835 1854879 YP_049698.1 - hypothetical protein
ECA1598 1855522 1856875 YP_049699.1 - hypothetical protein
PccI18
======
ECA1628 1881481 1882006 YP_049729.1 - hypothetical protein
ECA1629 1883004 1883862 YP_049730.1 - AraC-family transcriptional regulator
ECA1632 1886844 1888533 YP_049731.1 - hypothetical protein
ECA1633 1888809 1889685 YP_049732.1 - hypothetical protein
ECA1634 1890003 1890399 YP_049733.1 - hypothetical protein
ECA1635 1890476 1890773 YP_049734.1 - hypothetical protein
ECA1636 1890822 1891059 YP_049735.1 - hypothetical protein
ECA1637 1891085 1891436 YP_049736.1 - hypothetical protein
ECA1638 1891504 1891747 YP_049737.1 - Hns-like DNA-binding protein
ECA1639 1891964 1893239 YP_049738.1 - hypothetical protein
ECA1640 1893332 1893812 YP_049739.1 - hypothetical protein
ECA1641 1893899 1894037 YP_049740.1 - hypothetical protein
ECA1642 1894271 1894526 YP_049741.1 - hypothetical protein
ECA1643 1894836 1895358 YP_049742.1 - hypothetical protein
ECA1644 1895383 1896118 YP_049743.1 - putative DNA-binding protein
ECA1645 1897720 1898485 YP_049744.1 - hypothetical protein
ECA1646 1898491 1899034 YP_049745.1 - hypothetical protein
ECA1647 1899337 1900024 YP_049746.1 - hypothetical protein
ECA1648 1900097 1900667 YP_049747.1 - hypothetical protein
ECA1649 1900958 1901330 YP_049748.1 - hypothetical protein
ECA1650 1901395 1901848 YP_049749.1 - hypothetical protein
ECA1651 1901912 1902167 YP_049750.1 - hypothetical protein
ECA1652 1902247 1903087 YP_049751.1 - transposase
ECA1653 1903089 1903356 YP_049752.1 - transposase
ECA1654 1903581 1903959 YP_049753.1 - hypothetical protein
ECA1655 1904591 1905143 YP_049754.1 - putative lipoprotein
ECA1656 1905153 1905429 YP_049755.1 - hypothetical protein
ECA1657 1905550 1907395 YP_049756.1 - hypothetical protein
ECA1658 1908103 1909087 YP_049757.1 - hypothetical protein
ECA1659 1909270 1910731 YP_049758.1 - probable plasmid-related protein
ECA1660 1911407 1912160 YP_049759.1 - hypothetical protein
ECA1661 1912379 1913045 YP_049760.1 - hypothetical protein
ECA1662 1913611 1913905 YP_049761.1 - hypothetical protein
ECA1663 1913933 1914218 YP_049762.1 - hypothetical protein
ECA1664 1914323 1914683 YP_049763.1 - putative lipoprotein
ECA1665 1914762 1915161 YP_049764.1 hns1 DNA-binding protein Hns
ECA1666 1915261 1915492 YP_049765.1 - hypothetical protein
ECA1667 1915723 1916281 YP_049766.1 - putative type IV pilin protein precursor
ECA1668 1916387 1917980 YP_049767.1 - putative type IV prepilin
ECA1669 1918142 1919885 YP_049768.1 - hypothetical protein
ECA1669A 1919881 1920076 YP_049769.1 - hypothetical protein
ECA1670 1920491 1920980 YP_049770.1 - hypothetical protein
ECA1671 1921647 1921920 YP_049771.1 - hypothetical protein
ECA1672 1922528 1922717 YP_049772.1 - hypothetical protein
ECA1672A 1922742 1922949 YP_049773.1 - hypothetical protein
ECA1673 1923047 1923206 YP_049774.1 - integrase (partial)
ECA1674 1923211 1923496 YP_049775.1 - hypothetical protein
ECA1675 1923639 1923981 YP_049776.1 - putative integrase (partial)
ECA1676 1923956 1924289 YP_049777.1 chpA toxin ChpA
ECA1677 1924288 1924540 YP_049778.1 chpR suppressor of growth inhibitory protein ChpA
ECA1678 1925153 1925486 YP_049779.1 - hypothetical protein
PccI19
======
ECA1732 1976823 1980243 YP_049832.1 - hypothetical protein
ECA1733 1980554 1981667 YP_049833.1 vioA nucleotide sugar transaminase
ECA1734 1981772 1982474 YP_049834.1 - hypothetical protein
ECA1735 1982476 1983436 YP_049835.1 - hypothetical protein
ECA1736 1983435 1984404 YP_049836.1 - hypothetical protein
ECA1737 1984403 1984844 YP_049837.1 - putative acetyltransferase
PccI20
======
ECA1836 2081519 2083361 YP_049933.1 edd phosphogluconate dehydratase
ECA1837 2083657 2083858 YP_049934.1 - hypothetical protein
PccI21
======
ECA1848 2095151 2096741 YP_049945.1 aglA PTS system, alpha-glucoside-specific IIBC component
ECA1849 2097008 2097755 YP_049946.1 - GntR-family transcriptional regulator
PccI22
======
ECA1919 2175614 2177036 YP_050016.1 - putative electron transport protein
ECA1920 2177045 2177765 YP_050017.1 - hypothetical protein
PccI23
======
ECA2019 2291855 2294414 YP_050113.1 - hypothetical protein
ECA2020 2294423 2297000 YP_050114.1 - hypothetical protein
PccI24
======
ECA2046 2325746 2328827 YP_050141.1 - putative bifunctional enzyme including aminotransferase and
chitin synthase

ECA2047	2328860	2330060	YP_050142.1	-	hypothetical protein
ECA2048	2330076	2332041	YP_050143.1	-	hypothetical protein
ECA2049	2332155	2332908	YP_050144.1	-	hypothetical protein

PccI25
======

ECA2068	2355814	2357275	YP_050163.1	-	putative permease
ECA2069	2357360	2358035	YP_050164.1	-	TetR-family transcriptional regulator
ECA2070	2358132	2359305	YP_050165.1	-	major facilitator family transporter
ECA2071	2359588	2360806	YP_050166.1	-	putative cytochrome P450
ECA2072	2360795	2361239	YP_050167.1	-	flavodoxin
ECA2073	2361326	2362568	YP_050168.1	-	probable oxidoreductase

PccI26
======

ECA2119	2426504	2426837	YP_050214.1	-	hypothetical protein
ECA2120	2426873	2427218	YP_050215.1	-	hypothetical protein

PccI27
======

ECA2126	2428960	2429380	YP_050221.1	-	hypothetical protein
ECA2127	2429381	2429813	YP_050222.1	-	hypothetical protein
ECA2128	2430107	2431115	YP_050223.1	-	hypothetical protein
ECA2129	2431181	2431634	YP_050224.1	-	hypothetical protein
ECA2130	2431736	2432021	YP_050225.1	-	hypothetical protein
ECA2131	2432246	2432537	YP_050226.1	-	hypothetical protein
ECA2132	2432550	2433018	YP_050227.1	-	hypothetical protein

PccI28
======

ECA2175	2479529	2479928	YP_050270.1	-	hypothetical protein
ECA2176	2479938	2480340	YP_050271.1	-	hypothetical protein
ECA2178	2480846	2481182	YP_050272.1	-	prophage integrase (partial)
ECA2179	2481463	2481994	YP_050273.1	-	hypothetical protein
ECA2180	2481996	2484267	YP_050274.1	-	hypothetical protein
ECA2181	2484488	2484836	YP_050275.1	-	Bacteriophage polarity suppression protein

PccI29
======

ECA2232	2534899	2535796	YP_050326.1	-	LysR-family transcriptional regulator
ECA2233	2535824	2536745	YP_050327.1	-	probable hydrolase
ECA2234	2536812	2537412	YP_050328.1	-	hypothetical protein
ECA2235	2537429	2537795	YP_050329.1	-	putative transcriptional regulator

PccI30
======

ECA2294	2599459	2601298	YP_050389.1	-	putative glycosyl transferase
ECA2295	2601616	2602654	YP_050390.1	-	AraC-family transcriptional regulator

PccI31
======

ECA2307	2614332	2614647	YP_050402.1	-	putative phage-related protein
ECA2308	2614680	2615379	YP_050403.1	-	putative phage-related protein
ECA2309	2616137	2616857	YP_050404.1	-	putative phage-related protein
ECA2310	2616853	2617060	YP_050405.1	-	hypothetical protein
ECA2311	2617182	2617593	YP_050406.1	-	hypothetical protein

PccI32
======

ECA2499	2816170	2816962	YP_050591.1	-	hypothetical protein
ECA2500	2816961	2819196	YP_050592.1	-	putative DNA-binding protein
ECA2501	2819422	2820118	YP_050593.1	-	hypothetical protein
ECA2502	2820114	2820354	YP_050594.1	-	hypothetical protein
ECA2503	2820770	2820968	YP_050595.1	-	putative phage-related protein
ECA2504	2821763	2822402	YP_050596.1	-	resolvase
ECA2505	2823148	2823316	YP_050597.1	-	hypothetical protein
ECA2506	2823640	2824555	YP_050598.1	-	hypothetical protein

PccI33
======

ECA2514	2830706	2831090	YP_050605.1	-	hypothetical protein
ECA2515	2831104	2831689	YP_050606.1	-	hypothetical protein

PccI34
======

ECA2606	2942680	2943172	YP_050697.1	-	putative bacteriophage tail fiber protein U
ECA2607	2943292	2943910	YP_050698.1	-	putative bacteriophage tail fiber assembly protein G
ECA2608	2943909	2945520	YP_050699.1	-	probable bacteriophage variable tail fiber protein H
ECA2609	2945516	2946122	YP_050700.1	-	putative phage tail protein I
ECA2610	2946114	2947011	YP_050701.1	-	phage baseplate assembly protein J
ECA2611	2946997	2947366	YP_050702.1	-	phage baseplate assembly protein W
ECA2612	2947362	2947944	YP_050703.1	-	baseplate assembly protein V
ECA2613	2947940	2948579	YP_050704.1	-	putative phage tail protein S
ECA2614	2948571	2949024	YP_050705.1	-	phage tail completion protein R
ECA2615	2949163	2949604	YP_050707.1	-	putative phage encoded host lysis, regulatory protein
ECA2616	2949600	2950143	YP_050708.1	-	hypothetical protein

PccI35
======

ECA2620	2951269	2952163	YP_050712.1	-	phage terminase, endonuclease small subunit M
ECA2621	2952214	2953264	YP_050713.1	-	major phage capsid protein N precursor
ECA2622	2953288	2954122	YP_050714.1	-	capsid scaffolding protein O
ECA2623	2954280	2956002	YP_050715.1	-	phage terminase, ATPase subunit P
ECA2624	2956003	2957050	YP_050716.1	-	phage capsid portal protein Q

ECA2625	2957425	2958115	YP_050717.1	-	phage DNA methylase
ECA2626	2958117	2958444	YP_050718.1	-	putative phage-related protein
ECA2627	2958536	2961047	YP_050719.1	-	putative phage replication protein A
ECA2628	2961046	2962075	YP_050720.1	-	conserved phage protein
ECA2629	2962067	2962916	YP_050721.1	-	phage DNA adenine methylase
ECA2630	2962912	2963812	YP_050722.1	-	hypothetical protein
ECA2631	2963808	2964042	YP_050723.1	-	hypothetical protein
ECA2632	2964112	2964448	YP_050724.1	-	hypothetical protein
ECA2633	2964528	2964654	YP_050725.1	-	hypothetical protein
ECA2634	2964650	2964851	YP_050726.1	-	phage-related hypothetical protein
ECA2635	2964847	2965210	YP_050727.1	-	phage regulatory protein
PccI36					
======					
ECA2693	3028797	3029253	YP_050784.1	-	hypothetical protein
ECA2694	3029318	3031103	YP_050785.1	-	putative polyketide synthetase
ECA2695	3031132	3032050	YP_050786.1	-	putative malonyl CoA-acyl carrier protein transacylase
ECA2696	3032046	3033456	YP_050787.1	ehpG	putative phenazine antibiotic biosynthesis protein
ECA2697	3033455	3034517	YP_050788.1	ehpF	putative phenazine antibiotic biosynthesis protein
ECA2698	3034529	3035168	YP_050789.1	ehpE	putative phenazine antibiotic biosynthesis protein
ECA2699	3035177	3036023	YP_050790.1	ehpD	putative phenazine antibiotic biosynthesis protein
ECA2700	3036001	3036352	YP_050791.1	-	hypothetical protein
ECA2701	3036348	3038226	YP_050792.1	ehpC	putative phenazine antibiotic biosynthesis protein
ECA2702	3038222	3038843	YP_050793.1	ehpB	putative phenazine antibiotic biosynthesis protein
ECA2703	3038922	3039381	YP_050794.1	ehpA	putative phenazine antibiotic biosynthesis protein
ECA2704	3039427	3040207	YP_050795.1	-	short chain dehydrogenase
ECA2705	3040364	3040751	YP_050796.1	ehpR	putative phenazine antibiotic biosynthesis protein
PccI37					
======					
ECA2754	3095609	3098216	YP_050845.1	-	putative prophage primase
ECA2755	3098461	3098917	YP_050846.1	-	hypothetical protein
ECA2756	3099004	3100030	YP_050847.1	-	hypothetical protein
PccI38					
======					
ECA2848	3192249	3193197	YP_050939.1	-	lLysR-family transcriptional regulator
ECA2849	3193308	3194337	YP_050940.1	-	metallo-beta-lactamase
ECA2850	3194781	3195210	YP_050941.1	mvpA	putative plasmid protein
ECA2851	3195209	3195440	YP_050942.1	mvpT	putative plasmid protein
ECA2852	3195533	3196367	YP_050943.1	-	hypothetical protein
ECA2853	3196484	3196805	YP_050944.1	-	hypothetical protein
ECA2854	3196874	3197252	YP_050945.1	-	hypothetical protein
PccI39					
======					
ECA2858	3199215	3199407	YP_050949.1	-	hypothetical protein
ECA2859	3199452	3199596	YP_050950.1	-	hypothetical protein
ECA2860	3199574	3200462	YP_050951.1	-	hypothetical protein
ECA2861	3200557	3201589	YP_050952.1	-	hypothetical protein
ECA2862	3202359	3202950	YP_050953.1	-	hypothetical protein
ECA2863	3203054	3203267	YP_050954.1	-	hypothetical protein
ECA2864	3203359	3203785	YP_050955.1	-	hypothetical protein
ECA2865	3203939	3204521	YP_050956.1	-	hypothetical protein
PccI40					
======					
ECA2874	3216323	3217214	YP_050965.1	-	hypothetical protein
ECA2875	3217206	3220539	YP_050966.1	-	putative ATP-binding protein
ECA2876	3220531	3221371	YP_050967.1	-	putative phage-related protein
ECA2877	3221521	3223375	YP_050968.1	-	hypothetical protein
ECA2878	3223377	3225771	YP_050969.1	-	putative signal transduction protein
PccI41					
======					
ECA2890	3237700	3238219	YP_050981.1	-	hypothetical protein
ECA2891	3238279	3239092	YP_050982.1	-	hypothetical protein
ECA2892	3239132	3239936	YP_050983.1	-	hypothetical protein
ECA2893	3241129	3241531	YP_050984.1	hns3	DNA-binding protein Hns
ECA2894	3241657	3242011	YP_050985.1	-	putative plasmid-related protein
ECA2895	3242010	3242289	YP_050986.1	-	hypothetical protein
ECA2896	3242491	3242674	YP_050987.1	-	hypothetical protein
ECA2897	3242707	3242992	YP_050988.1	-	hypothetical protein
ECA2898	3243721	3245125	YP_050989.1	-	putative plasmid mobilization protein
ECA2899	3245207	3246110	YP_050990.1	-	hypothetical protein
ECA2900	3246455	3247238	YP_050991.1	-	hypothetical protein
ECA2901	3248044	3248488	YP_050992.1	-	hypothetical protein
ECA2902	3248551	3248980	YP_050993.1	-	hypothetical protein
ECA2903	3249068	3249716	YP_050994.1	-	hypothetical protein
ECA2904	3249732	3250287	YP_050995.1	-	putative lipoprotein
ECA2905	3250921	3251299	YP_050996.1	-	hypothetical protein
ECA2906	3251566	3252769	YP_050997.1	-	hypothetical protein
ECA2907	3253050	3253389	YP_050998.1	-	hypothetical protein
ECA2908	3253485	3254250	YP_050999.1	-	putative plasmid replication protein
ECA2909	3255301	3255463	YP_051000.1	-	hypothetical protein
ECA2910	3255833	3256568	YP_051001.1	-	putative plasmid replication protein
ECA2911	3256753	3257866	YP_051002.1	-	putative DNA-binding protein
ECA2912	3257875	3258616	YP_051003.1	-	putative plasmid-related protein
ECA2913	3258797	3259049	YP_051004.1	-	putative DNA-binding protein

ECA2914	3259116	3259467	YP_051005.1	-	hypothetical protein
ECA2915	3259506	3259770	YP_051006.1	-	hypothetical protein
ECA2916	3259823	3260120	YP_051007.1	-	hypothetical protein
ECA2917	3260199	3260514	YP_051008.1	-	hypothetical protein
ECA2918	3260546	3260765	YP_051009.1	-	putative phage-related protein
ECA2919	3260914	3261778	YP_051010.1	-	hypothetical protein
ECA2920	3261770	3261941	YP_051011.1	-	hypothetical protein
ECA2921	3262079	3263492	YP_051012.1	-	hypothetical protein
ECA2922	3264766	3265660	YP_051013.1	-	LysR-family transcriptional regulator
ECA2923	3265815	3266967	YP_051014.1	-	putative NADH:flavin oxidoreductase
ECA2924	3267166	3267820	YP_051015.1	-	putative lipoprotein
Pccl42					
======					
ECA2933	3278434	3279220	YP_051024.1	-	probable short-chain dehydrogenase
ECA2934	3279352	3279733	YP_051025.1	-	putative transcriptional regulator
ECA2935	3279844	3280585	YP_051026.1	-	hypothetical protein
ECA2936	3280603	3281122	YP_051027.1	nifQ	nitrogen fixation protein
Pccl43					
======					
ECA2951	3296145	3297336	YP_051042.1	-	probable transporter
ECA2952	3297653	3298316	YP_051043.1	nifY	nitrogen fixation protein
ECA2953	3298329	3298548	YP_051044.1	nifT	nitrogen fixation protein
ECA2954	3298620	3300189	YP_051045.1	nifK	nitrogenase molybdenum-iron protein beta chain
ECA2955	3300247	3301696	YP_051046.1	nifD	nitrogenase molybdenum-iron protein alpha chain
ECA2956	3301756	3302638	YP_051047.1	nifH	nitrogenase iron protein
ECA2957	3303127	3306640	YP_051048.1	nifJ	pyruvate-flavodoxin oxidoreductase
Pccl44					
======					
ECA2972	3322878	3324231	YP_051063.1	-	putative hydrolase
ECA2973	3324256	3325147	YP_051064.1	-	LysR-family transcriptional regulator
ECA2974	3325374	3325824	YP_051065.1	-	MutT-like protein
ECA2975	3325953	3326604	YP_051066.1	-	hypothetical protein
ECA2976	3326721	3327630	YP_051067.1	-	LysR-family transcriptional regulator
ECA2977	3327795	3328329	YP_051068.1	-	hypothetical protein
ECA2978	3328333	3328660	YP_051069.1	-	hypothetical protein
ECA2979	3328673	3329513	YP_051070.1	-	probable hydrolase
ECA2980	3329724	3330060	YP_051071.1	-	hypothetical protein
ECA2981	3330104	3332162	YP_051072.1	-	hypothetical protein
ECA2982	3332181	3332514	YP_051073.1	emrE	multidrug efflux protein
Pccl45					
======					
ECA3401	3814022	3814433	YP_051492.1	-	putative phage regulatory protein
ECA3402	3814511	3814736	YP_051493.1	-	hypothetical protein
ECA3403	3814801	3815131	YP_051494.1	-	hypothetical protein
ECA3404	3815127	3815565	YP_051495.1	-	hypothetical protein
Pccl46					
======					
ECA3411	3821845	3822094	YP_051502.1	-	hypothetical protein
ECA3412	3822090	3822462	YP_051503.1	-	hypothetical protein
ECA3413	3822479	3822674	YP_051504.1	-	hypothetical protein
Pccl47					
======					
ECA3446	3865038	3866442	YP_051536.1	oprJ	multidrug resistance outer membrane protein
ECA3447	3866438	3869582	YP_051537.1	mexB	multidrug resistance protein
ECA3448	3869664	3870837	YP_051538.1	mexC	multidrug resistance protein
ECA3449	3871026	3871593	YP_051539.1	nfxB	transcriptional regulator of multidrug resistance genes
ECA3450	3871999	3872587	YP_051540.1	-	hypothetical protein
Pccl48					
======					
ECA3456	3875263	3876157	YP_051546.1	-	hypothetical protein
ECA3457	3876156	3876780	YP_051547.1	-	hypothetical protein
ECA3458	3876890	3877589	YP_051548.1	-	hypothetical protein
ECA3459	3877588	3878830	YP_051549.1	-	hypothetical protein
ECA3460	3878829	3880056	YP_051550.1	-	phage integrase
Pccl49					
======					
ECA3695	4144590	4144983	YP_051783.1	-	phage regulatory protein protein
ECA3696	4144979	4145402	YP_051784.1	-	putative phage regluatory protein
ECA3697	4145379	4145580	YP_051785.1	-	hypothetical protein
ECA3698	4145576	4146017	YP_051786.1	-	hypothetical protein
ECA3699	4146027	4146327	YP_051787.1	-	conserved hypothetical phage-related protein
ECA3700	4146417	4147026	YP_051788.1	-	conserved hypothetical phage-related protein
ECA3701	4147038	4147308	YP_051789.1	-	conserved hypothetical phage-related protein
ECA3702	4147335	4147593	YP_051790.1	-	hypothetical phage-related protein
ECA3703	4147595	4148747	YP_051791.1	-	conserved hypothetical phage-related protein
ECA3704	4148756	4150526	YP_051792.1	-	conserved hypothetical phage-related protein
ECA3705	4150535	4151444	YP_051793.1	-	conserved hypothetical phage-related protein
ECA3706	4151453	4151759	YP_051794.1	-	putative phage-related DNA-binding protein
ECA3707	4151811	4152000	YP_051795.1	-	putative phage-related DNA-binding protein
ECA3708	4152091	4152508	YP_051796.1	-	putative phage-related DNA-binding protein
ECA3709	4152526	4153063	YP_051797.1	-	putative phage-related membrane protein
ECA3710	4153097	4154042	YP_051798.1	-	putative phage-related lipoprotein
ECA3711	4154161	4155169	YP_051799.1	-	conserved hypothetical phage-related protein

PccI50
======

ECA3719	4159329	4160865	YP_051807.1	-	putative phage-related protein
ECA3720	4160864	4162358	YP_051808.1	-	putative phage-related protein
ECA3721	4162338	4163160	YP_051809.1	-	putative phage-related protein
ECA3722	4163156	4163606	YP_051810.1	-	putative phage-related protein
ECA3723	4163801	4164911	YP_051811.1	-	conserved phage-related protein
ECA3724	4164947	4165883	YP_051812.1	-	conserved phage-related protein

PccI51
======

ECA3731A	4169657	4169960	YP_051820.1	-	putative phage-related protein
ECA3732	4170346	4172818	YP_051821.1	-	putative bacteriophage tail protein (Tape-measure)
ECA3733	4172817	4173702	YP_051822.1	-	putative phage-related protein
ECA3734	4173698	4173914	YP_051823.1	-	putative bacteriophage tail fibre protein

PccI52
======

ECA4142	4624415	4626443	YP_052229.1	-	putative RHS accessory genetic element
ECA4143	4626444	4627170	YP_052230.1	-	hypothetical protein
ECA4144	4627166	4627685	YP_052231.1	-	hypothetical protein
ECA4145	4627806	4628325	YP_052232.1	-	hypothetical protein
ECA4146	4628324	4630040	YP_052233.1	-	hypothetical protein

PccI53
======

ECA4278	4792989	4797273	YP_052365.1	-	Rhs-family protein
ECA4279	4797273	4797585	YP_052366.1	-	hypothetical protein

PccI54
======

ECA4286	4803224	4803392	YP_052373.1	-	hypothetical protein
ECA4287	4803539	4803905	YP_052374.1	-	hypothetical protein
ECA4288	4803905	4804217	YP_052375.1	-	hypothetical protein

PccI55
======

ECA4381	4913049	4913964	YP_052466.1	-	putative DNA-binding protein
ECA4382	4914104	4915127	YP_052467.1	-	putative zinc-binding dehydrogenase

PccI56
======

ECA4388	4922194	4924099	YP_052473.1	-	PTS system, IIabc component
ECA4389	4924230	4925058	YP_052474.1	-	beta-glucoside operon antiterminator

PccI57
======

ECA4430	4969668	4970379	YP_052515.1	-	putative lipoprotein
ECA4431	4970378	4972373	YP_052516.1	-	hypothetical protein

PccI58
======

ECA4452	4993334	4994663	YP_052537.1	hmsR	N-glycosyltransferase PgaC
ECA4453	4994659	4996675	YP_052538.1	hmsF	putative hemin storage lipoprotein
ECA4454	4996686	4999149	YP_052539.1	hmsH	predicted outer membrane protein
ECA4455	4999352	5000237	YP_052540.1	-	hypothetical protein

PccI59
======

ECA4481	5024024	5024777	YP_052564.1	-	probable short-chain dehydrogenase
ECA4482	5024879	5025287	YP_052565.1	-	hypothetical protein
ECA4483	5025429	5026353	YP_052566.1	nac	nitrogen assimilation transcriptional regulator
ECA4484	5026475	5026682	YP_052567.1	-	hypothetical protein
ECA4485	5026699	5027902	YP_052568.1	aatA	aspartate aminotransferase
ECA4486	5027971	5028745	YP_052569.1	-	hypothetical protein
ECA4487	5028744	5029737	YP_052570.1	-	putative allophanate hydrolase subunit 2
ECA4488	5029733	5030435	YP_052571.1	-	putative allophanate hydrolase subunit 1
ECA4489	5030438	5031278	YP_052572.1	-	putative extracellular solute-binding protein
ECA4490	5031539	5032457	YP_052573.1	-	hypothetical protein
ECA4491	5032591	5033818	YP_052574.1	-	3-oxoacyl-(acyl carrier protein) synthase II
ECA4492	5033817	5034549	YP_052575.1	fabG	3-ketoacyl-(acyl-carrier-protein) reductase
ECA4493	5034587	5035070	YP_052576.1	-	putative beta-hydroxydecanoyl-ACP dehydrase

PccI60
======

ECA4501	5042192	5043548	YP_052584.1	-	putative AMP-binding enzyme
ECA4502	5043544	5044123	YP_052585.1	-	hypothetical protein
ECA4503	5044135	5044387	YP_052586.1	-	acyl carrier protein
ECA4504	5044404	5044671	YP_052587.1	-	putative acyl carrier protein
