# Supplementary material for: Microarray Comparative Genomic Hybridisation Analysis Incorporating Genomic Organisation, and Application to Enterobacterial Plant Pathogens
Source: PLoS Comput Biol. 2009 Aug 21;5(8):e1000473. doi: 10.1371/journal.pcbi.1000473 (PMC2718846; doi:10.1371/journal.pcbi.1000473)
Supplement: Table S4 — 165 Pba1043 genomic islands predicted to have no orthologues in Dda3937, prefixed DdaI. (0.23 MB PDF) [file pcbi.1000473.s004.pdf]

Supplementary Table 4: 165 *Pba*1043 genomic islands predicted to have no orthologues in *Dda*3937, prefixed DdaI.

DdaI1
=====

ECA0043	51399	53091	YP_048172.1	ligB	NAD-dependent DNA ligase LigB
ECA0044	53349	55035	YP_048173.1	-	phosphoethanolamine transferase
ECA0045	55764	56313	YP_048174.1	-	putative virulence-associated outer membrane protein

DdaI2
=====

ECA0063	79097	79385	YP_048192.1	-	hypothetical protein
ECA0064	79381	79633	YP_048193.1	-	hypothetical protein
ECA0065	79888	80794	YP_048194.1	-	hypothetical protein
ECA0066	80828	81584	YP_048195.1	dsbG	disulfide isomerase/thiol-disulfide oxidase
ECA0067	81804	82428	YP_048196.1	-	putative LysE family transporter
ECA0068	82474	83347	YP_048197.1	-	LysR-family transcriptional regulator

DdaI3
=====

ECA0077	91665	92868	YP_048205.1	-	hypothetical protein
ECA0078	92959	93922	YP_048206.2	tkrA	2-hydroxyacid dehydrogenase
ECA0079	93966	94173	YP_048207.1	-	hypothetical protein
ECA0080	94275	96228	YP_048208.1	-	methyl-accepting chemotaxis protein

DdaI4
=====

ECA0105	125688	126339	YP_048233.1	expI	N-acylhomoserine lactone synthesis protein
ECA0106	126322	127051	YP_048234.1	expR	quorum-sensing transcriptional regulator

DdaI5
=====

ECA0115	134971	136117	YP_048243.1	-	putative fatty acid desaturase
ECA0116	136116	137208	YP_048244.1	-	putative fatty acid desaturase

DdaI6
=====

ECA0120	141448	142765	YP_048248.1	-	hypothetical protein
ECA0121	142829	143192	YP_048249.1	-	hypothetical protein
ECA0122	143223	143610	YP_048250.1	-	hypothetical protein
ECA0123	143718	144270	YP_048251.1	-	putative lipoprotein
ECA0124	144321	144879	YP_048252.1	-	putative lipoprotein
ECA0125	144969	145197	YP_048253.1	-	hypothetical protein
ECA0126	145495	145804	YP_048254.1	-	hypothetical protein
ECA0127	145800	147051	YP_048255.1	-	hypothetical protein
ECA0128	147193	147496	YP_048256.1	-	hypothetical protein
ECA0129	148810	150391	YP_048257.1	-	putative deoxycytidylate deaminase

DdaI7
=====

ECA0149	169391	170366	YP_048277.1	-	putative sugar transferase
ECA0150	170374	170854	YP_048278.1	coaD	phosphopantetheine adenylyltransferase
ECA0151	170890	172168	YP_048279.1	kdtA	3-deoxy-D-manno-octulosonic-acid transferase
ECA0152	172395	173259	YP_048280.1	-	putative glycosyltransferase
ECA0153	173306	174074	YP_048281.1	kdtX	lipopolysaccharide core biosynthesis glycosyl transferase
ECA0154	174160	174907	YP_048282.1	waaX	putative beta1,4-galactosyltransferase
ECA0155	174977	175163	YP_048283.1	-	hypothetical protein
ECA0156	175282	176065	YP_048284.1	-	hypothetical protein
ECA0157	176213	177230	YP_048285.1	waaJ	lipopolysaccharide 1,2-glucosyltransferase
ECA0158	177283	178294	YP_048286.1	waaI	lipopolysaccharide 1,3-galactosyltransferase
ECA0159	178360	179479	YP_048287.1	waaG	lipopolysaccharide core biosynthesis protein
ECA0160	179475	180546	YP_048288.1	waaQ	lipopolysaccharide core biosynthesis protein
ECA0161	180542	181769	YP_048289.1	waaL2	O-antigen ligase
ECA0162	182260	183544	YP_048290.1	waaL1	O-antigen ligase
ECA0163	183540	184524	YP_048291.1	waaC	ADP-heptose:LPS heptosyl transferase I

DdaI8
=====

ECA0176	196153	196633	YP_048303.1	-	hypothetical protein
ECA0177	196649	197366	YP_048304.1	-	hypothetical protein
ECA0178	197366	197699	YP_048305.1	-	hypothetical protein

DdaI9
=====

ECA0182	202589	204218	YP_048309.1	-	methyl-accepting chemotaxis protein
ECA0183	204905	206567	YP_048310.1	-	methyl-accepting chemotaxis protein

DdaI10
======

ECA0189	214456	215656	YP_048316.1	-	putative mandelate racemase / muconate lactonizing enzyme
ECA0190	215661	216387	YP_048317.1	-	GntR-family transcriptional regulator

DdaI11
======

ECA0225	264270	265668	YP_048352.1	shiA	shikimate transporter
ECA0226	265738	265930	YP_048353.1	-	hypothetical protein
ECA0227	266103	267540	YP_048354.1	-	putative GntR-family transcriptional regulator

DdaI12
======

ECA0249	294648	295059	YP_048376.1	-	hypothetical protein
ECA0250	295275	296334	YP_048377.1	-	putative oxidoreductase
ECA0251	296588	297767	YP_048378.1	-	endonuclease/Exonuclease/phosphatase family protein

ECA0252	297835	298240	YP_048379.1	-	hypothetical protein
ECA0253	298565	299036	YP_048380.1	doc	putative death on curing protein
ECA0254	299035	299440	YP_048381.1	-	hypothetical protein
DdaI13					
======					
ECA0338	386070	386934	YP_048464.1	-	hypothetical protein
ECA0339	387023	387875	YP_048465.1	-	probable sugar-bisphosphate aldolase
ECA0340	387886	388978	YP_048466.1	-	PTS system, IIbc component
ECA0341	389003	389318	YP_048467.1	-	PTS system, IIb component
ECA0342	389331	389811	YP_048468.1	-	PTS system, EIIa component
ECA0343	389859	391410	YP_048469.1	-	probable PTS system regulatory protein
ECA0344	391921	392257	YP_048470.1	-	hypothetical protein
DdaI14					
======					
ECA0365	417454	418135	YP_048491.1	-	hypothetical protein
ECA0366	418294	418606	YP_048492.1	-	hypothetical protein
ECA0367	418602	419019	YP_048493.1	-	putative DNA-binding protein
DdaI15					
======					
ECA0394	451995	452394	YP_048518.1	-	hypothetical protein
ECA0395	452394	452937	YP_048519.1	kptA	RNA 2'-phosphotransferase
ECA0396	453260	454961	YP_048520.1	-	putative haemolysin-like protein
ECA0397	454962	455337	YP_048521.1	-	hypothetical protein
ECA0398	455618	456002	YP_048522.1	-	hypothetical protein
ECA0399	456047	456269	YP_048523.1	-	hypothetical protein
ECA0399A	456304	456442	YP_048524.1	-	putative hemagglutinin-like protein (partial)
ECA0400	456425	456719	YP_048525.1	-	putative phage-related protein
ECA0401	457210	457861	YP_048526.1	-	hypothetical protein
DdaI16					
======					
ECA0407	466209	466377	YP_048532.1	-	putative integrase (partial)
ECA0408	466557	466815	YP_048533.1	-	putative plasmid-related protein
ECA0410	467141	467654	YP_048535.1	-	putative kinase
ECA0413	469395	472884	YP_048536.1	-	hypothetical protein
ECA0414	473424	473673	YP_048537.1	-	putative bacteriophage regulatory protein
DdaI17					
======					
ECA0423	479800	480919	YP_048541.1	-	hypothetical protein
ECA0424	480918	481917	YP_048542.1	-	hypothetical protein
ECA0425	481913	483335	YP_048543.1	-	putative outer membrane efflux protein
ECA0426	483704	485144	YP_048544.1	aspA1	aspartate ammonia-lyase
ECA0427	485397	486354	YP_048545.1	-	LysR-family transcriptional regulator
ECA0428	487121	488642	YP_048546.1	-	putative porin
ECA0429	488658	490308	YP_048547.1	-	hypothetical protein
ECA0430	490304	491135	YP_048548.1	-	hypothetical protein
ECA0431	491594	492941	YP_048549.1	-	hypothetical protein
ECA0432	492952	493744	YP_048550.1	-	hypothetical protein
ECA0433	493896	494994	YP_048551.1	-	putative lipoprotein
ECA0434	495148	497098	YP_048552.1	-	methyl-accepting chemotaxis protein
ECA0435	497560	500272	YP_048553.1	mgtB	Magnesium transport ATPase
ECA0436	500718	502296	YP_048554.1	-	methyl-accepting chemotaxis protein
DdaI18					
======					
ECA0443	508847	509102	YP_048561.1	-	hypothetical protein
ECA0444	509098	509350	YP_048562.1	-	antitoxin YefM
DdaI19					
======					
ECA0452	518033	519071	YP_048570.1	-	putative zinc-binding dehydrogenase
ECA0454	519484	519781	YP_048572.1	-	hypothetical protein
ECA0455	519799	520555	YP_048573.1	-	hypothetical protein
ECA0456	520561	521041	YP_048574.1	-	hypothetical protein
DdaI20					
======					
ECA0467	532187	533090	YP_048585.1	-	hypothetical protein
ECA0468	533381	534971	YP_048586.1	prfC	peptide chain release factor 3
ECA0469	535472	536081	YP_048587.1	osmY	periplasmic protein
DdaI21					
======					
ECA0473	537937	539215	YP_048591.1	-	putative Na+ dependent nucleoside transporter-family protein
ECA0474	539364	540081	YP_048592.1	entD	enterobactin synthetase component D (4'-phosphopantetheinyl transferase)
ECA0475	540115	542077	YP_048593.1	-	TonB-dependent siderophore receptor protein
DdaI22					
======					
ECA0485	560282	562364	YP_048603.1	-	hypothetical protein
ECA0486	562386	562722	YP_048604.1	-	hypothetical protein
ECA0487	563151	564084	YP_048605.1	fom1	phosphoenolpyruvate phosphomutase
ECA0488	564263	565418	YP_048606.1	fom2	phosphonopyruvate decarboxylase
ECA0489	565470	566364	YP_048607.1	-	putative 2-hydroxy-3-oxopropionate reductase
ECA0490	566364	566808	YP_048608.1	phnG	putative phosphonate metabolism protein
ECA0491	566823	567396	YP_048609.1	phnH	carbon-phosphorus lyase complex subunit
ECA0492	567395	568499	YP_048610.1	phnI	putative phosphonate metabolism protein
DdaI23					

======					
ECA0497	572076	572643	YP_048615.1	phnN	ribose 1,5-bisphosphokinase
ECA0498	572684	573470	YP_048616.1	phnP	carbon-phosphorus lyase complex accessory protein
DdaI24					
======					
ECA0516	590843	591731	YP_048634.1	-	hypothetical protein
ECA0517	591723	592413	YP_048635.1	-	hypothetical protein
ECA0518	592409	592802	YP_048636.1	-	hypothetical protein
ECA0519	592767	594162	YP_048637.1	-	replicative DNA helicase
ECA0520	594161	595886	YP_048638.1	-	hypothetical protein
ECA0521	595889	596162	YP_048639.1	-	hypothetical protein
ECA0522	596186	596801	YP_048640.1	-	hypothetical protein
ECA0522A	596809	597064	YP_048641.1	-	hypothetical protein
ECA0523	597236	598433	YP_048642.1	-	hypothetical protein
ECA0524	598802	599600	YP_048643.1	-	hypothetical protein
ECA0525	599596	601615	YP_048644.1	topB	DNA topoisomerase III
ECA0526	601918	602095	YP_048645.1	-	hypothetical protein
ECA0527	602137	602293	YP_048646.1	-	hypothetical protein
ECA0528	602463	602955	YP_048647.1	-	hypothetical protein
ECA0531	603653	604091	YP_048648.1	-	putative plasmid-related protein
ECA0532	604236	605553	YP_048649.1	pilL	putative Type IV pilus protein
ECA0533	605552	605999	YP_048650.1	pilM	putative Type IV pilus protein
ECA0534	606012	607677	YP_048651.1	pilN	putative Type IV pilus protein
ECA0537	608989	609487	YP_048652.1	pilP	putative Type IV pilus protein
ECA0538	609486	611046	YP_048653.1	pilQ	putative Type IV pilus nucleotide-binding protein
ECA0539	611014	611167	YP_048654.1	-	hypothetical protein
ECA0540	611177	612215	YP_048655.1	pilR	putative Type IV pilus protein
ECA0541	612270	612876	YP_048656.1	pilS	putative type IV pilus prepilin
ECA0542	612882	613401	YP_048657.1	pilT	putative Type IV pilus protein
ECA0543	613397	614057	YP_048658.1	pilU	putative prepilin peptidase
ECA0544	614070	615465	YP_048659.1	pilV	putative Type IV pilus prepilin protein
ECA0545	615468	615948	YP_048660.1	-	alternative C-terminus for the PilV protein (fragment)
ECA0546	616234	617362	YP_048662.1	rci	shufflon-specific DNA recombinase
ECA0547	617410	617902	YP_048663.1	-	hypothetical protein
ECA0548	618089	618890	YP_048664.1	traE	putative plasmid transfer protein
ECA0549	619033	620236	YP_048665.1	traF	putative plasmid transfer protein
ECA0550	620302	620752	YP_048666.1	-	putative plasmid protein protein
ECA0551	620818	621118	YP_048667.1	-	hypothetical protein
ECA0552	621155	621848	YP_048668.1	-	hypothetical protein
ECA0553	621945	622320	YP_048669.1	-	hypothetical protein
ECA0554	622422	623298	YP_048670.1	-	hypothetical protein
ECA0555	623307	624021	YP_048671.1	-	hypothetical protein
ECA0556	624020	624608	YP_048672.1	-	hypothetical protein
ECA0557	624608	625130	YP_048673.1	-	hypothetical protein
ECA0558	625126	625744	YP_048674.1	-	hypothetical protein
ECA0559	625721	626204	YP_048675.1	-	hypothetical protein
ECA0560	626207	628304	YP_048676.1	-	putative plasmid transfer protein
ECA0561	628305	629061	YP_048677.1	-	hypothetical protein
ECA0562	629083	629278	YP_048678.1	-	hypothetical protein
ECA0563	629419	629959	YP_048679.1	-	hypothetical protein
ECA0564	630093	630414	YP_048680.1	-	hypothetical protein
ECA0565	630415	630655	YP_048681.1	-	hypothetical protein
ECA0566	630681	631023	YP_048682.1	-	hypothetical protein
ECA0567	631032	631395	YP_048683.1	-	hypothetical protein
ECA0568	631391	632048	YP_048684.1	-	hypothetical protein
ECA0569	632044	632971	YP_048685.1	-	hypothetical protein
ECA0570	632960	634493	YP_048686.1	-	hypothetical protein
ECA0571	634610	634946	YP_048687.1	-	hypothetical protein
ECA0572	634938	635349	YP_048688.1	-	putative lipoprotein
ECA0573	635348	638210	YP_048689.1	-	putative plasmid-related protein
ECA0574	638206	638653	YP_048690.1	-	hypothetical protein
ECA0575	638754	639189	YP_048691.1	-	hypothetical protein
ECA0576	639328	639733	YP_048692.1	-	hypothetical protein
ECA0577	639725	640700	YP_048693.1	-	hypothetical protein
ECA0578	640709	642134	YP_048694.1	-	hypothetical protein
ECA0579	642136	642508	YP_048695.1	-	hypothetical protein
ECA0580	642504	644037	YP_048696.1	-	hypothetical protein
ECA0581	644069	644459	YP_048697.1	-	hypothetical protein
ECA0582	644601	644961	YP_048698.1	-	putative plasmid-related protein
ECA0583	644957	645260	YP_048699.1	-	putative plasmid-related protein
ECA0584	645557	647429	YP_048700.1	-	restriction enzyme alpha subunit
ECA0585	647418	648405	YP_048701.1	-	restriction enzyme beta subunit
ECA0586	648537	648678	YP_048702.1	-	hypothetical protein
ECA0586A	648730	648898	YP_048703.1	-	hypothetical protein
ECA0587	649513	650404	YP_048704.1	-	hypothetical protein
ECA0588	650411	650702	YP_048705.1	-	putative plasmid-related protein
ECA0589	651096	651408	YP_048706.1	-	hypothetical protein
ECA0590	651457	651814	YP_048707.1	-	hypothetical protein
ECA0591	651895	652369	YP_048708.1	-	hypothetical protein
ECA0592	652457	652862	YP_048709.1	-	hypothetical protein
ECA0593	653072	653486	YP_048710.1	-	putative DNA repair protein
ECA0594	653662	654259	YP_048711.1	-	hypothetical protein
ECA0595	654255	654528	YP_048712.1	-	hypothetical protein

ECA0596	654598	655084	YP_048713.1	-	hypothetical protein
ECA0597	655149	657111	YP_048714.1	-	hypothetical protein
ECA0598	657185	658079	YP_048715.1	-	hypothetical protein
ECA0599	658153	659074	YP_048716.1	-	hypothetical protein
ECA0600	659712	661026	YP_048717.1	cfa8B	putative oxidoreductase
ECA0601	661109	661541	YP_048718.1	cfa8A	putative oxidoreductase
ECA0602	661604	667991	YP_048719.1	cfa7	type I polyketide synthase
ECA0603	667987	676129	YP_048720.1	cfa6	type I polyketide synthase
ECA0604	676142	677582	YP_048721.1	cfa5	coronafacic acid synthetase, ligase component
ECA0605	677578	678112	YP_048722.1	cfa4	coronafacic acid synthetase component
ECA0606	678108	679254	YP_048723.1	cfa3	Cfa-beta-ketoacylsynthase
ECA0607	679247	679745	YP_048724.1	cfa2	coronafacic acid dehydratase
ECA0608	679741	680017	YP_048725.1	cfa1	Cfa-acyl carrier protein
ECA0609	680118	681681	YP_048726.1	cfl	coronafacate ligase
ECA0610	681940	682903	YP_048727.1	-	LysR-family transcriptional regulator
ECA0611	683331	684792	YP_048728.1	-	hypothetical protein
ECA0612	684911	685403	YP_048729.1	-	hypothetical protein
ECA0613	685695	687294	YP_048730.1	-	hypothetical protein
ECA0614	687379	688402	YP_048731.1	-	putative phage integrase
ECA0615	688831	689677	YP_048732.1	-	AraC-family transcriptional regulator
DdaI25					
======					
ECA0640	710755	711145	YP_048756.1	-	hypothetical protein
ECA0641	711150	711546	YP_048757.1	-	hypothetical protein
DdaI26					
======					
ECA0658	731958	732447	YP_048773.1	-	periplasmic protein
ECA0659	732645	734115	YP_048774.1	-	putative signaling membrane protein
DdaI27					
======					
ECA0665	739282	740689	YP_048780.1	-	putative phage integrase
ECA0666	740666	741473	YP_048781.1	-	hypothetical protein
ECA0667	741624	741816	YP_048782.1	-	hypothetical protein
ECA0668	741881	742790	YP_048783.1	-	hypothetical protein
ECA0669	742779	743127	YP_048784.1	-	hypothetical protein
ECA0670	743119	743425	YP_048785.1	-	hypothetical protein
ECA0671	743497	743797	YP_048786.1	-	putative bacteriophage derepression protein
ECA0672	743860	746539	YP_048787.1	-	putative phage-related protein
ECA0673	747114	747489	YP_048788.1	-	putative phage-related DNA binding protein
ECA0674	747552	747822	YP_048789.1	-	putative phage-related protein
ECA0675	748134	748305	YP_048790.1	-	hypothetical protein
DdaI28					
======					
ECA0705	774295	774733	YP_048819.1	-	putative degenerate non-ribosomal peptide synthetase (partial)
ECA0706	774825	775116	YP_048820.1	-	putative degenerate non-ribosomal peptide synthetase (partial)
ECA0707A	775622	775718	YP_048821.1	-	hypothetical protein (partial)
ECA0708	776321	776672	YP_048822.1	evr	virulence regulator
DdaI29					
======					
ECA0731	799577	801830	YP_048843.1	fcuA	putative ferric siderophore TonB-dependent receptor
ECA0732	801919	802399	YP_048844.1	-	hypothetical protein
DdaI30					
======					
ECA0762	834357	834681	YP_048874.1	-	putative lipoprotein
ECA0763	834709	835228	YP_048875.1	fldB	flavodoxin FldB
ECA0764	835455	835632	YP_048876.1	-	hypothetical protein
ECA0765	835646	836093	YP_048877.1	-	hypothetical protein
ECA0766	836293	836902	YP_048878.1	-	hypothetical protein
ECA0767	837168	837669	YP_048879.1	-	hypothetical protein
ECA0768	837919	838096	YP_048880.1	-	hypothetical protein
DdaI31					
======					
ECA0777	848522	849212	YP_048889.1	-	hypothetical protein
ECA0778	849287	851009	YP_048890.1	-	hypothetical protein
ECA0779	851059	851452	YP_048891.1	-	hypothetical protein
ECA0780	851562	852633	YP_048892.1	-	hypothetical protein
DdaI32					
======					
ECA0785	858629	859340	YP_048897.1	-	two component system response regulator
ECA0786	859317	860817	YP_048898.1	-	two component system sensor kinase
ECA0787	861359	861911	YP_048899.1	-	hypothetical protein
ECA0788	861959	862904	YP_048900.1	-	hypothetical protein
DdaI33					
======					
ECA0797	871251	871884	YP_048909.1	-	hypothetical protein
ECA0798	872063	873695	YP_048910.1	-	hypothetical protein
ECA0799	873753	883440	YP_048911.1	-	putative outer membrane protein
DdaI34					
======					
ECA0804	890039	891770	YP_048916.1	rhiE	rhamnogalacturonate lyase
ECA0805	891898	892831	YP_048917.1	-	hypothetical protein
DdaI35					
======					

ECA0818	907055	907505	YP_048928.1	-	hypothetical protein
ECA0819	907497	908973	YP_048929.1	uxuB	putative D-mannonate oxidoreductase
ECA0820	909307	910588	YP_048930.1	-	O-acetyl-L-homoserine sulfhydrylase
ECA0821	910650	911748	YP_048931.1	-	hypothetical protein
ECA0822	911923	912721	YP_048932.1	-	putative carbon-nitrogen hydrolase
ECA0823	912742	913279	YP_048933.1	cybB	cytochrome B561
ECA0824	913552	917086	YP_048934.1	-	pyruvate-flavodoxin oxidoreductase
ECA0825	917475	917901	YP_048935.1	-	hypothetical protein
ECA0826	918245	918758	YP_048936.1	-	putative GNAT-family acetyltransferase
ECA0827	918801	919428	YP_048937.1	-	putative amino acid efflux protein
ECA0828	919624	920407	YP_048938.1	-	putative amino acid-binding periplasmic protein
ECA0829	920664	921243	YP_048939.1	-	hypothetical protein
ECA0830	921447	921897	YP_048940.1	-	hypothetical protein
ECA0831	921905	922199	YP_048941.1	-	putative transposase (partial)
ECA0832	922239	922566	YP_048942.1	-	putative phage capsid protein (partial)
ECA0833	922584	922755	YP_048943.1	-	phage regulatory protein
ECA0834	923067	925512	YP_048944.1	-	hypothetical protein
ECA0835	925540	926821	YP_048945.1	-	prophage integrase
DdaI36					
======					
ECA0849	939858	940740	YP_048959.1	-	putative sugar ABC transporter, permease protein
ECA0850	940741	941554	YP_048960.1	-	putative sugar ABC transporter, permease protein
ECA0851	941590	942676	YP_048961.1	-	putative sugar ABC transporter ATP-binding protein
ECA0852	943024	944548	YP_048962.1	-	putative exported plant proteoglycan hydrolase
DdaI37					
======					
ECA0856	947608	948505	YP_048966.1	-	ABC transporter, periplasmic binding protein
ECA0858	948917	949769	YP_048967.1	-	putative beta-glucoside operon antiterminator
DdaI38					
======					
ECA0863	956802	957261	YP_048972.1	rpiB	ribose-5-phosphate isomerase B
ECA0864	957604	957808	YP_048973.1	-	hypothetical protein
DdaI39					
======					
ECA0876	971014	973210	YP_048985.1	iutA	TonB-dependent ferric aerobactin receptor
ECA0877	973974	974826	YP_048986.1	-	TonB-like protein
ECA0878	974923	977506	YP_048987.1	-	hypothetical protein
ECA0879	977611	980386	YP_048988.1	-	putative zinc protease
ECA0880	980391	982056	YP_048989.1	-	putative ABC transporter ATP-binding protein
DdaI40					
======					
ECA0921	1029049	1029736	YP_049030.1	-	GntR-family transcriptional regulator
ECA0922	1029896	1030829	YP_049031.1	-	LysR-family transcriptional regulator
ECA0923	1030958	1031849	YP_049032.1	-	hypothetical protein
DdaI41					
======					
ECA0935	1046526	1048740	YP_049044.1	fhuE	ferric-rhodotorulic acid outer membrane transporter
ECA0936	1048832	1050182	YP_049045.1	-	putative glucarate dehydratase
ECA0937	1050174	1051521	YP_049046.1	-	putative transport protein
ECA0938	1051811	1052303	YP_049047.1	-	hypothetical protein
ECA0939	1052369	1052939	YP_049048.1	-	putative fimbrial protein
ECA0940	1052966	1054001	YP_049049.1	-	hypothetical protein
ECA0941	1054019	1054769	YP_049050.1	-	putative fimbrial chaperone
ECA0942	1054807	1057300	YP_049051.1	-	putative outer membrane usher protein
ECA0943	1057441	1057990	YP_049052.1	-	fimbriae major subunit protein
ECA0944	1058635	1059439	YP_049053.1	-	hypothetical protein
ECA0945	1059425	1059896	YP_049054.1	-	hypothetical protein
ECA0946	1059957	1060530	YP_049055.1	-	TetR-family transcriptional regulator
ECA0947	1060622	1061516	YP_049056.1	-	hypothetical protein
ECA0948	1061621	1062176	YP_049057.1	-	hypothetical protein
ECA0949	1062409	1063174	YP_049058.1	-	hypothetical protein
ECA0950	1063489	1064089	YP_049059.1	-	TetR-family transcriptional regulator
ECA0951	1064206	1064794	YP_049060.1	-	probable NAD(P)H oxidoreductase
ECA0952	1064819	1064996	YP_049061.1	-	hypothetical protein
ECA0953	1065059	1065287	YP_049062.1	-	hypothetical protein
DdaI42					
======					
ECA0970	1080116	1081325	YP_049077.1	-	hypothetical protein
ECA0971	1081338	1082172	YP_049078.1	-	hypothetical protein
ECA0972	1082386	1083430	YP_049079.1	-	hypothetical protein
ECA0973	1083392	1084556	YP_049080.1	-	AraC-family transcriptional regulator
DdaI43					
======					
ECA1002	1122905	1123823	YP_049109.1	-	putative dihydrodipicolinate synthase
ECA1003	1123896	1124589	YP_049110.1	-	putative tetR-family transcription factor
DdaI44					
======					
ECA1052	1177367	1178066	YP_049159.1	cutF	lipoprotein involved with copper homeostasis and adhesion
ECA1053	1178138	1180703	YP_049160.1	mutS	DNA mismatch repair protein
ECA1054	1180861	1182331	YP_049161.1	-	putative integrase
ECA1055	1182390	1182576	YP_049162.1	-	hypothetical protein
ECA1056	1182639	1183911	YP_049163.1	-	putative phage-related reverse transcriptase/maturase family protein

ECA1057	1183907	1185917	YP_049164.1	-	phage-related hypothetical protein
ECA1058	1186180	1186795	YP_049165.1	-	hypothetical protein
ECA1059	1187196	1188408	YP_049166.1	-	putative integrase
ECA1060	1188400	1189894	YP_049167.1	-	putative integrase
ECA1061	1189893	1191858	YP_049168.1	-	putative integrase
ECA1062	1191838	1192258	YP_049169.1	-	hypothetical protein
ECA1063	1192338	1192806	YP_049170.1	-	hypothetical protein
ECA1064	1192860	1193283	YP_049171.1	-	hypothetical protein
ECA1065	1193408	1194704	YP_049172.1	-	hypothetical protein
ECA1066	1194709	1195108	YP_049173.1	-	hypothetical protein
ECA1067	1195300	1196644	YP_049174.1	-	hypothetical protein
ECA1068	1197375	1198215	YP_049175.1	-	hypothetical protein
ECA1069	1198649	1199129	YP_049176.1	-	hypothetical protein
ECA1070	1199329	1200754	YP_049177.1	-	putative 3-polyprenyl-4-hydroxybenzoate carboxy-lyase
DdaI45					
======					
ECA1079	1209125	1209647	YP_049186.1	fecI	RNA polymerase sigma factor FecI
ECA1080	1209692	1209875	YP_049187.1	-	hypothetical protein
ECA1081	1209939	1211562	YP_049188.1	-	probable transcriptional regulator
DdaI46					
======					
ECA1089	1217226	1218471	YP_049196.1	-	hypothetical protein
ECA1090	1218825	1220019	YP_049197.1	-	putative lipoprotein
ECA1092	1220230	1220965	YP_049198.1	-	GntR-family transcriptional regulator
ECA1093	1221232	1222429	YP_049199.1	uxuA	mannonate dehydratase
ECA1094	1222650	1223694	YP_049200.1	pelI	pectate lyase
ECA1095	1224543	1225752	YP_049201.1	pehA	endo-polygalacturonase
ECA1096	1225994	1227164	YP_049202.1	-	putative type I secretion protein
ECA1097	1227175	1229320	YP_049203.1	-	putative type I secretion protein, ATP-binding protein
ECA1098	1229353	1230796	YP_049204.1	-	putative type I secretion protein
ECA1099	1231300	1242148	YP_049205.1	-	large repetitive protein
ECA1100	1242165	1245936	YP_049206.1	-	hypothetical protein
ECA1101	1246645	1248292	YP_049207.1	-	multidrug transporter membrane component/ATP-binding component
ECA1102	1248507	1249548	YP_049208.1	ansB1	L-asparaginase precursor
ECA1103	1250101	1250623	YP_049209.1	aroL	shikimate kinase II
ECA1104	1250761	1251046	YP_049210.1	-	hypothetical protein
ECA1105	1251538	1253329	YP_049211.1	-	putative methyl-accepting chemotaxis protein
ECA1106	1253390	1254320	YP_049212.1	rdgC	recombination associated protein
ECA1107	1254671	1255583	YP_049213.1	-	fructokinase
DdaI47					
======					
ECA1183	1339633	1339852	YP_049289.1	-	hypothetical protein
ECA1184	1339958	1341608	YP_049290.1	ushA	bifunctional UDP-sugar hydrolase/5'-nucleotidase periplasmic precursor
ECA1185	1341796	1343851	YP_049291.1	-	hypothetical protein
ECA1186	1343980	1346041	YP_049292.1	-	hypothetical protein
DdaI48					
======					
ECA1191	1351948	1352428	YP_049297.1	-	hypothetical protein
ECA1192	1352498	1353317	YP_049298.1	-	hypothetical protein
DdaI49					
======					
ECA1204	1369799	1372484	YP_049310.1	-	phosphotransfer intermediate protein in two-component regulatory system with RcsBC
ECA1205	1372497	1373667	YP_049311.1	-	hypothetical protein
ECA1206	1373803	1374223	YP_049312.1	-	probable transcriptional regulator
ECA1207	1374600	1375371	YP_049313.1	-	probable short chain dehydrogenase
ECA1208	1375436	1376318	YP_049314.1	-	probable short chain dehydrogenase
DdaI50					
======					
ECA1248	1419552	1419789	YP_049354.1	-	hypothetical protein
ECA1249	1419999	1420545	YP_049355.1	hydN	electron transport protein HydN
ECA1250	1420601	1422752	YP_049356.1	fdhF	formate dehydrogenase H
ECA1251	1422748	1425052	YP_049357.1	hypF	hydrogenase maturation protein
DdaI51					
======					
ECA1274	1448116	1448638	YP_049380.1	-	RNA polymerase sigma factor
ECA1275	1449047	1451423	YP_049381.1	-	TonB-dependent ferrichrome-iron receptor
ECA1276	1451497	1453288	YP_049382.1	-	ABC transporter ATP binding component
ECA1277	1453349	1453721	YP_049383.1	-	hypothetical protein
DdaI52					
======					
ECA1281	1455487	1457158	YP_049387.1	-	methyl-accepting chemotaxis protein
ECA1282	1457256	1458435	YP_049388.1	atoB	acetyl-CoA acetyltransferase
ECA1283	1458446	1459094	YP_049389.1	atoA	acetate CoA-transferase beta subunit
ECA1284	1459093	1459750	YP_049390.1	atoD	acetyl-CoA:acetoacetyl-CoA transferase subunit alpha
ECA1285	1459906	1460206	YP_049391.1	-	hypothetical protein
ECA1286	1460205	1461546	YP_049392.1	-	hypothetical protein
ECA1287	1461607	1462930	YP_049393.1	citM	Mg(2+)/citrate complex transporter
ECA1288	1463403	1464303	YP_049394.1	-	LysR-family transcriptional regulator
DdaI53					
======					

ECA1321	1495195	1495876	YP_049427.1	-	hypothetical protein
ECA1322	1495946	1496435	YP_049428.1	-	hypothetical protein
Ddal54					
======					
ECA1344	1523467	1523674	YP_049450.1	-	hypothetical protein
ECA1345	1523817	1525473	YP_049451.1	-	ABC transporter permease protein
ECA1346	1525673	1526654	YP_049452.1	-	ABC transporter substrate binding protein
ECA1347	1526671	1527400	YP_049453.1	-	ABC transporter ATP-binding protein
Ddal55					
======					
ECA1417	1607968	1609105	YP_049523.1	wza	putative polysaccharide export protein
ECA1418	1609113	1609548	YP_049524.1	wzb	probable protein-tyrosine-phosphatase
ECA1419	1609564	1611733	YP_049525.1	wzc	tyrosine kinase
ECA1420	1611940	1613374	YP_049526.1	rfbP	undecaprenyl-phosphate galactosephosphotransferase
ECA1421	1613497	1614457	YP_049527.1	rfbI	CDP-6-deoxy-delta-3,4-glucoseen reductase
ECA1422	1614551	1615325	YP_049528.1	rfbF	glucose-1-phosphate cytidylyltransferase
ECA1423	1615315	1616422	YP_049529.1	rfbG	CDP-glucose 4,6-dehydratase
ECA1424	1616421	1617735	YP_049530.1	rfbH	CDP-4-keto-6-deoxy-D-glucose-3-dehydratase
ECA1425	1617929	1618712	YP_049531.1	hpcH	putative 2,4-dihydroxyhept-2-ene-1,7-dioic acid aldolase
ECA1426	1618721	1620518	YP_049532.1	-	putative thiamine pyrophosphate enzyme
ECA1427	1620523	1621459	YP_049533.1	-	putative dTDP-glucose 4,6-dehydratase
ECA1428	1621486	1622377	YP_049534.1	nahO	acetaldehyde dehydrogenase
ECA1429	1622381	1623413	YP_049535.1	nahM	4-hydroxy-2-ketovalerate aldolase
ECA1430	1623415	1624471	YP_049536.1	-	putative dTDP-glucose 4-6-dehydratase
ECA1431	1624708	1626028	YP_049537.1	rfbX	putative O-antigen transporter
ECA1432	1625990	1626770	YP_049538.1	-	putative glycosyl transferase
ECA1433	1626766	1627393	YP_049539.1	-	putative acyl transferase
ECA1434	1627408	1628554	YP_049540.1	-	putative glycosyl transferase
ECA1435	1628550	1629753	YP_049541.1	rfc	putative O-antigen polymerase
ECA1436	1629785	1630895	YP_049542.1	rfbU	putative glycosyl transferase
ECA1437	1631013	1631931	YP_049543.1	rfbN	O antigen biosynthesis rhamnosyltransferase rfbn
ECA1438	1631951	1633361	YP_049544.1	rfbM	mannose-1-phosphate guanylyltransferase
ECA1439	1633373	1634744	YP_049545.1	rfbK	phosphomannomutase
ECA1440	1634819	1635671	YP_049546.1	rfbD	dTDP-4-dehydrorhamnose reductase
ECA1441	1635667	1636204	YP_049547.1	rfbC	dTDP-6-deoxy-D-glucose-3,5 epimerase
Ddal56					
======					
ECA1466	1666598	1666913	YP_049572.1	-	hypothetical protein
ECA1467	1666912	1667191	YP_049573.1	-	hypothetical protein
Ddal57					
======					
ECA1477	1673637	1673904	YP_049582.1	-	hypothetical protein
ECA1478	1674048	1674948	YP_049583.1	-	AraC-family transcriptional regulator
ECA1479	1675100	1676003	YP_049584.1	-	LysR-family transcriptional regulator
ECA1480	1676094	1677111	YP_049585.1	-	putative zinc-binding oxidoreductase
ECA1481	1677772	1678411	YP_049586.1	-	hypothetical protein
Ddal58					
======					
ECA1485	1680732	1681461	YP_049590.1	-	putative lipoprotein
ECA1486	1681841	1683506	YP_049591.1	-	ABC transporter ATP-binding protein
ECA1487	1683592	1704739	YP_049592.1	-	non-ribosomal peptide synthetase
ECA1488	1704731	1727303	YP_049593.1	-	non-ribosomal peptide synthetase
ECA1489	1728128	1729391	YP_049594.1	lacY	galactoside permease
ECA1490	1729533	1732656	YP_049595.1	lacZ	beta-D-galactosidase
Ddal59					
======					
ECA1509	1754694	1756374	YP_049611.1	-	methyl-accepting chemotaxis protein
ECA1510	1756919	1758824	YP_049612.1	-	putative iron permease
Ddal60					
======					
ECA1521	1770115	1771012	YP_049623.1	-	hypothetical protein
ECA1522	1771276	1771549	YP_049624.1	-	hypothetical protein
Ddal61					
======					
ECA1574	1830585	1831821	YP_049675.1	-	probable transporter
ECA1575	1831875	1832349	YP_049676.1	-	MerR-family transcriptional regulator
ECA1576	1832391	1832559	YP_049677.1	-	hypothetical protein
ECA1577	1832681	1833305	YP_049678.1	-	putative lipoprotein
ECA1578	1833670	1834210	YP_049679.1	-	hypothetical protein
ECA1579	1834369	1835260	YP_049680.1	-	2-dehydropantoate 2-reductase
ECA1580	1835449	1835932	YP_049681.1	-	putative cold-shock protein
ECA1581	1836064	1836310	YP_049682.1	-	hypothetical protein
ECA1582	1836536	1836671	YP_049683.1	-	putative transposase (partial)
ECA1583	1836750	1837002	YP_049684.1	-	putative integrase
ECA1584	1837756	1837906	YP_049685.1	-	hypothetical protein
ECA1585	1837902	1839441	YP_049686.1	-	hypothetical protein
ECA1586	1840175	1840934	YP_049687.1	-	probable short chain dehydrogenase
ECA1587	1840981	1841674	YP_049688.1	-	probable glutathione-S-transferase
ECA1588	1841726	1842110	YP_049689.1	-	hypothetical protein
ECA1589	1842240	1847037	YP_049690.1	lhr	putative ATP-dependent helicase Lhr
ECA1590	1847044	1847386	YP_049691.1	-	hypothetical protein
ECA1591	1847461	1848211	YP_049692.1	-	hypothetical protein
ECA1592	1848238	1849141	YP_049693.1	-	putative acetyl-hydrolase

ECA1593	1849383	1850334	YP_049694.1	-	AraC-family transcriptional regulator
ECA1594	1850489	1850948	YP_049695.1	-	hypothetical protein
ECA1595	1851096	1851453	YP_049696.1	-	hypothetical protein
ECA1596	1851991	1853839	YP_049697.1	-	hypothetical protein
ECA1597	1853835	1854879	YP_049698.1	-	hypothetical protein
ECA1598	1855522	1856875	YP_049699.1	-	hypothetical protein

DdaI62
======

ECA1628	1881481	1882006	YP_049729.1	-	hypothetical protein
ECA1629	1883004	1883862	YP_049730.1	-	AraC-family transcriptional regulator
ECA1632	1886844	1888533	YP_049731.1	-	hypothetical protein
ECA1633	1888809	1889685	YP_049732.1	-	hypothetical protein
ECA1634	1890003	1890399	YP_049733.1	-	hypothetical protein
ECA1635	1890476	1890773	YP_049734.1	-	hypothetical protein
ECA1636	1890822	1891059	YP_049735.1	-	hypothetical protein
ECA1637	1891085	1891436	YP_049736.1	-	hypothetical protein
ECA1638	1891504	1891747	YP_049737.1	-	Hns-like DNA-binding protein
ECA1639	1891964	1893239	YP_049738.1	-	hypothetical protein
ECA1640	1893332	1893812	YP_049739.1	-	hypothetical protein
ECA1641	1893899	1894037	YP_049740.1	-	hypothetical protein
ECA1642	1894271	1894526	YP_049741.1	-	hypothetical protein
ECA1643	1894836	1895358	YP_049742.1	-	hypothetical protein
ECA1644	1895383	1896118	YP_049743.1	-	putative DNA-binding protein
ECA1645	1897720	1898485	YP_049744.1	-	hypothetical protein
ECA1646	1898491	1899034	YP_049745.1	-	hypothetical protein
ECA1647	1899337	1900024	YP_049746.1	-	hypothetical protein
ECA1648	1900097	1900667	YP_049747.1	-	hypothetical protein
ECA1649	1900958	1901330	YP_049748.1	-	hypothetical protein
ECA1650	1901395	1901848	YP_049749.1	-	hypothetical protein
ECA1651	1901912	1902167	YP_049750.1	-	hypothetical protein
ECA1652	1902247	1903087	YP_049751.1	-	transposase
ECA1653	1903089	1903356	YP_049752.1	-	transposase
ECA1654	1903581	1903959	YP_049753.1	-	hypothetical protein
ECA1655	1904591	1905143	YP_049754.1	-	putative lipoprotein
ECA1656	1905153	1905429	YP_049755.1	-	hypothetical protein
ECA1657	1905550	1907395	YP_049756.1	-	hypothetical protein
ECA1658	1908103	1909087	YP_049757.1	-	hypothetical protein
ECA1659	1909270	1910731	YP_049758.1	-	probable plasmid-related protein
ECA1660	1911407	1912160	YP_049759.1	-	hypothetical protein
ECA1661	1912379	1913045	YP_049760.1	-	hypothetical protein
ECA1662	1913611	1913905	YP_049761.1	-	hypothetical protein
ECA1663	1913933	1914218	YP_049762.1	-	hypothetical protein
ECA1664	1914323	1914683	YP_049763.1	-	putative lipoprotein
ECA1665	1914762	1915161	YP_049764.1	hns1	DNA-binding protein Hns
ECA1666	1915261	1915492	YP_049765.1	-	hypothetical protein
ECA1667	1915723	1916281	YP_049766.1	-	putative type IV pilin protein precursor
ECA1668	1916387	1917980	YP_049767.1	-	putative type IV prepilin
ECA1669	1918142	1919885	YP_049768.1	-	hypothetical protein
ECA1669A	1919881	1920076	YP_049769.1	-	hypothetical protein
ECA1670	1920491	1920980	YP_049770.1	-	hypothetical protein
ECA1671	1921647	1921920	YP_049771.1	-	hypothetical protein
ECA1672	1922528	1922717	YP_049772.1	-	hypothetical protein
ECA1672A	1922742	1922949	YP_049773.1	-	hypothetical protein
ECA1673	1923047	1923206	YP_049774.1	-	integrase (partial)
ECA1674	1923211	1923496	YP_049775.1	-	hypothetical protein
ECA1675	1923639	1923981	YP_049776.1	-	putative integrase (partial)
ECA1676	1923956	1924289	YP_049777.1	chpA	toxin ChpA
ECA1677	1924288	1924540	YP_049778.1	chpR	suppressor of growth inhibitory protein ChpA
ECA1678	1925153	1925486	YP_049779.1	-	hypothetical protein
ECA1679	1925681	1926959	YP_049780.1	intB	phage integrase
ECA1680	1927324	1928122	YP_049781.1	-	hypothetical protein
ECA1681	1928678	1930007	YP_049782.1	-	hypothetical protein

DdaI63
======

ECA1704	1951838	1952513	YP_049805.1	flgD	flagellar basal body rod modification protein
ECA1705	1952588	1953806	YP_049806.1	flgE	flagellar hook protein FlgE

DdaI64
======

ECA1710	1957412	1958375	YP_049811.1	flgJ	peptidoglycan hydrolase
ECA1711	1958501	1960208	YP_049812.1	flgK	flagellar hook-associated protein FlgK
ECA1712	1960239	1961193	YP_049813.1	flgL	flagellar hook-associated protein FlgL

DdaI65
======

ECA1729	1973614	1974025	YP_049829.1	fliS	flagellar protein FliS
ECA1730	1974064	1975483	YP_049830.1	fliD	flagellar capping protein
ECA1731	1975727	1976600	YP_049831.1	fliC	flagellin
ECA1732	1976823	1980243	YP_049832.1	-	hypothetical protein
ECA1733	1980554	1981667	YP_049833.1	vioA	nucleotide sugar transaminase
ECA1734	1981772	1982474	YP_049834.1	-	hypothetical protein
ECA1735	1982476	1983436	YP_049835.1	-	hypothetical protein
ECA1736	1983435	1984404	YP_049836.1	-	hypothetical protein
ECA1737	1984403	1984844	YP_049837.1	-	putative acetyltransferase

DdaI66

======
ECA1762 2010924 2011254 YP_049862.1 - hypothetical protein
ECA1763 2011394 2012054 YP_049863.1 - hypothetical protein
ECA1764 2012811 2013066 YP_049864.1 - hypothetical protein
ECA1765 2013099 2013261 YP_049865.1 - hypothetical protein
ECA1766 2013547 2013970 YP_049866.1 - hypothetical protein
ECA1767 2013993 2014578 YP_049867.1 - Isochorismatase family protein
ECA1768 2014582 2015038 YP_049868.1 - putative acetyltransferase
ECA1769 2015202 2015631 YP_049869.1 - hypothetical protein
ECA1770 2015679 2016186 YP_049870.1 - hypothetical protein
ECA1771 2016269 2016455 YP_049871.1 - hypothetical protein
ECA1772 2016610 2016910 YP_049872.1 - hypothetical protein
ECA1773 2017068 2017350 YP_049873.1 - hypothetical protein
ECA1774 2018183 2019848 YP_049874.1 - methyl-accepting chemotaxis protein
DdaI67
======
ECA1783 2030936 2031491 YP_049883.1 - hypothetical protein
ECA1784 2031946 2032201 YP_049884.1 bssS biofilm formation regulatory protein BssS
ECA1785 2032437 2032695 YP_049885.1 - hypothetical protein
ECA1786 2032783 2033023 YP_049886.1 dinI DNA damage-inducible protein I
DdaI68
======
ECA1828 2075554 2075833 YP_049925.1 - hypothetical protein
ECA1829 2076004 2076721 YP_049926.1 - putative Crp/Fnr-family of transcriptional regulator
DdaI69
======
ECA1845 2091755 2092574 YP_049942.1 hmuT hemin-binding periplasmic protein
ECA1846 2092570 2093620 YP_049943.1 hemS hemin transport protein
ECA1847 2093826 2095149 YP_049944.1 aglB 6-phospho-alpha-glucosidase
ECA1848 2095151 2096741 YP_049945.1 aglA PTS system, alpha-glucoside-specific IIBC component
ECA1849 2097008 2097755 YP_049946.1 - GntR-family transcriptional regulator
DdaI70
======
ECA1872 2127145 2128786 YP_049969.1 - putative porin
ECA1873 2128872 2130063 YP_049970.1 - putative glycosyl hydrolase exoenzyme
ECA1874 2130167 2131181 YP_049971.1 - putative inosine-uridine preferring nucleoside hydrolase
ECA1875 2131620 2133054 YP_049972.1 nrfA cytochrome c nitrite reductase
DdaI71
======
ECA1900 2152116 2153838 YP_049997.1 narQ nitrate/nitrite sensor protein NarQ
ECA1901 2153925 2154558 YP_049998.1 narP DNA-binding response regulator in two-component regulatory system with NarQ or NarX
ECA1902 2154855 2155701 YP_049999.1 - AraC-family transcriptional regulator
ECA1903 2155749 2157117 YP_050000.1 - hypothetical protein
ECA1904 2157265 2158261 YP_050001.1 - probable zinc-binding dehydrogenase
DdaI72
======
ECA1970 2237015 2237987 YP_050065.1 - putative thiamine biosynthesis protein
ECA1971 2238072 2238627 YP_050066.1 aidA hypothetical protein
DdaI73
======
ECA1996 2264210 2264807 YP_050090.1 col colicin
ECA1997 2264868 2265129 YP_050091.1 imm colicin immunity protein
ECA1998 2265485 2265758 YP_050092.1 - hypothetical protein
ECA1999 2266316 2266607 YP_050093.1 - putative lipoprotein
DdaI74
======
ECA2007 2279510 2280350 YP_050101.1 - putative protease
ECA2008 2280395 2282174 YP_050102.1 - putative signaling protein
ECA2009 2282646 2283504 YP_050103.1 - putative exported protease
ECA2010 2283755 2284052 YP_050104.1 - hypothetical protein
DdaI75
======
ECA2019 2291855 2294414 YP_050113.1 - hypothetical protein
ECA2020 2294423 2297000 YP_050114.1 - hypothetical protein
ECA2021 2297112 2298807 YP_050115.1 - putative sulfate transporter YchM
ECA2022 2298970 2300071 YP_050116.1 chaA calcium/sodium:proton antiporter
ECA2022A 2300085 2300208 YP_050117.1 - putative surface antigen protein
ECA2023 2300400 2301204 YP_050118.1 - putative siderophore-interacting protein
DdaI76
======
ECA2042 2323395 2323677 YP_050137.1 - hypothetical protein
ECA2043 2323654 2324143 YP_050138.1 - hypothetical protein
ECA2044 2324295 2324544 YP_050139.1 - hypothetical protein
ECA2045 2324721 2325750 YP_050140.1 - putative NAD dependent epimerase/dehydratase
ECA2046 2325746 2328827 YP_050141.1 - putative bifunctional enzyme including aminotransferase and chitin synthase
ECA2047 2328860 2330060 YP_050142.1 - hypothetical protein
ECA2048 2330076 2332041 YP_050143.1 - hypothetical protein
ECA2049 2332155 2332908 YP_050144.1 - hypothetical protein
DdaI77
======
ECA2061 2346978 2348505 YP_050156.1 - methyl-accepting chemotaxis protein

ECA2062	2348944	2350252	YP_050157.1	-	putative phosphatase

DdaI78

======

ECA2067	2354961	2355252	YP_050162.1	bor	lipoprotein
ECA2068	2355814	2357275	YP_050163.1	-	putative permease
ECA2069	2357360	2358035	YP_050164.1	-	TetR-family transcriptional regulator
ECA2070	2358132	2359305	YP_050165.1	-	major facilitator family transporter
ECA2071	2359588	2360806	YP_050166.1	-	putative cytochrome P450
ECA2072	2360795	2361239	YP_050167.1	-	flavodoxin
ECA2073	2361326	2362568	YP_050168.1	-	probable oxidoreductase

DdaI79

======

ECA2090	2379835	2380900	YP_050185.1	hrpS	sigma-54-dependent enhancer-binding protein
ECA2091	2380996	2382616	YP_050186.1	-	putative signaling membrane protein

DdaI80

======

ECA2111	2398202	2398526	YP_050206.1	-	potential HrpW-specific chaperone
ECA2112	2398527	2399967	YP_050207.1	hrpW	type III effector protein
ECA2113	2400143	2404988	YP_050208.1	dspE	putative avirulence protein
ECA2114	2405022	2405445	YP_050209.1	dspF	putative avirulence protein
ECA2115	2405454	2406120	YP_050210.1	-	putative transcriptional regulator
ECA2116	2406457	2408131	YP_050211.1	hecB	putative hemolysin activator protein
ECA2117	2408180	2411585	YP_050212.1	hecA1	hemolysin/hemagglutinin-like protein
ECA2118	2411692	2426503	YP_050213.1	hecA2	hemolysin/hemagglutinin-like protein HecA precursor
ECA2119	2426504	2426837	YP_050214.1	-	hypothetical protein
ECA2120	2426873	2427218	YP_050215.1	-	hypothetical protein

DdaI81

======

ECA2126	2428960	2429380	YP_050221.1	-	hypothetical protein
ECA2127	2429381	2429813	YP_050222.1	-	hypothetical protein
ECA2128	2430107	2431115	YP_050223.1	-	hypothetical protein
ECA2129	2431181	2431634	YP_050224.1	-	hypothetical protein
ECA2130	2431736	2432021	YP_050225.1	-	hypothetical protein
ECA2131	2432246	2432537	YP_050226.1	-	hypothetical protein
ECA2132	2432550	2433018	YP_050227.1	-	hypothetical protein
ECA2133	2433252	2433552	YP_050228.1	-	hypothetical protein
ECA2134	2433583	2434705	YP_050229.1	-	ABC transporter, substrate binding protein
ECA2135	2434873	2436580	YP_050230.1	-	periplasmic pectate lyase
ECA2136	2436679	2437444	YP_050231.1	-	hypothetical protein
ECA2137	2437562	2438381	YP_050232.1	-	hypothetical protein
ECA2138	2438377	2438554	YP_050233.1	-	hypothetical protein
ECA2139	2439080	2439257	YP_050234.1	-	hypothetical protein
ECA2140	2439404	2440412	YP_050235.1	-	putative oxidoreductase
ECA2141	2440592	2442395	YP_050236.1	-	amidase

DdaI82

======

ECA2149	2452531	2452981	YP_050244.1	-	hypothetical protein
ECA2150	2453336	2453636	YP_050245.1	-	hypothetical protein

DdaI83

======

ECA2159	2461265	2461811	YP_050254.1	hpaC	putative 4-hydroxyphenylacetate 3-monooxygenase, reductase component
ECA2160	2462085	2463282	YP_050255.1	-	putative peptidase
ECA2161	2463566	2464547	YP_050256.1	-	putative luciferase-like monooxygenase
ECA2162	2464655	2465162	YP_050257.1	-	hypothetical protein
ECA2163	2466051	2468013	YP_050258.1	-	autotransporter
ECA2164	2468108	2469152	YP_050259.1	ascG	asc operon repressor

DdaI84

======

ECA2169	2473903	2474320	YP_050264.1	-	hypothetical protein
ECA2170	2474316	2475462	YP_050265.1	-	hypothetical protein
ECA2171	2475467	2476352	YP_050266.1	-	hypothetical protein
ECA2172	2476853	2477447	YP_050267.1	-	hypothetical protein
ECA2173	2477691	2478756	YP_050268.1	-	hypothetical protein
ECA2174	2479014	2479443	YP_050269.1	-	hypothetical protein
ECA2175	2479529	2479928	YP_050270.1	-	hypothetical protein
ECA2176	2479938	2480340	YP_050271.1	-	hypothetical protein
ECA2178	2480846	2481182	YP_050272.1	-	prophage integrase (partial)
ECA2179	2481463	2481994	YP_050273.1	-	hypothetical protein
ECA2180	2481996	2484267	YP_050274.1	-	hypothetical protein
ECA2181	2484488	2484836	YP_050275.1	-	Bacteriophage polarity suppression protein

DdaI85

======

ECA2208	2507443	2507977	YP_050301.1	ogt	methylated-DNA--protein-cysteine methyltransferase
ECA2209	2508507	2509803	YP_050302.1	-	putative oxidoreductase
ECA2210	2509911	2511231	YP_050303.1	-	periplasmic binding protein precursor
ECA2211	2511297	2511822	YP_050304.1	-	copper-zinc superoxide dismutase
ECA2212	2511875	2513105	YP_050305.1	-	putative cyclopropane-fatty-acyl-phospholipid synthase
ECA2213	2513101	2513827	YP_050306.1	-	hypothetical protein
ECA2214	2513819	2515088	YP_050307.1	-	putative amine oxidoreductase
ECA2215	2515093	2515801	YP_050308.1	-	putative short-chain dehydrogenase
ECA2216	2515806	2516286	YP_050309.1	-	hypothetical protein

DdaI86

======
ECA2220 2523661 2524351 YP_050313.1 - putative cellulase
ECA2221 2524681 2525110 YP_050314.1 osmC osmotically inducible protein C
ECA2222 2525398 2525785 YP_050315.1 cybC putative soluble cytochrome b562
ECA2223 2525892 2526447 YP_050316.1 - TetR-family transcriptional regulator
ECA2224 2526582 2527779 YP_050317.1 - putative transporter
ECA2225 2527928 2528417 YP_050318.1 - putative acetyltransferase
ECA2225A 2528537 2528708 YP_050319.1 - hypothetical protein
ECA2226 2528724 2530560 YP_050320.1 - methyl-accepting chemotaxis protein
ECA2227 2530748 2531648 YP_050321.1 - chromosome replication initiation inhibitor protein
ECA2228 2531644 2531911 YP_050322.1 - hypothetical protein
ECA2229 2532269 2532398 YP_050323.1 - hypothetical protein
ECA2230 2532527 2533646 YP_050324.1 - putative NADH-dependent flavin oxidoreductase
ECA2231 2533658 2534552 YP_050325.1 - hypothetical protein
ECA2232 2534899 2535796 YP_050326.1 - LysR-family transcriptional regulator
ECA2233 2535824 2536745 YP_050327.1 - probable hydrolase
ECA2234 2536812 2537412 YP_050328.1 - hypothetical protein
DdaI87
======
ECA2237 2539269 2539632 YP_050332.1 flxA hypothetical protein
ECA2238 2539821 2540028 YP_050333.1 - hypothetical protein
ECA2239 2540217 2540505 YP_050334.1 - hypothetical protein
ECA2240 2540510 2541806 YP_050335.1 - putative lipoprotein
ECA2241 2541854 2542190 YP_050336.1 - hypothetical protein
ECA2242 2542220 2542454 YP_050337.1 - hypothetical protein
ECA2243 2542866 2544249 YP_050338.1 dat diaminobutyrate--2-oxoglutarate aminotransferase
ECA2244 2544267 2545785 YP_050339.1 ddc L-2,4-diaminobutyrate decarboxylase
ECA2245 2545856 2546087 YP_050340.1 - 4-oxalocrotonate tautomerase
ECA2246 2546334 2547111 YP_050341.1 - hypothetical protein
ECA2247 2547154 2548117 YP_050342.1 - Arac-family transcriptional regulator
ECA2248 2548322 2549147 YP_050343.1 - mannosyl-3-phosphoglycerate phosphatase
DdaI88
======
ECA2253 2553107 2553458 YP_050348.1 - hypothetical protein
ECA2254 2553610 2554228 YP_050349.1 - putative lipoprotein
ECA2255 2554333 2554633 YP_050350.1 - hypothetical protein
ECA2256 2554697 2556170 YP_050351.1 - putative transporter
DdaI89
======
ECA2264 2564897 2566448 YP_050359.1 - hypothetical protein
ECA2265 2566549 2567527 YP_050360.1 - hypothetical protein
DdaI90
======
ECA2294 2599459 2601298 YP_050389.1 - putative glycosyl transferase
ECA2295 2601616 2602654 YP_050390.1 - AraC-family transcriptional regulator
DdaI91
======
ECA2306 2612726 2614289 YP_050401.1 - probable phage integrase
ECA2307 2614332 2614647 YP_050402.1 - putative phage-related protein
ECA2308 2614680 2615379 YP_050403.1 - putative phage-related protein
ECA2309 2616137 2616857 YP_050404.1 - putative phage-related protein
ECA2310 2616853 2617060 YP_050405.1 - hypothetical protein
ECA2311 2617182 2617593 YP_050406.1 - hypothetical protein
DdaI92
======
ECA2352 2665343 2666324 YP_050447.1 - putative 6-phosphofructokinase isozyme II
ECA2353 2666334 2666628 YP_050448.1 - hypothetical protein
ECA2354 2666641 2667979 YP_050449.1 ulaA ascorbate-specific PTS system enzyme IIC
ECA2355 2668043 2668322 YP_050450.1 - hypothetical protein
ECA2356 2668328 2668766 YP_050451.1 - putative sugar phosphotransferase enzyme IIa component
ECA2357 2668762 2669296 YP_050452.1 - putative kinase
ECA2358 2669332 2670232 YP_050453.1 - putative aldolase
DdaI93
======
ECA2381 2692520 2693453 YP_050476.1 - LysR-family transcriptional regulator
ECA2382 2693564 2694197 YP_050477.1 - putative NADP oxidoreductase coenzyme F420-dependent
protein
ECA2383 2694344 2695058 YP_050478.1 - putative isochorismatase
DdaI94
======
ECA2409 2721433 2722009 YP_050504.1 - hypothetical protein
ECA2410 2722185 2723580 YP_050505.1 - putative sodium:dicarboxylate symporter
DdaI95
======
ECA2430 2744003 2745227 YP_050525.1 - hypothetical protein
ECA2431 2745410 2746160 YP_050526.1 - two-component response regulator
ECA2432 2746156 2747899 YP_050527.1 - two-component sensor kinase
ECA2433 2748046 2750215 YP_050528.1 - putative signaling protein
ECA2434 2750607 2750931 YP_050529.1 - hypothetical protein
ECA2435 2751258 2751993 YP_050530.1 rdgA regulator of pectin lyase production
ECA2437 2752446 2752797 YP_050531.1 rdgB regulator of pectin lyase production
ECA2438 2753271 2754381 YP_050532.1 - putative electron transport protein
DdaI96

======
ECA2454 2769656 2770193 YP_050547.1 ogt probable methylated DNA--protein-cysteine methyltransferase
ECA2455 2770375 2771476 YP_050548.1 fsr fosmidomycin resistance protein
ECA2456 2771526 2771685 YP_050549.1 - probable transcriptional regulator (partial)
DdaI97
======
ECA2465 2782997 2783189 YP_050558.1 - hypothetical protein
ECA2466 2783207 2783903 YP_050559.1 - hypothetical protein
ECA2467 2783942 2784188 YP_050560.1 - hypothetical protein
DdaI98
======
ECA2497 2814295 2815513 YP_050589.1 - putative signal transduction protein
ECA2498 2815600 2816095 YP_050590.1 - hypothetical protein
ECA2499 2816170 2816962 YP_050591.1 - hypothetical protein
ECA2500 2816961 2819196 YP_050592.1 - putative DNA-binding protein
ECA2501 2819422 2820118 YP_050593.1 - hypothetical protein
ECA2502 2820114 2820354 YP_050594.1 - hypothetical protein
ECA2503 2820770 2820968 YP_050595.1 - putative phage-related protein
ECA2504 2821763 2822402 YP_050596.1 - resolvase
ECA2505 2823148 2823316 YP_050597.1 - hypothetical protein
ECA2506 2823640 2824555 YP_050598.1 - hypothetical protein
ECA2508 2825270 2826881 YP_050599.1 - putative integrase/recombinase
DdaI99
======
ECA2513 2830277 2830577 YP_050604.1 - hypothetical protein
ECA2514 2830706 2831090 YP_050605.1 - hypothetical protein
ECA2515 2831104 2831689 YP_050606.1 - hypothetical protein
DdaI100
=======
ECA2598 2935460 2935850 YP_050689.1 - hypothetical protein
ECA2599 2935892 2936141 YP_050690.1 - bacteriophage regulatory protein
ECA2600 2936177 2937215 YP_050691.1 - bacteriophage late gene control protein D
ECA2601 2937467 2938652 YP_050692.1 - major tail sheath protein FI
ECA2602 2938652 2939168 YP_050693.1 - major tail tube protein FII
ECA2603 2939281 2939590 YP_050694.1 - putative phage tail protein E
ECA2604 2939610 2939751 YP_050695.1 - putative phage tail protein E'
ECA2605 2939737 2942668 YP_050696.1 - putative bacteriophage tail fiber protein T (tape measure)
ECA2606 2942680 2943172 YP_050697.1 - putative bacteriophage tail fiber protein U
ECA2607 2943292 2943910 YP_050698.1 - putative bacteriophage tail fiber assembly protein G
ECA2608 2943909 2945520 YP_050699.1 - probable bacteriophage variable tail fiber protein H
ECA2609 2945516 2946122 YP_050700.1 - putative phage tail protein I
ECA2610 2946114 2947011 YP_050701.1 - phage baseplate assembly protein J
ECA2611 2946997 2947366 YP_050702.1 - phage baseplate assembly protein W
ECA2612 2947362 2947944 YP_050703.1 - baseplate assembly protein V
ECA2613 2947940 2948579 YP_050704.1 - putative phage tail protein S
ECA2614 2948571 2949024 YP_050705.1 - phage tail completion protein R
ECA2615 2949163 2949604 YP_050707.1 - putative phage encoded host lysis, regulatory protein
ECA2616 2949600 2950143 YP_050708.1 - hypothetical protein
DdaI101
=======
ECA2620 2951269 2952163 YP_050712.1 - phage terminase, endonuclease small subunit M
ECA2621 2952214 2953264 YP_050713.1 - major phage capsid protein N precursor
ECA2622 2953288 2954122 YP_050714.1 - capsid scaffolding protein O
ECA2623 2954280 2956002 YP_050715.1 - phage terminase, ATPase subunit P
ECA2624 2956003 2957050 YP_050716.1 - phage capsid portal protein Q
ECA2625 2957425 2958115 YP_050717.1 - phage DNA methylase
ECA2626 2958117 2958444 YP_050718.1 - putative phage-related protein
ECA2627 2958536 2961047 YP_050719.1 - putative phage replication protein A
ECA2628 2961046 2962075 YP_050720.1 - conserved phage protein
ECA2629 2962067 2962916 YP_050721.1 - phage DNA adenine methylase
ECA2630 2962912 2963812 YP_050722.1 - hypothetical protein
ECA2631 2963808 2964042 YP_050723.1 - hypothetical protein
ECA2632 2964112 2964448 YP_050724.1 - hypothetical protein
ECA2633 2964528 2964654 YP_050725.1 - hypothetical protein
ECA2634 2964650 2964851 YP_050726.1 - phage-related hypothetical protein
ECA2635 2964847 2965210 YP_050727.1 - phage regulatory protein
ECA2636 2965318 2965618 YP_050728.1 - phage repressor protein C
ECA2637 2965684 2966671 YP_050729.1 - phage integrase
ECA2639 2967602 2969171 YP_050730.1 - putative hydantoinase
ECA2640 2969181 2970291 YP_050731.1 - hypothetical protein
ECA2641 2970343 2971648 YP_050732.1 - permease
ECA2642 2971921 2972863 YP_050733.1 - LysR-family transcriptional regulator
DdaI102
=======
ECA2654 2988413 2989868 YP_050745.1 - GntR-family transcriptional regulator
ECA2655 2989859 2990339 YP_050746.1 - hypothetical protein
DdaI103
=======
ECA2692 3027382 3028801 YP_050783.1 - putative flavin-containing amine oxidase
ECA2693 3028797 3029253 YP_050784.1 - hypothetical protein
ECA2694 3029318 3031103 YP_050785.1 - putative polyketide synthetase
ECA2695 3031132 3032050 YP_050786.1 - putative malonyl CoA-acyl carrier protein transacylase
ECA2696 3032046 3033456 YP_050787.1 ehpG putative phenazine antibiotic biosynthesis protein

ECA2697	3033455	3034517	YP_050788.1	ehpF	putative phenazine antibiotic biosynthesis protein
ECA2698	3034529	3035168	YP_050789.1	ehpE	putative phenazine antibiotic biosynthesis protein
ECA2699	3035177	3036023	YP_050790.1	ehpD	putative phenazine antibiotic biosynthesis protein
ECA2700	3036001	3036352	YP_050791.1	-	hypothetical protein
ECA2701	3036348	3038226	YP_050792.1	ehpC	putative phenazine antibiotic biosynthesis protein
ECA2702	3038222	3038843	YP_050793.1	ehpB	putative phenazine antibiotic biosynthesis protein
ECA2703	3038922	3039381	YP_050794.1	ehpA	putative phenazine antibiotic biosynthesis protein
ECA2704	3039427	3040207	YP_050795.1	-	short chain dehydrogenase
ECA2705	3040364	3040751	YP_050796.1	ehpR	putative phenazine antibiotic biosynthesis protein
Ddal104					
=======					
ECA2754	3095609	3098216	YP_050845.1	-	putative prophage primase
ECA2755	3098461	3098917	YP_050846.1	-	hypothetical protein
ECA2756	3099004	3100030	YP_050847.1	-	hypothetical protein
ECA2757	3100257	3100503	YP_050848.1	-	hypothetical protein
ECA2758	3100492	3100777	YP_050849.1	-	hypothetical protein
ECA2759	3100822	3101182	YP_050850.1	-	hypothetical protein
ECA2760	3101845	3102175	YP_050851.1	-	hypothetical protein
ECA2761	3102234	3102771	YP_050852.1	-	hypothetical protein
Ddal105					
=======					
ECA2775	3115104	3115545	YP_050866.1	-	hypothetical protein
ECA2776	3115833	3116145	YP_050867.1	-	hypothetical protein
ECA2777	3116137	3116467	YP_050868.1	-	hypothetical protein
Ddal106					
=======					
ECA2794	3134964	3135321	YP_050885.1	-	hypothetical protein
ECA2795	3135381	3135924	YP_050886.1	cyaB	adenylate cyclase
Ddal107					
=======					
ECA2840	3182763	3184236	YP_050931.1	-	hypothetical protein
ECA2841	3184359	3185100	YP_050932.1	-	GntR-family transcriptional regulator
ECA2842	3185327	3186677	YP_050933.1	pcaB	3-carboxy-cis,cis-muconate cycloisomerase
ECA2843	3186823	3188245	YP_050934.1	-	putative sodium:sulfate symporter
ECA2844	3188335	3188503	YP_050935.1	-	hypothetical protein
ECA2845	3188703	3190713	YP_050936.1	nosA	TonB dependent receptor
ECA2846	3190821	3191298	YP_050937.1	-	hypothetical protein
ECA2847	3191687	3191906	YP_050938.1	-	hypothetical protein
ECA2848	3192249	3193197	YP_050939.1	-	lLysR-family transcriptional regulator
ECA2849	3193308	3194337	YP_050940.1	-	metallo-beta-lactamase
ECA2850	3194781	3195210	YP_050941.1	mvpA	putative plasmid protein
ECA2851	3195209	3195440	YP_050942.1	mvpT	putative plasmid protein
ECA2852	3195533	3196367	YP_050943.1	-	hypothetical protein
ECA2853	3196484	3196805	YP_050944.1	-	hypothetical protein
ECA2854	3196874	3197252	YP_050945.1	-	hypothetical protein
ECA2855	3197248	3197722	YP_050946.1	-	hypothetical protein
ECA2856	3197844	3198195	YP_050947.1	-	hypothetical protein
ECA2857	3198252	3199086	YP_050948.1	-	hypothetical protein
ECA2858	3199215	3199407	YP_050949.1	-	hypothetical protein
ECA2859	3199452	3199596	YP_050950.1	-	hypothetical protein
ECA2860	3199574	3200462	YP_050951.1	-	hypothetical protein
ECA2861	3200557	3201589	YP_050952.1	-	hypothetical protein
ECA2862	3202359	3202950	YP_050953.1	-	hypothetical protein
ECA2863	3203054	3203267	YP_050954.1	-	hypothetical protein
ECA2864	3203359	3203785	YP_050955.1	-	hypothetical protein
ECA2865	3203939	3204521	YP_050956.1	-	hypothetical protein
Ddal108					
=======					
ECA2870	3212037	3212454	YP_050961.1	-	hypothetical protein
ECA2871	3212529	3212853	YP_050962.1	-	hypothetical protein
ECA2872	3213660	3215115	YP_050963.1	-	putative flagellin
ECA2873	3215244	3215712	YP_050964.1	-	putative transcriptional regulator
ECA2874	3216323	3217214	YP_050965.1	-	hypothetical protein
ECA2875	3217206	3220539	YP_050966.1	-	putative ATP-binding protein
ECA2876	3220531	3221371	YP_050967.1	-	putative phage-related protein
ECA2877	3221521	3223375	YP_050968.1	-	hypothetical protein
ECA2878	3223377	3225771	YP_050969.1	-	putative signal transduction protein
ECA2879	3226142	3227396	YP_050970.1	-	integrase
Ddal109					
=======					
ECA2884	3232085	3232373	YP_050975.1	-	hypothetical protein
ECA2885	3233242	3234655	YP_050976.1	cycA	D-alanine/D-serine/glycine permease
ECA2886	3234824	3235091	YP_050977.1	-	hypothetical protein
ECA2887	3235087	3235258	YP_050978.1	-	hypothetical protein
ECA2888	3235471	3235747	YP_050979.1	nac	nitrogen assimilation regulatory protein (partial)
ECA2889	3236380	3237667	YP_050980.1	intB	integrase
ECA2890	3237700	3238219	YP_050981.1	-	hypothetical protein
ECA2891	3238279	3239092	YP_050982.1	-	hypothetical protein
ECA2892	3239132	3239936	YP_050983.1	-	hypothetical protein
ECA2893	3241129	3241531	YP_050984.1	hns3	DNA-binding protein Hns
ECA2894	3241657	3242011	YP_050985.1	-	putative plasmid-related protein
ECA2895	3242010	3242289	YP_050986.1	-	hypothetical protein
ECA2896	3242491	3242674	YP_050987.1	-	hypothetical protein

ECA2897	3242707	3242992	YP_050988.1	-	hypothetical protein
ECA2898	3243721	3245125	YP_050989.1	-	putative plasmid mobilization protein
ECA2899	3245207	3246110	YP_050990.1	-	hypothetical protein
ECA2900	3246455	3247238	YP_050991.1	-	hypothetical protein
ECA2901	3248044	3248488	YP_050992.1	-	hypothetical protein
ECA2902	3248551	3248980	YP_050993.1	-	hypothetical protein
ECA2903	3249068	3249716	YP_050994.1	-	hypothetical protein
ECA2904	3249732	3250287	YP_050995.1	-	putative lipoprotein
ECA2905	3250921	3251299	YP_050996.1	-	hypothetical protein
ECA2906	3251566	3252769	YP_050997.1	-	hypothetical protein
ECA2907	3253050	3253389	YP_050998.1	-	hypothetical protein
ECA2908	3253485	3254250	YP_050999.1	-	putative plasmid replication protein
ECA2909	3255301	3255463	YP_051000.1	-	hypothetical protein
ECA2910	3255833	3256568	YP_051001.1	-	putative plasmid replication protein
ECA2911	3256753	3257866	YP_051002.1	-	putative DNA-binding protein
ECA2912	3257875	3258616	YP_051003.1	-	putative plasmid-related protein
ECA2913	3258797	3259049	YP_051004.1	-	putative DNA-binding protein
ECA2914	3259116	3259467	YP_051005.1	-	hypothetical protein
ECA2915	3259506	3259770	YP_051006.1	-	hypothetical protein
ECA2916	3259823	3260120	YP_051007.1	-	hypothetical protein
ECA2917	3260199	3260514	YP_051008.1	-	hypothetical protein
ECA2918	3260546	3260765	YP_051009.1	-	putative phage-related protein
ECA2919	3260914	3261778	YP_051010.1	-	hypothetical protein
ECA2920	3261770	3261941	YP_051011.1	-	hypothetical protein
ECA2921	3262079	3263492	YP_051012.1	-	hypothetical protein
ECA2922	3264766	3265660	YP_051013.1	-	LysR-family transcriptional regulator
ECA2923	3265815	3266967	YP_051014.1	-	putative NADH:flavin oxidoreductase
ECA2924	3267166	3267820	YP_051015.1	-	putative lipoprotein
ECA2925	3268213	3268972	YP_051016.1	-	probable short chain dehydrogenase
ECA2926	3269019	3269145	YP_051017.1	-	putative glutathione-S-transferase (partial)
ECA2927	3269196	3270420	YP_051018.1	-	probable transporter
Ddal110					
=======					
ECA2931	3276182	3276797	YP_051022.1	-	TetR-family transcriptional regulator
ECA2932	3276883	3278356	YP_051023.1	-	putative outer membrane efflux lipoprotein
ECA2933	3278434	3279220	YP_051024.1	-	probable short-chain dehydrogenase
ECA2934	3279352	3279733	YP_051025.1	-	putative transcriptional regulator
ECA2935	3279844	3280585	YP_051026.1	-	hypothetical protein
ECA2936	3280603	3281122	YP_051027.1	nifQ	nitrogen fixation protein
Ddal111					
=======					
ECA2940	3286144	3286675	YP_051031.1	nifF	flavodoxin FldA
ECA2941	3286908	3287706	YP_051032.1	nifM	nitrogen fixation protein
Ddal112					
=======					
ECA2950	3295377	3295986	YP_051041.1	-	TetR-family regulatory protein
ECA2951	3296145	3297336	YP_051042.1	-	probable transporter
Ddal113					
=======					
ECA2972	3322878	3324231	YP_051063.1	-	putative hydrolase
ECA2973	3324256	3325147	YP_051064.1	-	LysR-family transcriptional regulator
ECA2974	3325374	3325824	YP_051065.1	-	MutT-like protein
ECA2975	3325953	3326604	YP_051066.1	-	hypothetical protein
ECA2976	3326721	3327630	YP_051067.1	-	LysR-family transcriptional regulator
ECA2977	3327795	3328329	YP_051068.1	-	hypothetical protein
ECA2978	3328333	3328660	YP_051069.1	-	hypothetical protein
ECA2979	3328673	3329513	YP_051070.1	-	probable hydrolase
ECA2980	3329724	3330060	YP_051071.1	-	hypothetical protein
ECA2981	3330104	3332162	YP_051072.1	-	hypothetical protein
ECA2982	3332181	3332514	YP_051073.1	emrE	multidrug efflux protein
ECA2983	3332534	3332705	YP_051074.1	-	hypothetical protein
ECA2984	3332712	3333801	YP_051075.1	xenA	flavoprotein xenobiotic reductase
ECA2985	3333852	3334194	YP_051076.1	-	ArsR-family transcriptional regulator
ECA2986	3334273	3334795	YP_051077.1	-	probable oxidase
ECA2987	3334879	3335344	YP_051078.1	-	hypothetical protein
ECA2988	3335433	3335667	YP_051079.1	-	conserved hypothetical protein (partial)
Ddal114					
=======					
ECA3075	3432148	3432922	YP_051165.1	-	putative fimbrial chaperone
ECA3076	3432965	3435347	YP_051166.1	-	putative fimbrial usher protein
ECA3077	3435351	3436362	YP_051167.1	-	hypothetical protein
ECA3078	3436354	3438550	YP_051168.1	fadJ	multifunctional fatty acid oxidation complex subunit alpha
ECA3079	3438546	3439860	YP_051169.1	fadI	3-ketoacyl-CoA thiolase
Ddal115					
=======					
ECA3088	3448806	3449568	YP_051178.1	-	hypothetical protein
ECA3089	3449696	3450047	YP_051179.1	-	hypothetical protein
ECA3090	3450487	3451027	YP_051180.1	-	hypothetical protein
Ddal116					
=======					
ECA3111	3471137	3473117	YP_051201.1	pehX	exo-poly-alpha-D-galacturonosidase
ECA3112	3473255	3474824	YP_051202.1	-	pectate lyase
Ddal117					

=======					
ECA3116	3478243	3479224	YP_051206.1	-	putative mandelate racemase/muconate lactonizing enzyme
ECA3117	3479266	3480127	YP_051207.1	dat	D-alanine aminotransferase
ECA3118	3480207	3482655	YP_051208.1	-	putative mechanosensitive ion channel protein
ECA3119	3483327	3484431	YP_051209.1	-	hypothetical protein
ECA3120	3484441	3486097	YP_051210.1	hsdS	subunit S of type I restriction-modification system
ECA3121	3486096	3487569	YP_051211.1	hsdM	subunit M of type I restriction-modification system
ECA3122	3487641	3490068	YP_051212.1	hsdR	type I restriction enzyme EcoEI R protein
DdaI118					
=======					
ECA3172	3542090	3543101	YP_051261.1	galR	DNA-binding transcriptional repressor
ECA3173	3543586	3544894	YP_051262.1	lamB	maltoporin
DdaI119					
=======					
ECA3180	3554168	3554510	YP_051269.1	-	PTS system, glucose-specific IIbc component (partial)
ECA3181	3554641	3556399	YP_051270.1	dld	D-lactate dehydrogenase
DdaI120					
=======					
ECA3202	3583504	3584407	YP_051291.1	-	LysR-family transcriptional regulator
ECA3203	3584422	3585196	YP_051292.1	-	hypothetical protein
ECA3204	3585400	3585751	YP_051293.1	-	hypothetical protein
ECA3205	3586090	3587221	YP_051294.1	-	putative exported choloylglycine hydrolase
ECA3206	3587319	3587535	YP_051295.1	-	transcriptional regulator
ECA3207	3587835	3588690	YP_051296.1	-	hypothetical protein
DdaI121					
=======					
ECA3213	3595534	3596425	YP_051302.1	-	AraC-family transcriptional regulator
ECA3214	3596585	3597341	YP_051303.1	-	probable short chain dehydrogenase
ECA3215	3597444	3598404	YP_051304.1	-	putative transport protein
DdaI122					
=======					
ECA3268	3672394	3674611	YP_051357.1	-	putative toxin secretion ATP-binding protein
ECA3269	3674597	3675959	YP_051358.1	-	HlyD family secretion protein
ECA3270	3676001	3677516	YP_051359.1	-	hypothetical protein
ECA3271	3678303	3679095	YP_051360.1	-	EAL domain containing protein involved in flagellar function
ECA3272	3679112	3679367	YP_051361.1	-	hypothetical protein
ECA3273	3679565	3679826	YP_051362.1	-	putative ferredoxin
DdaI123					
=======					
ECA3353	3770089	3770806	YP_051442.1	-	putative two-component response-regulatory protein YehT
ECA3354	3770827	3771202	YP_051443.1	-	hypothetical protein
DdaI124					
=======					
ECA3370	3786824	3787319	YP_051459.1	-	competence damage-inducible protein A
ECA3371	3787554	3787890	YP_051460.1	-	hypothetical protein
ECA3372	3788370	3788859	YP_051461.1	-	hypothetical protein
ECA3373	3788941	3789616	YP_051462.1	marC	multiple drug resistance protein MarC
ECA3374	3789807	3791406	YP_051463.1	-	putative signaling membrane protein
ECA3375	3791665	3792868	YP_051464.1	-	probable transporter
ECA3376	3792983	3793865	YP_051465.1	-	LysR-family transcriptional regulator
ECA3377	3794081	3794717	YP_051466.1	-	glutaredoxin 2
ECA3378	3794815	3795238	YP_051467.1	-	putative bacteriocin immunity protein
ECA3379	3795369	3796710	YP_051468.1	-	putative glycosyltransferase
ECA3380	3796721	3797654	YP_051469.1	pvcB	pyoverdine biosynthesis protein
ECA3381	3797643	3798624	YP_051470.1	pvcA	pyoverdine biosynthesis protein
ECA3381A	3798620	3798818	YP_051471.1	-	putative bacteriocin immunity protein
ECA3382	3799228	3799516	YP_051472.1	-	hypothetical protein
ECA3382A	3799503	3799749	YP_051473.1	-	hypothetical protein
ECA3383	3799872	3800157	YP_051474.1	-	hypothetical protein
ECA3384	3800169	3800646	YP_051475.1	-	hypothetical protein
ECA3385	3800656	3800947	YP_051476.1	-	hypothetical protein
ECA3386	3801261	3801546	YP_051477.1	-	hypothetical protein
ECA3387	3801550	3801913	YP_051478.1	-	hypothetical protein
DdaI125					
=======					
ECA3401	3814022	3814433	YP_051492.1	-	putative phage regulatory protein
ECA3402	3814511	3814736	YP_051493.1	-	hypothetical protein
ECA3403	3814801	3815131	YP_051494.1	-	hypothetical protein
ECA3404	3815127	3815565	YP_051495.1	-	hypothetical protein
DdaI126					
=======					
ECA3411	3821845	3822094	YP_051502.1	-	hypothetical protein
ECA3412	3822090	3822462	YP_051503.1	-	hypothetical protein
DdaI127					
=======					
ECA3420	3828783	3829221	YP_051511.1	-	hypothetical protein
ECA3421	3829217	3834074	YP_051512.1	-	Rhs protein
ECA3422	3834094	3835012	YP_051513.1	-	hypothetical protein
ECA3423	3835008	3835965	YP_051514.1	-	hypothetical protein
DdaI128					
=======					
ECA3446	3865038	3866442	YP_051536.1	oprJ	multidrug resistance outer membrane protein
ECA3447	3866438	3869582	YP_051537.1	mexB	multidrug resistance protein

ECA3448	3869664	3870837	YP_051538.1	mexC	multidrug resistance protein
ECA3449	3871026	3871593	YP_051539.1	nfxB	transcriptional regulator of multidrug resistance genes
ECA3450	3871999	3872587	YP_051540.1	-	hypothetical protein
ECA3451	3872982	3873474	YP_051541.1	-	hypothetical protein
ECA3452	3873466	3874063	YP_051542.1	fic	cell filamentation protein
ECA3453	3874070	3874256	YP_051543.1	-	putative phage capsid protein (partial)
ECA3454	3874252	3874501	YP_051544.1	-	bacteriophage regulatory protein
ECA3455	3874619	3874775	YP_051545.1	-	hypothetical protein
ECA3456	3875263	3876157	YP_051546.1	-	hypothetical protein
ECA3457	3876156	3876780	YP_051547.1	-	hypothetical protein
ECA3458	3876890	3877589	YP_051548.1	-	hypothetical protein
ECA3459	3877588	3878830	YP_051549.1	-	hypothetical protein
ECA3460	3878829	3880056	YP_051550.1	-	phage integrase
ECA3461	3880771	3881191	YP_051551.1	-	hypothetical protein
Ddal129					
=======					
ECA3480	3901552	3902074	YP_051570.1	-	hypothetical protein
ECA3481	3902192	3902711	YP_051571.1	-	hypothetical protein
Ddal130					
=======					
ECA3548	3976722	3978447	YP_051636.1	-	hypothetical protein
ECA3549	3978973	3979714	YP_051637.1	-	putative signaling protein
Ddal131					
=======					
ECA3556	3987236	3988169	YP_051644.1	-	LysR-family transcriptional regulator
ECA3557	3988267	3989257	YP_051645.1	-	metallo hydrolase
Ddal132					
=======					
ECA3625	4074060	4075893	YP_051713.1	-	hypothetical protein
ECA3626	4075861	4076878	YP_051714.1	-	putative type II/IV secretion system protein
Ddal133					
=======					
ECA3634	4081911	4082829	YP_051722.1	-	LysR-family transcriptional regulator
ECA3635	4082923	4084141	YP_051723.1	-	hypothetical protein
Ddal134					
=======					
ECA3657	4106202	4106496	YP_051745.1	-	hypothetical protein
ECA3658	4106531	4107239	YP_051746.1	-	hypothetical protein
ECA3659	4107616	4109308	YP_051747.1	-	hypothetical protein
Ddal135					
=======					
ECA3666	4116962	4117631	YP_051754.1	-	hypothetical protein
ECA3667	4117794	4118124	YP_051755.1	-	putative lipoprotein
Ddal136					
=======					
ECA3671	4121032	4121530	YP_051759.1	-	hypothetical protein
ECA3672	4121650	4122130	YP_051760.1	-	hypothetical protein
ECA3673	4122178	4122517	YP_051761.1	-	hypothetical protein
ECA3674	4122636	4123044	YP_051762.1	-	hypothetical protein
ECA3675	4123036	4123897	YP_051763.1	-	hypothetical protein
Ddal137					
=======					
ECA3695	4144590	4144983	YP_051783.1	-	phage regulatory protein protein
ECA3696	4144979	4145402	YP_051784.1	-	putative phage regluatory protein
ECA3697	4145379	4145580	YP_051785.1	-	hypothetical protein
ECA3698	4145576	4146017	YP_051786.1	-	hypothetical protein
ECA3699	4146027	4146327	YP_051787.1	-	conserved hypothetical phage-related protein
ECA3700	4146417	4147026	YP_051788.1	-	conserved hypothetical phage-related protein
ECA3701	4147038	4147308	YP_051789.1	-	conserved hypothetical phage-related protein
ECA3702	4147335	4147593	YP_051790.1	-	hypothetical phage-related protein
ECA3703	4147595	4148747	YP_051791.1	-	conserved hypothetical phage-related protein
ECA3704	4148756	4150526	YP_051792.1	-	conserved hypothetical phage-related protein
ECA3705	4150535	4151444	YP_051793.1	-	conserved hypothetical phage-related protein
ECA3706	4151453	4151759	YP_051794.1	-	putative phage-related DNA-binding protein
ECA3707	4151811	4152000	YP_051795.1	-	putative phage-related DNA-binding protein
ECA3708	4152091	4152508	YP_051796.1	-	putative phage-related DNA-binding protein
ECA3709	4152526	4153063	YP_051797.1	-	putative phage-related membrane protein
ECA3710	4153097	4154042	YP_051798.1	-	putative phage-related lipoprotein
ECA3711	4154161	4155169	YP_051799.1	-	conserved hypothetical phage-related protein
ECA3712	4155171	4156353	YP_051800.1	-	putative phage-related membrane protein
ECA3713	4156500	4156845	YP_051801.1	-	putative phage-related membrane protein
Ddal138					
=======					
ECA3719	4159329	4160865	YP_051807.1	-	putative phage-related protein
ECA3720	4160864	4162358	YP_051808.1	-	putative phage-related protein
ECA3721	4162338	4163160	YP_051809.1	-	putative phage-related protein
ECA3722	4163156	4163606	YP_051810.1	-	putative phage-related protein
ECA3723	4163801	4164911	YP_051811.1	-	conserved phage-related protein
ECA3724	4164947	4165883	YP_051812.1	-	conserved phage-related protein
Ddal139					
=======					
ECA3730	4168785	4169310	YP_051818.1	-	major tail sheath protein
ECA3731	4169316	4169517	YP_051819.1	-	hypothetical protein

ECA3731A	4169657	4169960	YP_051820.1	-	putative phage-related protein
ECA3732	4170346	4172818	YP_051821.1	-	putative bacteriophage tail protein (Tape-measure)
ECA3733	4172817	4173702	YP_051822.1	-	putative phage-related protein
ECA3734	4173698	4173914	YP_051823.1	-	putative bacteriophage tail fibre protein
ECA3735	4173901	4175047	YP_051824.1	-	putative bacteriophage protein
ECA3736	4175043	4175637	YP_051825.1	-	putative bacteriophage baseplate protein
ECA3737	4175663	4176512	YP_051826.1	-	putative phage-related protein
DdaI140					
=======					
ECA3741	4178590	4180153	YP_051830.1	-	putative bacteriophage tail fiber protein
ECA3742	4180152	4180770	YP_051831.1	-	putative bacteriophage tail fiber assembly protein
ECA3743	4180821	4181157	YP_051832.1	-	hypothetical protein
DdaI141					
=======					
ECA3768	4209248	4211753	YP_051857.1	-	putative formate acetyltransferase
ECA3769	4211838	4212789	YP_051858.1	-	putative pyruvate formate-lyase activating enzyme
ECA3770	4213114	4213531	YP_051859.1	qacH	quaternary ammonium compound resistance protein
ECA3771	4213548	4213875	YP_051860.1	-	putative multidrug resistance protein
ECA3772	4214034	4215231	YP_051861.1	-	LacI-family transcriptional regulator
ECA3773	4215530	4215974	YP_051862.1	sgcA	putative phosphotransferase enzyme II, A component
ECA3774	4215996	4216284	YP_051863.1	-	hypothetical protein
DdaI142					
=======					
ECA3780	4222571	4222883	YP_051869.1	-	ArsR-family transcriptional regulator
ECA3781	4223013	4225257	YP_051870.1	fepA	TonB dependent siderophore receptor
DdaI143					
=======					
ECA3792	4239709	4241143	YP_051881.1	-	hypothetical protein
ECA3793	4241117	4242077	YP_051882.1	-	putatuve glycosysl hydrolase
DdaI144					
=======					
ECA3835	4286954	4287266	YP_051923.1	-	hypothetical protein
ECA3836	4287262	4287997	YP_051924.1	-	hypothetical protein
ECA3837	4288181	4289018	YP_051925.1	-	AraC-family transcriptional regulator
ECA3838	4289236	4290856	YP_051926.1	-	methyl-accepting chemotaxis protein
ECA3839	4290970	4291162	YP_051927.1	-	putative matrix protein
ECA3840	4291682	4292240	YP_051928.1	-	hypothetical protein
ECA3841	4292627	4294286	YP_051929.1	-	putative extracellular solute-binding protein
ECA3842	4294344	4295274	YP_051930.1	-	putative isoflavone oxidoreductase
DdaI145					
=======					
ECA3943	4413808	4415560	YP_052030.1	-	hypothetical protein
ECA3944	4415556	4415766	YP_052031.1	-	putative phage-related protein
ECA3945	4415813	4416323	YP_052032.1	-	hypothetical protein
ECA3946	4417002	4418481	YP_052033.1	-	hypothetical protein
ECA3947	4418703	4419630	YP_052034.1	-	putative D-isomer specific 2-hydroxyacid dehydrogenase
ECA3948	4419691	4420816	YP_052035.1	-	hypothetical protein
ECA3949	4420812	4421475	YP_052036.1	-	hypothetical protein
ECA3950	4421778	4422828	YP_052037.1	-	putative zinc-binding dehydrogenase
ECA3951	4423295	4423913	YP_052038.1	-	LysE-type translocator
ECA3952	4424159	4425050	YP_052039.1	-	LysR-family transcriptional regulator
ECA3953	4425069	4426236	YP_052040.1	-	probable transporter
ECA3954	4426300	4427116	YP_052041.1	-	ABC transporter, ATP-binding protein
ECA3955	4427115	4427880	YP_052042.1	-	ABC transporter protein, membrane protein
ECA3956	4427895	4428915	YP_052043.1	-	ABC transporter, substrate binding protein
DdaI146					
=======					
ECA3960	4431769	4432609	YP_052047.1	-	putative hydrolase
ECA3961	4432619	4433255	YP_052048.1	-	hypothetical protein
ECA3962	4433306	4433852	YP_052049.1	-	hypothetical protein
ECA3963	4433914	4434142	YP_052050.1	-	hypothetical protein
DdaI147					
=======					
ECA4045	4511593	4512688	YP_052132.1	pmrB	sensor protein BasS/PmrB
ECA4046	4512739	4514011	YP_052133.1	-	putative serine hydroxymethyltransferase
ECA4047	4514043	4514856	YP_052134.1	-	putative alanine racemase
ECA4048	4514959	4516456	YP_052135.1	-	GntR-family transcriptional regulator
DdaI148					
=======					
ECA4052	4518537	4518864	YP_052139.1	-	hypothetical protein
ECA4053	4518863	4519643	YP_052140.1	-	short chain dehydrogenase
DdaI149					
=======					
ECA4078	4546706	4546904	YP_052165.1	-	hypothetical protein
ECA4079	4546958	4549529	YP_052166.1	nirB	nitrite reductase [NAD(P)H] large subunit
ECA4080	4549525	4549894	YP_052167.1	nirD	nitrite reductase small subunit
ECA4081	4549961	4551341	YP_052168.1	cysG	siroheme synthase
ECA4082	4551833	4552562	YP_052169.1	occQ	octopine transport system permease protein
ECA4083	4552561	4553296	YP_052170.1	occM	octopine transport system permease protein
ECA4084	4553292	4554057	YP_052171.1	occP	octopine permease ATP-binding protein P
DdaI150					
=======					
ECA4095	4564709	4565087	YP_052182.1	-	hypothetical protein

ECA4096	4565083	4565671	YP_052183.1	-	hypothetical protein
ECA4097	4565663	4566233	YP_052184.1	-	hypothetical protein
DdaI151					
=======					
ECA4109	4579773	4580184	YP_052196.1	-	hypothetical protein
ECA4110	4580246	4581398	YP_052197.1	-	putative periplasmic substrate-binding protein
ECA4111	4581390	4582641	YP_052198.1	-	probable transporter
ECA4112	4582714	4583872	YP_052199.1	-	putative octopine/opine/tauropine dehydrogenase
ECA4113	4583882	4584899	YP_052200.1	-	putative cystathionine beta-synthase
ECA4114	4584912	4586316	YP_052201.1	-	putative lysine/ornithine decarboxylase
ECA4115	4586357	4588553	YP_052202.1	-	TonB-dependent siderophore receptor
ECA4116	4588635	4590537	YP_052203.1	-	putative siderophore biosynthesis protein
ECA4117	4590787	4591669	YP_052204.1	-	putative citrate lyase beta chain
ECA4118	4591737	4592217	YP_052205.1	greB	transcription elongation factor GreB
ECA4119	4592391	4594107	YP_052206.1	-	putative acyltransferase
DdaI152					
=======					
ECA4129	4607030	4607510	YP_052216.1	-	hypothetical protein
ECA4130	4607522	4609268	YP_052217.1	-	alkaline phosphatase
DdaI153					
=======					
ECA4142	4624415	4626443	YP_052229.1	-	putative RHS accessory genetic element
ECA4143	4626444	4627170	YP_052230.1	-	hypothetical protein
ECA4144	4627166	4627685	YP_052231.1	-	hypothetical protein
ECA4145	4627806	4628325	YP_052232.1	-	hypothetical protein
ECA4146	4628324	4630040	YP_052233.1	-	hypothetical protein
DdaI154					
=======					
ECA4219	4720643	4721531	YP_052306.1	-	LysR-family transcriptional regulator
ECA4220	4721632	4722259	YP_052307.1	-	hypothetical protein
DdaI155					
=======					
ECA4278	4792989	4797273	YP_052365.1	-	Rhs-family protein
ECA4279	4797273	4797585	YP_052366.1	-	hypothetical protein
ECA4280	4797652	4797877	YP_052367.1	-	hypothetical protein
ECA4281	4797947	4798346	YP_052368.1	-	putative phage regulatory protein
DdaI156					
=======					
ECA4286	4803224	4803392	YP_052373.1	-	hypothetical protein
ECA4287	4803539	4803905	YP_052374.1	-	hypothetical protein
ECA4288	4803905	4804217	YP_052375.1	-	hypothetical protein
ECA4289	4804284	4804509	YP_052376.1	-	hypothetical protein
ECA4290	4804579	4804978	YP_052377.1	-	putative phage regulatory protein
ECA4291	4805079	4807998	YP_052378.1	-	DnaG primase-like protein
ECA4292	4808057	4809110	YP_052379.1	xerC	site-specific tyrosine recombinase XerC
ECA4293	4810141	4810594	YP_052380.1	-	hypothetical protein
ECA4294	4810593	4811115	YP_052381.1	-	hypothetical protein
DdaI157					
=======					
ECA4323	4841928	4842345	YP_052410.1	-	hypothetical protein
ECA4324	4842346	4843042	YP_052411.1	-	hypothetical protein
ECA4325	4843158	4843920	YP_052412.1	-	DeoR-family trancriptional regulator
ECA4326	4843916	4844132	YP_052413.1	-	hypothetical protein
DdaI158					
=======					
ECA4355	4876118	4876685	YP_052442.1	dcrB	hypothetical protein
ECA4356	4877183	4879178	YP_052443.1	-	hypothetical protein
ECA4357	4879177	4879885	YP_052444.1	-	putative lipoprotein
DdaI159					
=======					
ECA4363	4886992	4888174	YP_052450.1	-	hypothetical protein
ECA4364	4888642	4890289	YP_052451.1	-	hypothetical protein
ECA4365	4890290	4890512	YP_052452.1	-	hypothetical protein
ECA4366	4890508	4891822	YP_052453.1	-	hypothetical protein
DdaI160					
=======					
ECA4380	4911486	4912836	YP_052465.1	-	probable sugar transporter
ECA4381	4913049	4913964	YP_052466.1	-	putative DNA-binding protein
ECA4382	4914104	4915127	YP_052467.1	-	putative zinc-binding dehydrogenase
ECA4383	4915140	4916586	YP_052468.1	uxaB2	altronate oxidoreductase
DdaI161					
=======					
ECA4388	4922194	4924099	YP_052473.1	-	PTS system, IIabc component
ECA4389	4924230	4925058	YP_052474.1	-	beta-glucoside operon antiterminator
DdaI162					
=======					
ECA4396	4934119	4934395	YP_052481.1	-	hypothetical protein
ECA4397	4934378	4935272	YP_052482.1	-	hypothetical protein
DdaI163					
=======					
ECA4426	4965492	4967043	YP_052511.1	-	putative extracellular solute-binding protein
ECA4427	4967185	4968082	YP_052512.1	-	LysR-family transcriptional regulator
ECA4428	4968227	4969043	YP_052513.1	-	predicted hydrolase

ECA4429	4969442	4969667	YP_052514.1	-	putative transposase
ECA4430	4969668	4970379	YP_052515.1	-	putative lipoprotein
ECA4431	4970378	4972373	YP_052516.1	-	hypothetical protein
ECA4432	4972973	4974416	YP_052517.1	bglA	6-phospho-beta-glucosidase
ECA4433	4974428	4974776	YP_052518.1	-	pts system, cellobiose-specific IIa component
ECA4434	4974765	4976079	YP_052519.1	-	PTS system, IIbc component
ECA4435	4976095	4976401	YP_052520.1	-	pts system, IIb component
ECA4436	4976614	4977652	YP_052521.1	-	LacI-family transcriptional regulator
DdaI164					
=======					
ECA4447	4989708	4990275	YP_052532.1	-	hypothetical protein
ECA4448	4990459	4991356	YP_052533.1	-	hypothetical protein
ECA4449	4991420	4992272	YP_052534.1	-	hypothetical protein
ECA4450	4992372	4992792	YP_052535.1	-	hypothetical protein
ECA4451	4992908	4993331	YP_052536.1	hmsS	putative hemin storage protein
ECA4452	4993334	4994663	YP_052537.1	hmsR	N-glycosyltransferase PgaC
ECA4453	4994659	4996675	YP_052538.1	hmsF	putative hemin storage lipoprotein
ECA4454	4996686	4999149	YP_052539.1	hmsH	predicted outer membrane protein
ECA4455	4999352	5000237	YP_052540.1	-	hypothetical protein
ECA4457	5001993	5003496	YP_052541.1	smvA	putative methyl viologen resistance protein
DdaI165					
=======					
ECA4501	5042192	5043548	YP_052584.1	-	putative AMP-binding enzyme
ECA4502	5043544	5044123	YP_052585.1	-	hypothetical protein
ECA4503	5044135	5044387	YP_052586.1	-	acyl carrier protein
ECA4504	5044404	5044671	YP_052587.1	-	putative acyl carrier protein
ECA4505	5044690	5045479	YP_052588.1	-	putative acyltransferase
ECA4506	5045471	5046200	YP_052589.1	-	hypothetical protein
