# Supplementary material for: Microarray Comparative Genomic Hybridisation Analysis Incorporating Genomic Organisation, and Application to Enterobacterial Plant Pathogens
Source: PLoS Comput Biol. 2009 Aug 21;5(8):e1000473. doi: 10.1371/journal.pcbi.1000473 (PMC2718846; doi:10.1371/journal.pcbi.1000473)
Supplement: Table S5 — 168 Pba1043 genomic islands predicted to have no orthologues in Dda3937, but to have orthologues in both Pcc193 and Pba1039, prefixed PectoI. (0.16 MB PDF) [file pcbi.1000473.s005.pdf]

Supplementary Table 5: 168 *Pba*1043 genomic islands predicted to have no orthologues in *Dda*3937, but to have orthologues in both *Pcc*193 and *Pba*1039, prefixed PectoI

PectoI1
=======

ECA0043	51399	53091	YP_048172.1	ligB	NAD-dependent DNA ligase LigB
ECA0044	53349	55035	YP_048173.1	-	phosphoethanolamine transferase
ECA0045	55764	56313	YP_048174.1	-	putative virulence-associated outer membrane protein

PectoI2
=======

ECA0063	79097	79385	YP_048192.1	-	hypothetical protein
ECA0064	79381	79633	YP_048193.1	-	hypothetical protein
ECA0065	79888	80794	YP_048194.1	-	hypothetical protein
ECA0066	80828	81584	YP_048195.1	dsbG	disulfide isomerase/thiol-disulfide oxidase
ECA0067	81804	82428	YP_048196.1	-	putative LysE family transporter
ECA0068	82474	83347	YP_048197.1	-	LysR-family transcriptional regulator

PectoI3
=======

ECA0077	91665	92868	YP_048205.1	-	hypothetical protein
ECA0078	92959	93922	YP_048206.2	tkrA	2-hydroxyacid dehydrogenase
ECA0079	93966	94173	YP_048207.1	-	hypothetical protein
ECA0080	94275	96228	YP_048208.1	-	methyl-accepting chemotaxis protein

PectoI4
=======

ECA0105	125688	126339	YP_048233.1	expI	N-acylhomoserine lactone synthesis protein
ECA0106	126322	127051	YP_048234.1	expR	quorum-sensing transcriptional regulator

PectoI5
=======

ECA0115	134971	136117	YP_048243.1	-	putative fatty acid desaturase
ECA0116	136116	137208	YP_048244.1	-	putative fatty acid desaturase

PectoI6
=======

ECA0120	141448	142765	YP_048248.1	-	hypothetical protein
ECA0121	142829	143192	YP_048249.1	-	hypothetical protein
ECA0122	143223	143610	YP_048250.1	-	hypothetical protein
ECA0123	143718	144270	YP_048251.1	-	putative lipoprotein
ECA0124	144321	144879	YP_048252.1	-	putative lipoprotein
ECA0125	144969	145197	YP_048253.1	-	hypothetical protein
ECA0126	145495	145804	YP_048254.1	-	hypothetical protein
ECA0127	145800	147051	YP_048255.1	-	hypothetical protein
ECA0128	147193	147496	YP_048256.1	-	hypothetical protein
ECA0129	148810	150391	YP_048257.1	-	putative deoxycytidylate deaminase

PectoI7
=======

ECA0149	169391	170366	YP_048277.1	-	putative sugar transferase
ECA0150	170374	170854	YP_048278.1	coaD	phosphopantetheine adenylyltransferase
ECA0151	170890	172168	YP_048279.1	kdtA	3-deoxy-D-manno-octulosonic-acid transferase
ECA0152	172395	173259	YP_048280.1	-	putative glycosyltransferase
ECA0153	173306	174074	YP_048281.1	kdtX	lipopolysaccharide core biosynthesis glycosyl transferase
ECA0154	174160	174907	YP_048282.1	waaX	putative beta1,4-galactosyltransferase
ECA0155	174977	175163	YP_048283.1	-	hypothetical protein
ECA0156	175282	176065	YP_048284.1	-	hypothetical protein
ECA0157	176213	177230	YP_048285.1	waaJ	lipopolysaccharide 1,2-glucosyltransferase
ECA0158	177283	178294	YP_048286.1	waaI	lipopolysaccharide 1,3-galactosyltransferase
ECA0159	178360	179479	YP_048287.1	waaG	lipopolysaccharide core biosynthesis protein
ECA0160	179475	180546	YP_048288.1	waaQ	lipopolysaccharide core biosynthesis protein
ECA0161	180542	181769	YP_048289.1	waaL2	O-antigen ligase
ECA0162	182260	183544	YP_048290.1	waaL1	O-antigen ligase
ECA0163	183540	184524	YP_048291.1	waaC	ADP-heptose:LPS heptosyl transferase I

PectoI8
=======

ECA0178	197366	197699	YP_048305.1	-	hypothetical protein

PectoI9
=======

ECA0182	202589	204218	YP_048309.1	-	methyl-accepting chemotaxis protein
ECA0183	204905	206567	YP_048310.1	-	methyl-accepting chemotaxis protein

PectoI10
========

ECA0189	214456	215656	YP_048316.1	-	putative mandelate racemase / muconate lactonizing enzyme
ECA0190	215661	216387	YP_048317.1	-	GntR-family transcriptional regulator

PectoI11
========

ECA0225	264270	265668	YP_048352.1	shiA	shikimate transporter
ECA0226	265738	265930	YP_048353.1	-	hypothetical protein
ECA0227	266103	267540	YP_048354.1	-	putative GntR-family transcriptional regulator

PectoI12
========

ECA0249	294648	295059	YP_048376.1	-	hypothetical protein
ECA0250	295275	296334	YP_048377.1	-	putative oxidoreductase
ECA0251	296588	297767	YP_048378.1	-	endonuclease/Exonuclease/phosphatase family protein
ECA0252	297835	298240	YP_048379.1	-	hypothetical protein
ECA0253	298565	299036	YP_048380.1	doc	putative death on curing protein

ECA0254	299035	299440	YP_048381.1	-	hypothetical protein
PectoI13					
========					
ECA0338	386070	386934	YP_048464.1	-	hypothetical protein
ECA0339	387023	387875	YP_048465.1	-	probable sugar-bisphosphate aldolase
ECA0340	387886	388978	YP_048466.1	-	PTS system, IIbc component
ECA0341	389003	389318	YP_048467.1	-	PTS system, IIb component
ECA0342	389331	389811	YP_048468.1	-	PTS system, EIIa component
ECA0343	389859	391410	YP_048469.1	-	probable PTS system regulatory protein
ECA0344	391921	392257	YP_048470.1	-	hypothetical protein
PectoI14					
========					
ECA0365	417454	418135	YP_048491.1	-	hypothetical protein
ECA0366	418294	418606	YP_048492.1	-	hypothetical protein
ECA0367	418602	419019	YP_048493.1	-	putative DNA-binding protein
PectoI15					
========					
ECA0407	466209	466377	YP_048532.1	-	putative integrase (partial)
ECA0408	466557	466815	YP_048533.1	-	putative plasmid-related protein
PectoI16					
========					
ECA0414	473424	473673	YP_048537.1	-	putative bacteriophage regulatory protein
PectoI17					
========					
ECA0425	481913	483335	YP_048543.1	-	putative outer membrane efflux protein
ECA0426	483704	485144	YP_048544.1	aspA1	aspartate ammonia-lyase
ECA0427	485397	486354	YP_048545.1	-	LysR-family transcriptional regulator
ECA0428	487121	488642	YP_048546.1	-	putative porin
ECA0429	488658	490308	YP_048547.1	-	hypothetical protein
ECA0430	490304	491135	YP_048548.1	-	hypothetical protein
ECA0431	491594	492941	YP_048549.1	-	hypothetical protein
ECA0432	492952	493744	YP_048550.1	-	hypothetical protein
ECA0433	493896	494994	YP_048551.1	-	putative lipoprotein
ECA0434	495148	497098	YP_048552.1	-	methyl-accepting chemotaxis protein
ECA0435	497560	500272	YP_048553.1	mgtB	Magnesium transport ATPase
ECA0436	500718	502296	YP_048554.1	-	methyl-accepting chemotaxis protein
PectoI18					
========					
ECA0443	508847	509102	YP_048561.1	-	hypothetical protein
ECA0444	509098	509350	YP_048562.1	-	antitoxin YefM
PectoI19					
========					
ECA0452	518033	519071	YP_048570.1	-	putative zinc-binding dehydrogenase
ECA0454	519484	519781	YP_048572.1	-	hypothetical protein
ECA0455	519799	520555	YP_048573.1	-	hypothetical protein
ECA0456	520561	521041	YP_048574.1	-	hypothetical protein
PectoI20					
========					
ECA0467	532187	533090	YP_048585.1	-	hypothetical protein
ECA0468	533381	534971	YP_048586.1	prfC	peptide chain release factor 3
ECA0469	535472	536081	YP_048587.1	osmY	periplasmic protein
PectoI21					
========					
ECA0473	537937	539215	YP_048591.1	-	putative Na+ dependent nucleoside transporter-family protein
ECA0474	539364	540081	YP_048592.1	entD	enterobactin synthetase component D (4'-phosphopantetheinyl
transferase)					
ECA0475	540115	542077	YP_048593.1	-	TonB-dependent siderophore receptor protein
PectoI22					
========					
ECA0485	560282	562364	YP_048603.1	-	hypothetical protein
ECA0486	562386	562722	YP_048604.1	-	hypothetical protein
PectoI23					
========					
ECA0492	567395	568499	YP_048610.1	phnI	putative phosphonate metabolism protein
PectoI24					
========					
ECA0497	572076	572643	YP_048615.1	phnN	ribose 1,5-bisphosphokinase
ECA0498	572684	573470	YP_048616.1	phnP	carbon-phosphorus lyase complex accessory protein
PectoI25					
========					
ECA0516	590843	591731	YP_048634.1	-	hypothetical protein
ECA0517	591723	592413	YP_048635.1	-	hypothetical protein
ECA0518	592409	592802	YP_048636.1	-	hypothetical protein
ECA0519	592767	594162	YP_048637.1	-	replicative DNA helicase
ECA0520	594161	595886	YP_048638.1	-	hypothetical protein
ECA0521	595889	596162	YP_048639.1	-	hypothetical protein
ECA0522	596186	596801	YP_048640.1	-	hypothetical protein
ECA0522A	596809	597064	YP_048641.1	-	hypothetical protein
ECA0523	597236	598433	YP_048642.1	-	hypothetical protein
ECA0524	598802	599600	YP_048643.1	-	hypothetical protein
ECA0525	599596	601615	YP_048644.1	topB	DNA topoisomerase III
ECA0526	601918	602095	YP_048645.1	-	hypothetical protein
ECA0527	602137	602293	YP_048646.1	-	hypothetical protein

ECA0528	602463	602955	YP_048647.1	-	hypothetical protein
ECA0531	603653	604091	YP_048648.1	-	putative plasmid-related protein
ECA0532	604236	605553	YP_048649.1	pilL	putative Type IV pilus protein
ECA0533	605552	605999	YP_048650.1	pilM	putative Type IV pilus protein
ECA0534	606012	607677	YP_048651.1	pilN	putative Type IV pilus protein
ECA0537	608989	609487	YP_048652.1	pilP	putative Type IV pilus protein
ECA0538	609486	611046	YP_048653.1	pilQ	putative Type IV pilus nucleotide-binding protein
ECA0539	611014	611167	YP_048654.1	-	hypothetical protein
ECA0540	611177	612215	YP_048655.1	pilR	putative Type IV pilus protein
ECA0541	612270	612876	YP_048656.1	pilS	putative type IV pilus prepilin
ECA0542	612882	613401	YP_048657.1	pilT	putative Type IV pilus protein
ECA0543	613397	614057	YP_048658.1	pilU	putative prepilin peptidase
ECA0544	614070	615465	YP_048659.1	pilV	putative Type IV pilus prepilin protein
ECA0545	615468	615948	YP_048660.1	-	alternative C-terminus for the PilV protein (fragment)
ECA0546	616234	617362	YP_048662.1	rci	shufflon-specific DNA recombinase
ECA0547	617410	617902	YP_048663.1	-	hypothetical protein
ECA0548	618089	618890	YP_048664.1	traE	putative plasmid transfer protein
ECA0549	619033	620236	YP_048665.1	traF	putative plasmid transfer protein
ECA0550	620302	620752	YP_048666.1	-	putative plasmid protein protein
ECA0551	620818	621118	YP_048667.1	-	hypothetical protein
ECA0552	621155	621848	YP_048668.1	-	hypothetical protein
ECA0553	621945	622320	YP_048669.1	-	hypothetical protein
ECA0554	622422	623298	YP_048670.1	-	hypothetical protein
ECA0555	623307	624021	YP_048671.1	-	hypothetical protein
ECA0556	624020	624608	YP_048672.1	-	hypothetical protein
ECA0557	624608	625130	YP_048673.1	-	hypothetical protein
ECA0558	625126	625744	YP_048674.1	-	hypothetical protein
ECA0559	625721	626204	YP_048675.1	-	hypothetical protein
ECA0560	626207	628304	YP_048676.1	-	putative plasmid transfer protein
ECA0561	628305	629061	YP_048677.1	-	hypothetical protein
ECA0562	629083	629278	YP_048678.1	-	hypothetical protein
ECA0563	629419	629959	YP_048679.1	-	hypothetical protein
ECA0564	630093	630414	YP_048680.1	-	hypothetical protein
ECA0565	630415	630655	YP_048681.1	-	hypothetical protein
ECA0566	630681	631023	YP_048682.1	-	hypothetical protein
ECA0567	631032	631395	YP_048683.1	-	hypothetical protein
ECA0568	631391	632048	YP_048684.1	-	hypothetical protein
ECA0569	632044	632971	YP_048685.1	-	hypothetical protein
ECA0570	632960	634493	YP_048686.1	-	hypothetical protein
ECA0571	634610	634946	YP_048687.1	-	hypothetical protein
ECA0572	634938	635349	YP_048688.1	-	putative lipoprotein
ECA0573	635348	638210	YP_048689.1	-	putative plasmid-related protein
ECA0574	638206	638653	YP_048690.1	-	hypothetical protein
ECA0575	638754	639189	YP_048691.1	-	hypothetical protein
ECA0576	639328	639733	YP_048692.1	-	hypothetical protein
ECA0577	639725	640700	YP_048693.1	-	hypothetical protein
ECA0578	640709	642134	YP_048694.1	-	hypothetical protein
ECA0579	642136	642508	YP_048695.1	-	hypothetical protein
ECA0580	642504	644037	YP_048696.1	-	hypothetical protein
ECA0581	644069	644459	YP_048697.1	-	hypothetical protein
ECA0582	644601	644961	YP_048698.1	-	putative plasmid-related protein

Pectol26
========

ECA0586	648537	648678	YP_048702.1	-	hypothetical protein
ECA0586A	648730	648898	YP_048703.1	-	hypothetical protein
ECA0587	649513	650404	YP_048704.1	-	hypothetical protein
ECA0588	650411	650702	YP_048705.1	-	putative plasmid-related protein
ECA0589	651096	651408	YP_048706.1	-	hypothetical protein
ECA0590	651457	651814	YP_048707.1	-	hypothetical protein
ECA0591	651895	652369	YP_048708.1	-	hypothetical protein
ECA0592	652457	652862	YP_048709.1	-	hypothetical protein
ECA0593	653072	653486	YP_048710.1	-	putative DNA repair protein
ECA0594	653662	654259	YP_048711.1	-	hypothetical protein
ECA0595	654255	654528	YP_048712.1	-	hypothetical protein
ECA0596	654598	655084	YP_048713.1	-	hypothetical protein
ECA0597	655149	657111	YP_048714.1	-	hypothetical protein
ECA0598	657185	658079	YP_048715.1	-	hypothetical protein
ECA0599	658153	659074	YP_048716.1	-	hypothetical protein

Pectol27
========

ECA0611	683331	684792	YP_048728.1	-	hypothetical protein
ECA0612	684911	685403	YP_048729.1	-	hypothetical protein
ECA0613	685695	687294	YP_048730.1	-	hypothetical protein
ECA0614	687379	688402	YP_048731.1	-	putative phage integrase
ECA0615	688831	689677	YP_048732.1	-	AraC-family transcriptional regulator

Pectol28
========

ECA0640	710755	711145	YP_048756.1	-	hypothetical protein
ECA0641	711150	711546	YP_048757.1	-	hypothetical protein

Pectol29
========

ECA0658	731958	732447	YP_048773.1	-	periplasmic protein
ECA0659	732645	734115	YP_048774.1	-	putative signaling membrane protein

PectoI30
========

ECA0705	774295	774733	YP_048819.1	-	putative degenerate non-ribosomal peptide synthetase (partial)
ECA0706	774825	775116	YP_048820.1	-	putative degenerate non-ribosomal peptide synthetase (partial)
ECA0707A	775622	775718	YP_048821.1	-	hypothetical protein (partial)
ECA0708	776321	776672	YP_048822.1	evr	virulence regulator

PectoI31
========

ECA0731	799577	801830	YP_048843.1	fcuA	putative ferric siderophore TonB-dependent receptor
ECA0732	801919	802399	YP_048844.1	-	hypothetical protein

PectoI32
========

ECA0762	834357	834681	YP_048874.1	-	putative lipoprotein
ECA0763	834709	835228	YP_048875.1	fldB	flavodoxin FldB
ECA0764	835455	835632	YP_048876.1	-	hypothetical protein
ECA0765	835646	836093	YP_048877.1	-	hypothetical protein
ECA0766	836293	836902	YP_048878.1	-	hypothetical protein
ECA0767	837168	837669	YP_048879.1	-	hypothetical protein
ECA0768	837919	838096	YP_048880.1	-	hypothetical protein

PectoI33
========

ECA0779	851059	851452	YP_048891.1	-	hypothetical protein
ECA0780	851562	852633	YP_048892.1	-	hypothetical protein

PectoI34
========

ECA0785	858629	859340	YP_048897.1	-	two component system response regulator
ECA0786	859317	860817	YP_048898.1	-	two component system sensor kinase
ECA0787	861359	861911	YP_048899.1	-	hypothetical protein
ECA0788	861959	862904	YP_048900.1	-	hypothetical protein

PectoI35
========

ECA0797	871251	871884	YP_048909.1	-	hypothetical protein
ECA0798	872063	873695	YP_048910.1	-	hypothetical protein
ECA0799	873753	883440	YP_048911.1	-	putative outer membrane protein

PectoI36
========

ECA0804	890039	891770	YP_048916.1	rhiE	rhamnogalacturonate lyase
ECA0805	891898	892831	YP_048917.1	-	hypothetical protein

PectoI37
========

ECA0818	907055	907505	YP_048928.1	-	hypothetical protein
ECA0819	907497	908973	YP_048929.1	uxuB	putative D-mannonate oxidoreductase
ECA0820	909307	910588	YP_048930.1	-	O-acetyl-L-homoserine sulfhydrylase
ECA0821	910650	911748	YP_048931.1	-	hypothetical protein
ECA0822	911923	912721	YP_048932.1	-	putative carbon-nitrogen hydrolase
ECA0823	912742	913279	YP_048933.1	cybB	cytochrome B561
ECA0824	913552	917086	YP_048934.1	-	pyruvate-flavodoxin oxidoreductase
ECA0825	917475	917901	YP_048935.1	-	hypothetical protein
ECA0826	918245	918758	YP_048936.1	-	putative GNAT-family acetyltransferase
ECA0827	918801	919428	YP_048937.1	-	putative amino acid efflux protein
ECA0828	919624	920407	YP_048938.1	-	putative amino acid-binding periplasmic protein
ECA0829	920664	921243	YP_048939.1	-	hypothetical protein

PectoI38
========

ECA0835	925540	926821	YP_048945.1	-	prophage integrase

PectoI39
========

ECA0849	939858	940740	YP_048959.1	-	putative sugar ABC transporter, permease protein
ECA0850	940741	941554	YP_048960.1	-	putative sugar ABC transporter, permease protein
ECA0851	941590	942676	YP_048961.1	-	putative sugar ABC transporter ATP-binding protein
ECA0852	943024	944548	YP_048962.1	-	putative exported plant proteoglycan hydrolase

PectoI40
========

ECA0856	947608	948505	YP_048966.1	-	ABC transporter, periplasmic binding protein
ECA0858	948917	949769	YP_048967.1	-	putative beta-glucoside operon antiterminator

PectoI41
========

ECA0863	956802	957261	YP_048972.1	rpiB	ribose-5-phosphate isomerase B
ECA0864	957604	957808	YP_048973.1	-	hypothetical protein

PectoI42
========

ECA0876	971014	973210	YP_048985.1	iutA	TonB-dependent ferric aerobactin receptor
ECA0877	973974	974826	YP_048986.1	-	TonB-like protein
ECA0878	974923	977506	YP_048987.1	-	hypothetical protein
ECA0879	977611	980386	YP_048988.1	-	putative zinc protease
ECA0880	980391	982056	YP_048989.1	-	putative ABC transporter ATP-binding protein

PectoI43
========

ECA0921	1029049	1029736	YP_049030.1	-	GntR-family transcriptional regulator

PectoI44
========

ECA0935	1046526	1048740	YP_049044.1	fhuE	ferric-rhodotorulic acid outer membrane transporter
ECA0936	1048832	1050182	YP_049045.1	-	putative glucarate dehydratase

ECA0937 1050174 1051521 YP_049046.1 - putative transport protein
ECA0938 1051811 1052303 YP_049047.1 - hypothetical protein
ECA0939 1052369 1052939 YP_049048.1 - putative fimbrial protein
ECA0940 1052966 1054001 YP_049049.1 - hypothetical protein
ECA0941 1054019 1054769 YP_049050.1 - putative fimbrial chaperone
ECA0942 1054807 1057300 YP_049051.1 - putative outer membrane usher protein
ECA0943 1057441 1057990 YP_049052.1 - fimbriae major subunit protein
ECA0944 1058635 1059439 YP_049053.1 - hypothetical protein
ECA0945 1059425 1059896 YP_049054.1 - hypothetical protein
ECA0946 1059957 1060530 YP_049055.1 - TetR-family transcriptional regulator
ECA0947 1060622 1061516 YP_049056.1 - hypothetical protein
ECA0948 1061621 1062176 YP_049057.1 - hypothetical protein
ECA0949 1062409 1063174 YP_049058.1 - hypothetical protein
ECA0950 1063489 1064089 YP_049059.1 - TetR-family transcriptional regulator
ECA0951 1064206 1064794 YP_049060.1 - probable NAD(P)H oxidoreductase
ECA0952 1064819 1064996 YP_049061.1 - hypothetical protein
ECA0953 1065059 1065287 YP_049062.1 - hypothetical protein
PectoI45
========
ECA0970 1080116 1081325 YP_049077.1 - hypothetical protein
ECA0971 1081338 1082172 YP_049078.1 - hypothetical protein
ECA0972 1082386 1083430 YP_049079.1 - hypothetical protein
ECA0973 1083392 1084556 YP_049080.1 - AraC-family transcriptional regulator
PectoI46
========
ECA1002 1122905 1123823 YP_049109.1 - putative dihydrodipicolinate synthase
ECA1003 1123896 1124589 YP_049110.1 - putative tetR-family transcription factor
PectoI47
========
ECA1052 1177367 1178066 YP_049159.1 cutF lipoprotein involved with copper homeostasis and adhesion
ECA1053 1178138 1180703 YP_049160.1 mutS DNA mismatch repair protein
PectoI48
========
ECA1069 1198649 1199129 YP_049176.1 - hypothetical protein
ECA1070 1199329 1200754 YP_049177.1 - putative 3-polyprenyl-4-hydroxybenzoate carboxy-lyase
PectoI49
========
ECA1079 1209125 1209647 YP_049186.1 fecI RNA polymerase sigma factor FecI
ECA1080 1209692 1209875 YP_049187.1 - hypothetical protein
ECA1081 1209939 1211562 YP_049188.1 - probable transcriptional regulator
PectoI50
========
ECA1089 1217226 1218471 YP_049196.1 - hypothetical protein
ECA1090 1218825 1220019 YP_049197.1 - putative lipoprotein
ECA1092 1220230 1220965 YP_049198.1 - GntR-family transcriptional regulator
ECA1093 1221232 1222429 YP_049199.1 uxuA mannonate dehydratase
ECA1094 1222650 1223694 YP_049200.1 pelI pectate lyase
ECA1095 1224543 1225752 YP_049201.1 pehA endo-polygalacturonase
ECA1096 1225994 1227164 YP_049202.1 - putative type I secretion protein
ECA1097 1227175 1229320 YP_049203.1 - putative type I secretion protein, ATP-binding protein
ECA1098 1229353 1230796 YP_049204.1 - putative type I secretion protein
ECA1099 1231300 1242148 YP_049205.1 - large repetitive protein
ECA1100 1242165 1245936 YP_049206.1 - hypothetical protein
ECA1101 1246645 1248292 YP_049207.1 - multidrug transporter membrane component/ATP-binding
component
ECA1102 1248507 1249548 YP_049208.1 ansB1 L-asparaginase precursor
ECA1103 1250101 1250623 YP_049209.1 aroL shikimate kinase II
ECA1104 1250761 1251046 YP_049210.1 - hypothetical protein
ECA1105 1251538 1253329 YP_049211.1 - putative methyl-accepting chemotaxis protein
ECA1106 1253390 1254320 YP_049212.1 rdgC recombination associated protein
ECA1107 1254671 1255583 YP_049213.1 - fructokinase
PectoI51
========
ECA1183 1339633 1339852 YP_049289.1 - hypothetical protein
ECA1184 1339958 1341608 YP_049290.1 ushA bifunctional UDP-sugar hydrolase/5'-nucleotidase periplasmic
precursor
ECA1185 1341796 1343851 YP_049291.1 - hypothetical protein
ECA1186 1343980 1346041 YP_049292.1 - hypothetical protein
PectoI52
========
ECA1191 1351948 1352428 YP_049297.1 - hypothetical protein
ECA1192 1352498 1353317 YP_049298.1 - hypothetical protein
PectoI53
========
ECA1204 1369799 1372484 YP_049310.1 - phosphotransfer intermediate protein in two-component
regulatory system with RcsBC
ECA1205 1372497 1373667 YP_049311.1 - hypothetical protein
ECA1206 1373803 1374223 YP_049312.1 - probable transcriptional regulator
ECA1207 1374600 1375371 YP_049313.1 - probable short chain dehydrogenase
ECA1208 1375436 1376318 YP_049314.1 - probable short chain dehydrogenase
PectoI54
========
ECA1248 1419552 1419789 YP_049354.1 - hypothetical protein

ECA1249	1419999	1420545	YP_049355.1	hydN	electron transport protein HydN
ECA1250	1420601	1422752	YP_049356.1	fdhF	formate dehydrogenase H
ECA1251	1422748	1425052	YP_049357.1	hypF	hydrogenase maturation protein
PectoI55					
========					
ECA1274	1448116	1448638	YP_049380.1	-	RNA polymerase sigma factor
ECA1275	1449047	1451423	YP_049381.1	-	TonB-dependent ferrichrome-iron receptor
ECA1276	1451497	1453288	YP_049382.1	-	ABC transporter ATP binding component
ECA1277	1453349	1453721	YP_049383.1	-	hypothetical protein
PectoI56					
========					
ECA1281	1455487	1457158	YP_049387.1	-	methyl-accepting chemotaxis protein
ECA1282	1457256	1458435	YP_049388.1	atoB	acetyl-CoA acetyltransferase
ECA1283	1458446	1459094	YP_049389.1	atoA	acetate CoA-transferase beta subunit
ECA1284	1459093	1459750	YP_049390.1	atoD	acetyl-CoA:acetoacetyl-CoA transferase subunit alpha
ECA1285	1459906	1460206	YP_049391.1	-	hypothetical protein
ECA1286	1460205	1461546	YP_049392.1	-	hypothetical protein
ECA1287	1461607	1462930	YP_049393.1	citM	Mg(2+)/citrate complex transporter
ECA1288	1463403	1464303	YP_049394.1	-	LysR-family transcriptional regulator
PectoI57					
========					
ECA1321	1495195	1495876	YP_049427.1	-	hypothetical protein
ECA1322	1495946	1496435	YP_049428.1	-	hypothetical protein
PectoI58					
========					
ECA1344	1523467	1523674	YP_049450.1	-	hypothetical protein
ECA1345	1523817	1525473	YP_049451.1	-	ABC transporter permease protein
ECA1346	1525673	1526654	YP_049452.1	-	ABC transporter substrate binding protein
ECA1347	1526671	1527400	YP_049453.1	-	ABC transporter ATP-binding protein
PectoI59					
========					
ECA1417	1607968	1609105	YP_049523.1	wza	putative polysaccharide export protein
ECA1418	1609113	1609548	YP_049524.1	wzb	probable protein-tyrosine-phosphatase
ECA1419	1609564	1611733	YP_049525.1	wzc	tyrosine kinase
PectoI60					
========					
ECA1466	1666598	1666913	YP_049572.1	-	hypothetical protein
ECA1467	1666912	1667191	YP_049573.1	-	hypothetical protein
PectoI61					
========					
ECA1485	1680732	1681461	YP_049590.1	-	putative lipoprotein
ECA1486	1681841	1683506	YP_049591.1	-	ABC transporter ATP-binding protein
ECA1487	1683592	1704739	YP_049592.1	-	non-ribosomal peptide synthetase
ECA1488	1704731	1727303	YP_049593.1	-	non-ribosomal peptide synthetase
ECA1489	1728128	1729391	YP_049594.1	lacY	galactoside permease
ECA1490	1729533	1732656	YP_049595.1	lacZ	beta-D-galactosidase
PectoI62					
========					
ECA1509	1754694	1756374	YP_049611.1	-	methyl-accepting chemotaxis protein
ECA1510	1756919	1758824	YP_049612.1	-	putative iron permease
PectoI63					
========					
ECA1574	1830585	1831821	YP_049675.1	-	probable transporter
ECA1575	1831875	1832349	YP_049676.1	-	MerR-family transcriptional regulator
ECA1576	1832391	1832559	YP_049677.1	-	hypothetical protein
ECA1577	1832681	1833305	YP_049678.1	-	putative lipoprotein
ECA1578	1833670	1834210	YP_049679.1	-	hypothetical protein
ECA1579	1834369	1835260	YP_049680.1	-	2-dehydropantoate 2-reductase
ECA1580	1835449	1835932	YP_049681.1	-	putative cold-shock protein
ECA1581	1836064	1836310	YP_049682.1	-	hypothetical protein
ECA1582	1836536	1836671	YP_049683.1	-	putative transposase (partial)
ECA1583	1836750	1837002	YP_049684.1	-	putative integrase
PectoI64					
========					
ECA1679	1925681	1926959	YP_049780.1	intB	phage integrase
ECA1680	1927324	1928122	YP_049781.1	-	hypothetical protein
ECA1681	1928678	1930007	YP_049782.1	-	hypothetical protein
PectoI65					
========					
ECA1704	1951838	1952513	YP_049805.1	flgD	flagellar basal body rod modification protein
ECA1705	1952588	1953806	YP_049806.1	flgE	flagellar hook protein FlgE
PectoI66					
========					
ECA1710	1957412	1958375	YP_049811.1	flgJ	peptidoglycan hydrolase
ECA1711	1958501	1960208	YP_049812.1	flgK	flagellar hook-associated protein FlgK
ECA1712	1960239	1961193	YP_049813.1	flgL	flagellar hook-associated protein FlgL
PectoI67					
========					
ECA1729	1973614	1974025	YP_049829.1	fliS	flagellar protein FliS
ECA1730	1974064	1975483	YP_049830.1	fliD	flagellar capping protein
ECA1731	1975727	1976600	YP_049831.1	fliC	flagellin
PectoI68					
========					

ECA1762 2010924 2011254 YP_049862.1 - hypothetical protein
ECA1763 2011394 2012054 YP_049863.1 - hypothetical protein
ECA1764 2012811 2013066 YP_049864.1 - hypothetical protein
ECA1765 2013099 2013261 YP_049865.1 - hypothetical protein
ECA1766 2013547 2013970 YP_049866.1 - hypothetical protein
ECA1767 2013993 2014578 YP_049867.1 - Isochorismatase family protein
ECA1768 2014582 2015038 YP_049868.1 - putative acetyltransferase
ECA1769 2015202 2015631 YP_049869.1 - hypothetical protein
ECA1770 2015679 2016186 YP_049870.1 - hypothetical protein
ECA1771 2016269 2016455 YP_049871.1 - hypothetical protein
ECA1772 2016610 2016910 YP_049872.1 - hypothetical protein
ECA1773 2017068 2017350 YP_049873.1 - hypothetical protein
ECA1774 2018183 2019848 YP_049874.1 - methyl-accepting chemotaxis protein
PectoI69
========
ECA1783 2030936 2031491 YP_049883.1 - hypothetical protein
ECA1784 2031946 2032201 YP_049884.1 bssS biofilm formation regulatory protein BssS
ECA1785 2032437 2032695 YP_049885.1 - hypothetical protein
ECA1786 2032783 2033023 YP_049886.1 dinI DNA damage-inducible protein I
PectoI70
========
ECA1828 2075554 2075833 YP_049925.1 - hypothetical protein
ECA1829 2076004 2076721 YP_049926.1 - putative Crp/Fnr-family of transcriptional regulator
PectoI71
========
ECA1845 2091755 2092574 YP_049942.1 hmuT hemin-binding periplasmic protein
ECA1846 2092570 2093620 YP_049943.1 hemS hemin transport protein
ECA1847 2093826 2095149 YP_049944.1 aglB 6-phospho-alpha-glucosidase
PectoI72
========
ECA1872 2127145 2128786 YP_049969.1 - putative porin
ECA1873 2128872 2130063 YP_049970.1 - putative glycosyl hydrolase exoenzyme
ECA1874 2130167 2131181 YP_049971.1 - putative inosine-uridine preferring nucleoside hydrolase
ECA1875 2131620 2133054 YP_049972.1 nrfA cytochrome c nitrite reductase
PectoI73
========
ECA1900 2152116 2153838 YP_049997.1 narQ nitrate/nitrite sensor protein NarQ
ECA1901 2153925 2154558 YP_049998.1 narP DNA-binding response regulator in two-component regulatory system with NarQ or NarX
ECA1902 2154855 2155701 YP_049999.1 - AraC-family transcriptional regulator
ECA1903 2155749 2157117 YP_050000.1 - hypothetical protein
ECA1904 2157265 2158261 YP_050001.1 - probable zinc-binding dehydrogenase
PectoI74
========
ECA1970 2237015 2237987 YP_050065.1 - putative thiamine biosynthesis protein
ECA1971 2238072 2238627 YP_050066.1 aidA hypothetical protein
PectoI75
========
ECA1996 2264210 2264807 YP_050090.1 col colicin
ECA1997 2264868 2265129 YP_050091.1 imm colicin immunity protein
ECA1998 2265485 2265758 YP_050092.1 - hypothetical protein
ECA1999 2266316 2266607 YP_050093.1 - putative lipoprotein
PectoI76
========
ECA2007 2279510 2280350 YP_050101.1 - putative protease
ECA2008 2280395 2282174 YP_050102.1 - putative signaling protein
ECA2009 2282646 2283504 YP_050103.1 - putative exported protease
ECA2010 2283755 2284052 YP_050104.1 - hypothetical protein
PectoI77
========
ECA2021 2297112 2298807 YP_050115.1 - putative sulfate transporter YchM
ECA2022 2298970 2300071 YP_050116.1 chaA calcium/sodium:proton antiporter
ECA2022A 2300085 2300208 YP_050117.1 - putative surface antigen protein
ECA2023 2300400 2301204 YP_050118.1 - putative siderophore-interacting protein
PectoI78
========
ECA2042 2323395 2323677 YP_050137.1 - hypothetical protein
ECA2043 2323654 2324143 YP_050138.1 - hypothetical protein
ECA2044 2324295 2324544 YP_050139.1 - hypothetical protein
ECA2045 2324721 2325750 YP_050140.1 - putative NAD dependent epimerase/dehydratase
PectoI79
========
ECA2061 2346978 2348505 YP_050156.1 - methyl-accepting chemotaxis protein
ECA2062 2348944 2350252 YP_050157.1 - putative phosphatase
PectoI80
========
ECA2067 2354961 2355252 YP_050162.1 bor lipoprotein
PectoI81
========
ECA2090 2379835 2380900 YP_050185.1 hrpS sigma-54-dependent enhancer-binding protein
ECA2091 2380996 2382616 YP_050186.1 - putative signaling membrane protein
PectoI82
========

ECA2111 2398202 2398526 YP_050206.1 - potential HrpW-specific chaperone
ECA2112 2398527 2399967 YP_050207.1 hrpW type III effector protein
ECA2113 2400143 2404988 YP_050208.1 dspE putative avirulence protein
ECA2114 2405022 2405445 YP_050209.1 dspF putative avirulence protein
ECA2115 2405454 2406120 YP_050210.1 - putative transcriptional regulator
ECA2116 2406457 2408131 YP_050211.1 hecB putative hemolysin activator protein
ECA2117 2408180 2411585 YP_050212.1 hecA1 hemolysin/hemagglutinin-like protein
ECA2118 2411692 2426503 YP_050213.1 hecA2 hemolysin/hemagglutinin-like protein HecA precursor
PectoI83
========
ECA2133 2433252 2433552 YP_050228.1 - hypothetical protein
ECA2134 2433583 2434705 YP_050229.1 - ABC transporter, substrate binding protein
ECA2135 2434873 2436580 YP_050230.1 - periplasmic pectate lyase
ECA2136 2436679 2437444 YP_050231.1 - hypothetical protein
ECA2137 2437562 2438381 YP_050232.1 - hypothetical protein
ECA2138 2438377 2438554 YP_050233.1 - hypothetical protein
ECA2139 2439080 2439257 YP_050234.1 - hypothetical protein
ECA2140 2439404 2440412 YP_050235.1 - putative oxidoreductase
ECA2141 2440592 2442395 YP_050236.1 - amidase
PectoI84
========
ECA2149 2452531 2452981 YP_050244.1 - hypothetical protein
ECA2150 2453336 2453636 YP_050245.1 - hypothetical protein
PectoI85
========
ECA2159 2461265 2461811 YP_050254.1 hpaC putative 4-hydroxyphenylacetate 3-monooxygenase, reductase
component
ECA2160 2462085 2463282 YP_050255.1 - putative peptidase
ECA2161 2463566 2464547 YP_050256.1 - putative luciferase-like monooxygenase
ECA2162 2464655 2465162 YP_050257.1 - hypothetical protein
ECA2163 2466051 2468013 YP_050258.1 - autotransporter
ECA2164 2468108 2469152 YP_050259.1 ascG asc operon repressor
PectoI86
========
ECA2169 2473903 2474320 YP_050264.1 - hypothetical protein
ECA2170 2474316 2475462 YP_050265.1 - hypothetical protein
ECA2171 2475467 2476352 YP_050266.1 - hypothetical protein
ECA2172 2476853 2477447 YP_050267.1 - hypothetical protein
ECA2173 2477691 2478756 YP_050268.1 - hypothetical protein
ECA2174 2479014 2479443 YP_050269.1 - hypothetical protein
PectoI87
========
ECA2208 2507443 2507977 YP_050301.1 ogt methylated-DNA--protein-cysteine methyltransferase
ECA2209 2508507 2509803 YP_050302.1 - putative oxidoreductase
ECA2210 2509911 2511231 YP_050303.1 - periplasmic binding protein precursor
ECA2211 2511297 2511822 YP_050304.1 - copper-zinc superoxide dismutase
ECA2212 2511875 2513105 YP_050305.1 - putative cyclopropane-fatty-acyl-phospholipid synthase
ECA2213 2513101 2513827 YP_050306.1 - hypothetical protein
ECA2214 2513819 2515088 YP_050307.1 - putative amine oxidoreductase
ECA2215 2515093 2515801 YP_050308.1 - putative short-chain dehydrogenase
ECA2216 2515806 2516286 YP_050309.1 - hypothetical protein
PectoI88
========
ECA2220 2523661 2524351 YP_050313.1 - putative cellulase
ECA2221 2524681 2525110 YP_050314.1 osmC osmotically inducible protein C
ECA2222 2525398 2525785 YP_050315.1 cybC putative soluble cytochrome b562
ECA2223 2525892 2526447 YP_050316.1 - TetR-family transcriptional regulator
ECA2224 2526582 2527779 YP_050317.1 - putative transporter
ECA2225 2527928 2528417 YP_050318.1 - putative acetyltransferase
ECA2225A 2528537 2528708 YP_050319.1 - hypothetical protein
ECA2226 2528724 2530560 YP_050320.1 - methyl-accepting chemotaxis protein
ECA2227 2530748 2531648 YP_050321.1 - chromosome replication initiation inhibitor protein
ECA2228 2531644 2531911 YP_050322.1 - hypothetical protein
ECA2229 2532269 2532398 YP_050323.1 - hypothetical protein
ECA2230 2532527 2533646 YP_050324.1 - putative NADH-dependent flavin oxidoreductase
ECA2231 2533658 2534552 YP_050325.1 - hypothetical protein
PectoI89
========
ECA2237 2539269 2539632 YP_050332.1 flxA hypothetical protein
ECA2238 2539821 2540028 YP_050333.1 - hypothetical protein
ECA2239 2540217 2540505 YP_050334.1 - hypothetical protein
ECA2240 2540510 2541806 YP_050335.1 - putative lipoprotein
ECA2241 2541854 2542190 YP_050336.1 - hypothetical protein
ECA2242 2542220 2542454 YP_050337.1 - hypothetical protein
ECA2243 2542866 2544249 YP_050338.1 dat diaminobutyrate--2-oxoglutarate aminotransferase
ECA2244 2544267 2545785 YP_050339.1 ddc L-2,4-diaminobutyrate decarboxylase
ECA2245 2545856 2546087 YP_050340.1 - 4-oxalocrotonate tautomerase
ECA2246 2546334 2547111 YP_050341.1 - hypothetical protein
ECA2247 2547154 2548117 YP_050342.1 - Arac-family transcriptional regulator
ECA2248 2548322 2549147 YP_050343.1 - mannosyl-3-phosphoglycerate phosphatase
PectoI90
========
ECA2253 2553107 2553458 YP_050348.1 - hypothetical protein

ECA2254 2553610 2554228 YP_050349.1 - putative lipoprotein
ECA2255 2554333 2554633 YP_050350.1 - hypothetical protein
ECA2256 2554697 2556170 YP_050351.1 - putative transporter
PectoI91
========
ECA2264 2564897 2566448 YP_050359.1 - hypothetical protein
ECA2265 2566549 2567527 YP_050360.1 - hypothetical protein
PectoI92
========
ECA2306 2612726 2614289 YP_050401.1 - probable phage integrase
PectoI93
========
ECA2352 2665343 2666324 YP_050447.1 - putative 6-phosphofructokinase isozyme II
ECA2353 2666334 2666628 YP_050448.1 - hypothetical protein
ECA2354 2666641 2667979 YP_050449.1 ulaA ascorbate-specific PTS system enzyme IIC
ECA2355 2668043 2668322 YP_050450.1 - hypothetical protein
ECA2356 2668328 2668766 YP_050451.1 - putative sugar phosphotransferase enzyme IIa component
ECA2357 2668762 2669296 YP_050452.1 - putative kinase
ECA2358 2669332 2670232 YP_050453.1 - putative aldolase
PectoI94
========
ECA2381 2692520 2693453 YP_050476.1 - LysR-family transcriptional regulator
ECA2382 2693564 2694197 YP_050477.1 - putative NADP oxidoreductase coenzyme F420-dependent
protein
ECA2383 2694344 2695058 YP_050478.1 - putative isochorismatase
PectoI95
========
ECA2409 2721433 2722009 YP_050504.1 - hypothetical protein
ECA2410 2722185 2723580 YP_050505.1 - putative sodium:dicarboxylate symporter
PectoI96
========
ECA2430 2744003 2745227 YP_050525.1 - hypothetical protein
ECA2431 2745410 2746160 YP_050526.1 - two-component response regulator
ECA2432 2746156 2747899 YP_050527.1 - two-component sensor kinase
ECA2433 2748046 2750215 YP_050528.1 - putative signaling protein
ECA2434 2750607 2750931 YP_050529.1 - hypothetical protein
ECA2435 2751258 2751993 YP_050530.1 rdgA regulator of pectin lyase production
ECA2437 2752446 2752797 YP_050531.1 rdgB regulator of pectin lyase production
ECA2438 2753271 2754381 YP_050532.1 - putative electron transport protein
PectoI97
========
ECA2454 2769656 2770193 YP_050547.1 ogt probable methylated DNA--protein-cysteine methyltransferase
ECA2455 2770375 2771476 YP_050548.1 fsr fosmidomycin resistance protein
ECA2456 2771526 2771685 YP_050549.1 - probable transcriptional regulator (partial)
PectoI98
========
ECA2465 2782997 2783189 YP_050558.1 - hypothetical protein
ECA2466 2783207 2783903 YP_050559.1 - hypothetical protein
ECA2467 2783942 2784188 YP_050560.1 - hypothetical protein
PectoI99
========
ECA2497 2814295 2815513 YP_050589.1 - putative signal transduction protein
ECA2498 2815600 2816095 YP_050590.1 - hypothetical protein
PectoI100
=========
ECA2508 2825270 2826881 YP_050599.1 - putative integrase/recombinase
PectoI101
=========
ECA2513 2830277 2830577 YP_050604.1 - hypothetical protein
PectoI102
=========
ECA2598 2935460 2935850 YP_050689.1 - hypothetical protein
ECA2599 2935892 2936141 YP_050690.1 - bacteriophage regulatory protein
ECA2600 2936177 2937215 YP_050691.1 - bacteriophage late gene control protein D
ECA2601 2937467 2938652 YP_050692.1 - major tail sheath protein FI
ECA2602 2938652 2939168 YP_050693.1 - major tail tube protein FII
PectoI103
=========
ECA2636 2965318 2965618 YP_050728.1 - phage repressor protein C
ECA2637 2965684 2966671 YP_050729.1 - phage integrase
ECA2639 2967602 2969171 YP_050730.1 - putative hydantoinase
ECA2640 2969181 2970291 YP_050731.1 - hypothetical protein
ECA2641 2970343 2971648 YP_050732.1 - permease
ECA2642 2971921 2972863 YP_050733.1 - LysR-family transcriptional regulator
PectoI104
=========
ECA2654 2988413 2989868 YP_050745.1 - GntR-family transcriptional regulator
ECA2655 2989859 2990339 YP_050746.1 - hypothetical protein
PectoI105
=========
ECA2692 3027382 3028801 YP_050783.1 - putative flavin-containing amine oxidase
PectoI106
=========

ECA2757	3100257	3100503	YP_050848.1	-	hypothetical protein
ECA2758	3100492	3100777	YP_050849.1	-	hypothetical protein
ECA2759	3100822	3101182	YP_050850.1	-	hypothetical protein
ECA2760	3101845	3102175	YP_050851.1	-	hypothetical protein
ECA2761	3102234	3102771	YP_050852.1	-	hypothetical protein
PectoI107					
=========					
ECA2775	3115104	3115545	YP_050866.1	-	hypothetical protein
ECA2776	3115833	3116145	YP_050867.1	-	hypothetical protein
ECA2777	3116137	3116467	YP_050868.1	-	hypothetical protein
PectoI108					
=========					
ECA2794	3134964	3135321	YP_050885.1	-	hypothetical protein
ECA2795	3135381	3135924	YP_050886.1	cyaB	adenylate cyclase
PectoI109					
=========					
ECA2840	3182763	3184236	YP_050931.1	-	hypothetical protein
ECA2841	3184359	3185100	YP_050932.1	-	GntR-family transcriptional regulator
ECA2842	3185327	3186677	YP_050933.1	pcaB	3-carboxy-cis,cis-muconate cycloisomerase
ECA2843	3186823	3188245	YP_050934.1	-	putative sodium:sulfate symporter
ECA2844	3188335	3188503	YP_050935.1	-	hypothetical protein
ECA2845	3188703	3190713	YP_050936.1	nosA	TonB dependent receptor
ECA2846	3190821	3191298	YP_050937.1	-	hypothetical protein
ECA2847	3191687	3191906	YP_050938.1	-	hypothetical protein
PectoI110					
=========					
ECA2855	3197248	3197722	YP_050946.1	-	hypothetical protein
ECA2856	3197844	3198195	YP_050947.1	-	hypothetical protein
ECA2857	3198252	3199086	YP_050948.1	-	hypothetical protein
PectoI111					
=========					
ECA2870	3212037	3212454	YP_050961.1	-	hypothetical protein
ECA2871	3212529	3212853	YP_050962.1	-	hypothetical protein
ECA2872	3213660	3215115	YP_050963.1	-	putative flagellin
ECA2873	3215244	3215712	YP_050964.1	-	putative transcriptional regulator
PectoI112					
=========					
ECA2879	3226142	3227396	YP_050970.1	-	integrase
PectoI113					
=========					
ECA2884	3232085	3232373	YP_050975.1	-	hypothetical protein
ECA2885	3233242	3234655	YP_050976.1	cycA	D-alanine/D-serine/glycine permease
ECA2886	3234824	3235091	YP_050977.1	-	hypothetical protein
ECA2887	3235087	3235258	YP_050978.1	-	hypothetical protein
ECA2888	3235471	3235747	YP_050979.1	nac	nitrogen assimilation regulatory protein (partial)
ECA2889	3236380	3237667	YP_050980.1	intB	integrase
PectoI114					
=========					
ECA2925	3268213	3268972	YP_051016.1	-	probable short chain dehydrogenase
ECA2926	3269019	3269145	YP_051017.1	-	putative glutathione-S-transferase (partial)
ECA2927	3269196	3270420	YP_051018.1	-	probable transporter
PectoI115					
=========					
ECA2931	3276182	3276797	YP_051022.1	-	TetR-family transcriptional regulator
ECA2932	3276883	3278356	YP_051023.1	-	putative outer membrane efflux lipoprotein
PectoI116					
=========					
ECA2940	3286144	3286675	YP_051031.1	nifF	flavodoxin FldA
ECA2941	3286908	3287706	YP_051032.1	nifM	nitrogen fixation protein
PectoI117					
=========					
ECA2950	3295377	3295986	YP_051041.1	-	TetR-family regulatory protein
PectoI118					
=========					
ECA2983	3332534	3332705	YP_051074.1	-	hypothetical protein
ECA2984	3332712	3333801	YP_051075.1	xenA	flavoprotein xenobiotic reductase
ECA2985	3333852	3334194	YP_051076.1	-	ArsR-family transcriptional regulator
ECA2986	3334273	3334795	YP_051077.1	-	probable oxidase
ECA2987	3334879	3335344	YP_051078.1	-	hypothetical protein
ECA2988	3335433	3335667	YP_051079.1	-	conserved hypothetical protein (partial)
PectoI119					
=========					
ECA3075	3432148	3432922	YP_051165.1	-	putative fimbrial chaperone
ECA3076	3432965	3435347	YP_051166.1	-	putative fimbrial usher protein
ECA3077	3435351	3436362	YP_051167.1	-	hypothetical protein
ECA3078	3436354	3438550	YP_051168.1	fadJ	multifunctional fatty acid oxidation complex subunit alpha
ECA3079	3438546	3439860	YP_051169.1	fadI	3-ketoacyl-CoA thiolase
PectoI120					
=========					
ECA3088	3448806	3449568	YP_051178.1	-	hypothetical protein
ECA3089	3449696	3450047	YP_051179.1	-	hypothetical protein
ECA3090	3450487	3451027	YP_051180.1	-	hypothetical protein
PectoI121					

=========					
ECA3111	3471137	3473117	YP_051201.1	pehX	exo-poly-alpha-D-galacturonosidase
ECA3112	3473255	3474824	YP_051202.1	-	pectate lyase
PectoI122					
=========					
ECA3116	3478243	3479224	YP_051206.1	-	putative mandelate racemase/muconate lactonizing enzyme
ECA3117	3479266	3480127	YP_051207.1	dat	D-alanine aminotransferase
ECA3118	3480207	3482655	YP_051208.1	-	putative mechanosensitive ion channel protein
ECA3119	3483327	3484431	YP_051209.1	-	hypothetical protein
ECA3120	3484441	3486097	YP_051210.1	hsdS	subunit S of type I restriction-modification system
ECA3121	3486096	3487569	YP_051211.1	hsdM	subunit M of type I restriction-modification system
ECA3122	3487641	3490068	YP_051212.1	hsdR	type I restriction enzyme EcoEI R protein
PectoI123					
=========					
ECA3172	3542090	3543101	YP_051261.1	galR	DNA-binding transcriptional repressor
ECA3173	3543586	3544894	YP_051262.1	lamB	maltoporin
PectoI124					
=========					
ECA3180	3554168	3554510	YP_051269.1	-	PTS system, glucose-specific IIbc component (partial)
ECA3181	3554641	3556399	YP_051270.1	dld	D-lactate dehydrogenase
PectoI125					
=========					
ECA3202	3583504	3584407	YP_051291.1	-	LysR-family transcriptional regulator
ECA3203	3584422	3585196	YP_051292.1	-	hypothetical protein
ECA3204	3585400	3585751	YP_051293.1	-	hypothetical protein
ECA3205	3586090	3587221	YP_051294.1	-	putative exported choloylglycine hydrolase
ECA3206	3587319	3587535	YP_051295.1	-	transcriptional regulator
ECA3207	3587835	3588690	YP_051296.1	-	hypothetical protein
PectoI126					
=========					
ECA3213	3595534	3596425	YP_051302.1	-	AraC-family transcriptional regulator
ECA3214	3596585	3597341	YP_051303.1	-	probable short chain dehydrogenase
ECA3215	3597444	3598404	YP_051304.1	-	putative transport protein
PectoI127					
=========					
ECA3268	3672394	3674611	YP_051357.1	-	putative toxin secretion ATP-binding protein
ECA3269	3674597	3675959	YP_051358.1	-	HlyD family secretion protein
ECA3270	3676001	3677516	YP_051359.1	-	hypothetical protein
ECA3271	3678303	3679095	YP_051360.1	-	EAL domain containing protein involved in flagellar function
ECA3272	3679112	3679367	YP_051361.1	-	hypothetical protein
ECA3273	3679565	3679826	YP_051362.1	-	putative ferredoxin
PectoI128					
=========					
ECA3353	3770089	3770806	YP_051442.1	-	putative two-component response-regulatory protein YehT
ECA3354	3770827	3771202	YP_051443.1	-	hypothetical protein
PectoI129					
=========					
ECA3370	3786824	3787319	YP_051459.1	-	competence damage-inducible protein A
ECA3371	3787554	3787890	YP_051460.1	-	hypothetical protein
ECA3372	3788370	3788859	YP_051461.1	-	hypothetical protein
ECA3373	3788941	3789616	YP_051462.1	marC	multiple drug resistance protein MarC
ECA3374	3789807	3791406	YP_051463.1	-	putative signaling membrane protein
ECA3375	3791665	3792868	YP_051464.1	-	probable transporter
ECA3376	3792983	3793865	YP_051465.1	-	LysR-family transcriptional regulator
ECA3377	3794081	3794717	YP_051466.1	-	glutaredoxin 2
ECA3378	3794815	3795238	YP_051467.1	-	putative bacteriocin immunity protein
ECA3379	3795369	3796710	YP_051468.1	-	putative glycosyltransferase
ECA3380	3796721	3797654	YP_051469.1	pvcB	pyoverdine biosynthesis protein
ECA3381	3797643	3798624	YP_051470.1	pvcA	pyoverdine biosynthesis protein
ECA3381A	3798620	3798818	YP_051471.1	-	putative bacteriocin immunity protein
ECA3382	3799228	3799516	YP_051472.1	-	hypothetical protein
ECA3382A	3799503	3799749	YP_051473.1	-	hypothetical protein
ECA3383	3799872	3800157	YP_051474.1	-	hypothetical protein
ECA3384	3800169	3800646	YP_051475.1	-	hypothetical protein
ECA3385	3800656	3800947	YP_051476.1	-	hypothetical protein
ECA3386	3801261	3801546	YP_051477.1	-	hypothetical protein
ECA3387	3801550	3801913	YP_051478.1	-	hypothetical protein
PectoI130					
=========					
ECA3420	3828783	3829221	YP_051511.1	-	hypothetical protein
ECA3421	3829217	3834074	YP_051512.1	-	Rhs protein
ECA3422	3834094	3835012	YP_051513.1	-	hypothetical protein
ECA3423	3835008	3835965	YP_051514.1	-	hypothetical protein
PectoI131					
=========					
ECA3451	3872982	3873474	YP_051541.1	-	hypothetical protein
ECA3452	3873466	3874063	YP_051542.1	fic	cell filamentation protein
ECA3453	3874070	3874256	YP_051543.1	-	putative phage capsid protein (partial)
ECA3454	3874252	3874501	YP_051544.1	-	bacteriophage regulatory protein
ECA3455	3874619	3874775	YP_051545.1	-	hypothetical protein
PectoI132					
=========					
ECA3461	3880771	3881191	YP_051551.1	-	hypothetical protein

Pectol133
=========

ECA3480	3901552	3902074	YP_051570.1	-	hypothetical protein
ECA3481	3902192	3902711	YP_051571.1	-	hypothetical protein

Pectol134
=========

ECA3548	3976722	3978447	YP_051636.1	-	hypothetical protein
ECA3549	3978973	3979714	YP_051637.1	-	putative signaling protein

Pectol135
=========

ECA3556	3987236	3988169	YP_051644.1	-	LysR-family transcriptional regulator
ECA3557	3988267	3989257	YP_051645.1	-	metallo hydrolase

Pectol136
=========

ECA3625	4074060	4075893	YP_051713.1	-	hypothetical protein
ECA3626	4075861	4076878	YP_051714.1	-	putative type II/IV secretion system protein

Pectol137
=========

ECA3634	4081911	4082829	YP_051722.1	-	LysR-family transcriptional regulator
ECA3635	4082923	4084141	YP_051723.1	-	hypothetical protein

Pectol138
=========

ECA3657	4106202	4106496	YP_051745.1	-	hypothetical protein
ECA3658	4106531	4107239	YP_051746.1	-	hypothetical protein
ECA3659	4107616	4109308	YP_051747.1	-	hypothetical protein

Pectol139
=========

ECA3666	4116962	4117631	YP_051754.1	-	hypothetical protein
ECA3667	4117794	4118124	YP_051755.1	-	putative lipoprotein

Pectol140
=========

ECA3671	4121032	4121530	YP_051759.1	-	hypothetical protein
ECA3672	4121650	4122130	YP_051760.1	-	hypothetical protein
ECA3673	4122178	4122517	YP_051761.1	-	hypothetical protein
ECA3674	4122636	4123044	YP_051762.1	-	hypothetical protein
ECA3675	4123036	4123897	YP_051763.1	-	hypothetical protein

Pectol141
=========

ECA3713	4156500	4156845	YP_051801.1	-	putative phage-related membrane protein

Pectol142
=========

ECA3741	4178590	4180153	YP_051830.1	-	putative bacteriophage tail fiber protein
ECA3742	4180152	4180770	YP_051831.1	-	putative bacteriophage tail fiber assembly protein
ECA3743	4180821	4181157	YP_051832.1	-	hypothetical protein

Pectol143
=========

ECA3768	4209248	4211753	YP_051857.1	-	putative formate acetyltransferase
ECA3769	4211838	4212789	YP_051858.1	-	putative pyruvate formate-lyase activating enzyme
ECA3770	4213114	4213531	YP_051859.1	qacH	quaternary ammonium compound resistance protein
ECA3771	4213548	4213875	YP_051860.1	-	putative multidrug resistance protein
ECA3772	4214034	4215231	YP_051861.1	-	LacI-family transcriptional regulator
ECA3773	4215530	4215974	YP_051862.1	sgcA	putative phosphotransferase enzyme II, A component
ECA3774	4215996	4216284	YP_051863.1	-	hypothetical protein

Pectol144
=========

ECA3780	4222571	4222883	YP_051869.1	-	ArsR-family transcriptional regulator
ECA3781	4223013	4225257	YP_051870.1	fepA	TonB dependent siderophore receptor

Pectol145
=========

ECA3792	4239709	4241143	YP_051881.1	-	hypothetical protein
ECA3793	4241117	4242077	YP_051882.1	-	putatuve glycosysl hydrolase

Pectol146
=========

ECA3835	4286954	4287266	YP_051923.1	-	hypothetical protein
ECA3836	4287262	4287997	YP_051924.1	-	hypothetical protein
ECA3837	4288181	4289018	YP_051925.1	-	AraC-family transcriptional regulator
ECA3838	4289236	4290856	YP_051926.1	-	methyl-accepting chemotaxis protein
ECA3839	4290970	4291162	YP_051927.1	-	putative matrix protein
ECA3840	4291682	4292240	YP_051928.1	-	hypothetical protein
ECA3841	4292627	4294286	YP_051929.1	-	putative extracellular solute-binding protein
ECA3842	4294344	4295274	YP_051930.1	-	putative isoflavone oxidoreductase

Pectol147
=========

ECA3943	4413808	4415560	YP_052030.1	-	hypothetical protein
ECA3944	4415556	4415766	YP_052031.1	-	putative phage-related protein
ECA3945	4415813	4416323	YP_052032.1	-	hypothetical protein
ECA3946	4417002	4418481	YP_052033.1	-	hypothetical protein
ECA3947	4418703	4419630	YP_052034.1	-	putative D-isomer specific 2-hydroxyacid dehydrogenase
ECA3948	4419691	4420816	YP_052035.1	-	hypothetical protein
ECA3949	4420812	4421475	YP_052036.1	-	hypothetical protein
ECA3950	4421778	4422828	YP_052037.1	-	putative zinc-binding dehydrogenase
ECA3951	4423295	4423913	YP_052038.1	-	LysE-type translocator
ECA3952	4424159	4425050	YP_052039.1	-	LysR-family transcriptional regulator

ECA3953	4425069	4426236	YP_052040.1	-	probable transporter
ECA3954	4426300	4427116	YP_052041.1	-	ABC transporter, ATP-binding protein
ECA3955	4427115	4427880	YP_052042.1	-	ABC transporter protein, membrane protein
ECA3956	4427895	4428915	YP_052043.1	-	ABC transporter, substrate binding protein
PectoI148					
=========					
ECA3960	4431769	4432609	YP_052047.1	-	putative hydrolase
ECA3961	4432619	4433255	YP_052048.1	-	hypothetical protein
ECA3962	4433306	4433852	YP_052049.1	-	hypothetical protein
ECA3963	4433914	4434142	YP_052050.1	-	hypothetical protein
PectoI149					
=========					
ECA4045	4511593	4512688	YP_052132.1	pmrB	sensor protein BasS/PmrB
ECA4046	4512739	4514011	YP_052133.1	-	putative serine hydroxymethyltransferase
ECA4047	4514043	4514856	YP_052134.1	-	putative alanine racemase
ECA4048	4514959	4516456	YP_052135.1	-	GntR-family transcriptional regulator
PectoI150					
=========					
ECA4052	4518537	4518864	YP_052139.1	-	hypothetical protein
ECA4053	4518863	4519643	YP_052140.1	-	short chain dehydrogenase
PectoI151					
=========					
ECA4078	4546706	4546904	YP_052165.1	-	hypothetical protein
ECA4079	4546958	4549529	YP_052166.1	nirB	nitrite reductase [NAD(P)H] large subunit
ECA4080	4549525	4549894	YP_052167.1	nirD	nitrite reductase small subunit
ECA4081	4549961	4551341	YP_052168.1	cysG	siroheme synthase
ECA4082	4551833	4552562	YP_052169.1	occQ	octopine transport system permease protein
ECA4083	4552561	4553296	YP_052170.1	occM	octopine transport system permease protein
ECA4084	4553292	4554057	YP_052171.1	occP	octopine permease ATP-binding protein P
PectoI152					
=========					
ECA4095	4564709	4565087	YP_052182.1	-	hypothetical protein
ECA4096	4565083	4565671	YP_052183.1	-	hypothetical protein
ECA4097	4565663	4566233	YP_052184.1	-	hypothetical protein
PectoI153					
=========					
ECA4109	4579773	4580184	YP_052196.1	-	hypothetical protein
ECA4110	4580246	4581398	YP_052197.1	-	putative periplasmic substrate-binding protein
ECA4111	4581390	4582641	YP_052198.1	-	probable transporter
ECA4112	4582714	4583872	YP_052199.1	-	putative octopine/opine/tauropine dehydrogenase
ECA4113	4583882	4584899	YP_052200.1	-	putative cystathionine beta-synthase
ECA4114	4584912	4586316	YP_052201.1	-	putative lysine/ornithine decarboxylase
ECA4115	4586357	4588553	YP_052202.1	-	TonB-dependent siderophore receptor
ECA4116	4588635	4590537	YP_052203.1	-	putative siderophore biosynthesis protein
ECA4117	4590787	4591669	YP_052204.1	-	putative citrate lyase beta chain
ECA4118	4591737	4592217	YP_052205.1	greB	transcription elongation factor GreB
ECA4119	4592391	4594107	YP_052206.1	-	putative acyltransferase
PectoI154					
=========					
ECA4129	4607030	4607510	YP_052216.1	-	hypothetical protein
ECA4130	4607522	4609268	YP_052217.1	-	alkaline phosphatase
PectoI155					
=========					
ECA4219	4720643	4721531	YP_052306.1	-	LysR-family transcriptional regulator
ECA4220	4721632	4722259	YP_052307.1	-	hypothetical protein
PectoI156					
=========					
ECA4280	4797652	4797877	YP_052367.1	-	hypothetical protein
ECA4281	4797947	4798346	YP_052368.1	-	putative phage regulatory protein
PectoI157					
=========					
ECA4289	4804284	4804509	YP_052376.1	-	hypothetical protein
ECA4290	4804579	4804978	YP_052377.1	-	putative phage regulatory protein
ECA4291	4805079	4807998	YP_052378.1	-	DnaG primase-like protein
ECA4292	4808057	4809110	YP_052379.1	xerC	site-specific tyrosine recombinase XerC
ECA4293	4810141	4810594	YP_052380.1	-	hypothetical protein
ECA4294	4810593	4811115	YP_052381.1	-	hypothetical protein
PectoI158					
=========					
ECA4323	4841928	4842345	YP_052410.1	-	hypothetical protein
ECA4324	4842346	4843042	YP_052411.1	-	hypothetical protein
ECA4325	4843158	4843920	YP_052412.1	-	DeoR-family trancriptional regulator
ECA4326	4843916	4844132	YP_052413.1	-	hypothetical protein
PectoI159					
=========					
ECA4355	4876118	4876685	YP_052442.1	dcrB	hypothetical protein
ECA4356	4877183	4879178	YP_052443.1	-	hypothetical protein
ECA4357	4879177	4879885	YP_052444.1	-	putative lipoprotein
PectoI160					
=========					
ECA4363	4886992	4888174	YP_052450.1	-	hypothetical protein
ECA4364	4888642	4890289	YP_052451.1	-	hypothetical protein
ECA4365	4890290	4890512	YP_052452.1	-	hypothetical protein

ECA4366	4890508	4891822	YP_052453.1	-	hypothetical protein
PectoI161					
=========					
ECA4380	4911486	4912836	YP_052465.1	-	probable sugar transporter
PectoI162					
=========					
ECA4383	4915140	4916586	YP_052468.1	uxaB2	altronate oxidoreductase
PectoI163					
=========					
ECA4396	4934119	4934395	YP_052481.1	-	hypothetical protein
ECA4397	4934378	4935272	YP_052482.1	-	hypothetical protein
PectoI164					
=========					
ECA4426	4965492	4967043	YP_052511.1	-	putative extracellular solute-binding protein
ECA4427	4967185	4968082	YP_052512.1	-	LysR-family transcriptional regulator
ECA4428	4968227	4969043	YP_052513.1	-	predicted hydrolase
ECA4429	4969442	4969667	YP_052514.1	-	putative transposase
PectoI165					
=========					
ECA4432	4972973	4974416	YP_052517.1	bglA	6-phospho-beta-glucosidase
ECA4433	4974428	4974776	YP_052518.1	-	pts system, cellobiose-specific IIa component
ECA4434	4974765	4976079	YP_052519.1	-	PTS system, IIbc component
ECA4435	4976095	4976401	YP_052520.1	-	pts system, IIb component
ECA4436	4976614	4977652	YP_052521.1	-	LacI-family transcriptional regulator
PectoI166					
=========					
ECA4447	4989708	4990275	YP_052532.1	-	hypothetical protein
ECA4448	4990459	4991356	YP_052533.1	-	hypothetical protein
ECA4449	4991420	4992272	YP_052534.1	-	hypothetical protein
ECA4450	4992372	4992792	YP_052535.1	-	hypothetical protein
ECA4451	4992908	4993331	YP_052536.1	hmsS	putative hemin storage protein
PectoI167					
=========					
ECA4457	5001993	5003496	YP_052541.1	smvA	putative methyl viologen resistance protein
PectoI168					
=========					
ECA4505	5044690	5045479	YP_052588.1	-	putative acyltransferase
ECA4506	5045471	5046200	YP_052589.1	-	hypothetical protein
