# Supplementary material for: Microarray Comparative Genomic Hybridisation Analysis Incorporating Genomic Organisation, and Application to Enterobacterial Plant Pathogens
Source: PLoS Comput Biol. 2009 Aug 21;5(8):e1000473. doi: 10.1371/journal.pcbi.1000473 (PMC2718846; doi:10.1371/journal.pcbi.1000473)
Supplement: Table S6 — Islands of Pba1043 CDS predicted by aCGH to have be Pba-specific (PbaI), or to have no orthologues in either Pcc193 (PccI). (0.05 MB PDF) [file pcbi.1000473.s006.pdf]

Supplementary Table 6: Islands of *Pba*1043 CDS predicted by aCGH to have be *Pba*-specific (PbaI), or to have no orthologues in either *Pcc*193 (PccI) or *Dda*3937 (DdaI). These islands additionally contain a statistical abundance of CDS that have putative orthologues either only in plant-associated bacteria (PAB), or to share greater sequence identity with their orthologue(s) in PAB than with their orthologues in animal-associated enterobacteria (AAE).

Locus tags	Pba-specific	Absent in Pcc193	Absent in Dda3937	Putative phenotype
ECA0600-ECA0610	PbaI7	PccI7	-	*cfa* biosynthesis
ECA0849-ECA0852	-	-	DdaI36	sugar ABC transport
ECA1477-ECA1481	PbaI14	PccI15	DdaI57	Hypothetical
ECA1584-ECA1598	PbaI16	PccI17	-	Hypothetical
ECA2208-ECA2216	-	-	DdaI85	oxidoreductase, *ogt*
ECA2232-ECA2234	PbaI26	-	-	Hypothetical
ECA2381-ECA2383	-	-	DdaI93	putative isochorismatase
ECA2933-ECA2936	PbaI39	PccI42	DdaI110	*nifQ*
ECA2951	PbaI40	-	-	Transporter
ECA2951-ECA2957	-	PccI43	-	Nitrogen fixation
ECA3202-ECA3207	-	-	DdaI120	Hypothetical
ECA3556-ECA3557	-	-	DdaI131	Metal hydrolase
ECA4052-ECA4053	-	-	DdaI148	Hypothetical
ECA4219-ECA4220	-	-	DdaI154	Hypothetical
ECA4481-ECA4493	-	PccI59	-	allphanate hydrolase
