# Supplementary material for: Microarray Comparative Genomic Hybridisation Analysis Incorporating Genomic Organisation, and Application to Enterobacterial Plant Pathogens
Source: PLoS Comput Biol. 2009 Aug 21;5(8):e1000473. doi: 10.1371/journal.pcbi.1000473 (PMC2718846; doi:10.1371/journal.pcbi.1000473)
Supplement: Figure S1 — Scatter plot of percentage sequence identity for coding sequences in Pba1043 to Dda3937 by reciprocal best FASTA and BLASTN analyses. (0.31 MB PDF) [file pcbi.1000473.s007.pdf]

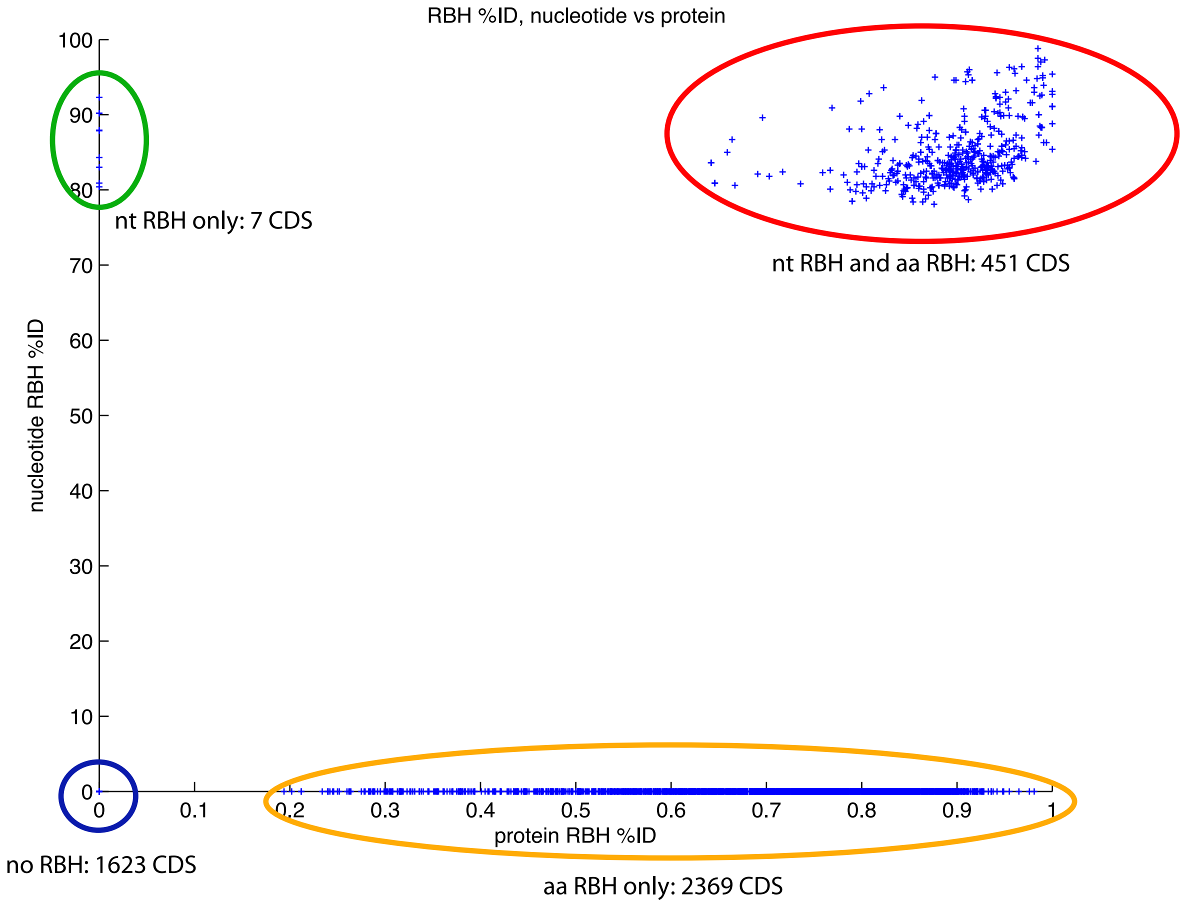

Supplementary Figure 1: Scatter plot of percentage sequence identity for reciprocal best hits (RBH) for coding sequences (CDS) in *Pba*1043 to *Dda*3937 by reciprocal best FASTA analysis (protein, x-axis) and reciprocal best BLASTN analysis (nucleotide, y-axis). CDS that do not have a RBH with either method are assigned a sequence identity of zero. Four classes of *Pba*1043 CDS can be distinguished: 451/4450 make RBH at both protein and nucleotide levels; 7/4450 make RBH only at the nucleotide level; 2369/4450 make RBH only at the protein level; and 1623/4450 make no reciprocal best hits. The majority of *Pba*1043 CDS have an RBH with *Dda*3937, but approximately one third of the genome does not. There are very few sequences that are similar at the nucleotide, but not the protein level (suggestive of positive selection), but many that are similar at the protein, and not the nucleotide level (suggestive of neutral drift). The ‘core’ conserved set of CDS comprises only approximately 10% of the *Pba*1043 genome.
