# Supplementary material for: Microarray Comparative Genomic Hybridisation Analysis Incorporating Genomic Organisation, and Application to Enterobacterial Plant Pathogens
Source: PLoS Comput Biol. 2009 Aug 21;5(8):e1000473. doi: 10.1371/journal.pcbi.1000473 (PMC2718846; doi:10.1371/journal.pcbi.1000473)
Supplement: Figure S2 — Scatterplots of putative orthologue (RBH) nucleotide sequence identity. (1.54 MB PDF) [file pcbi.1000473.s008.pdf]

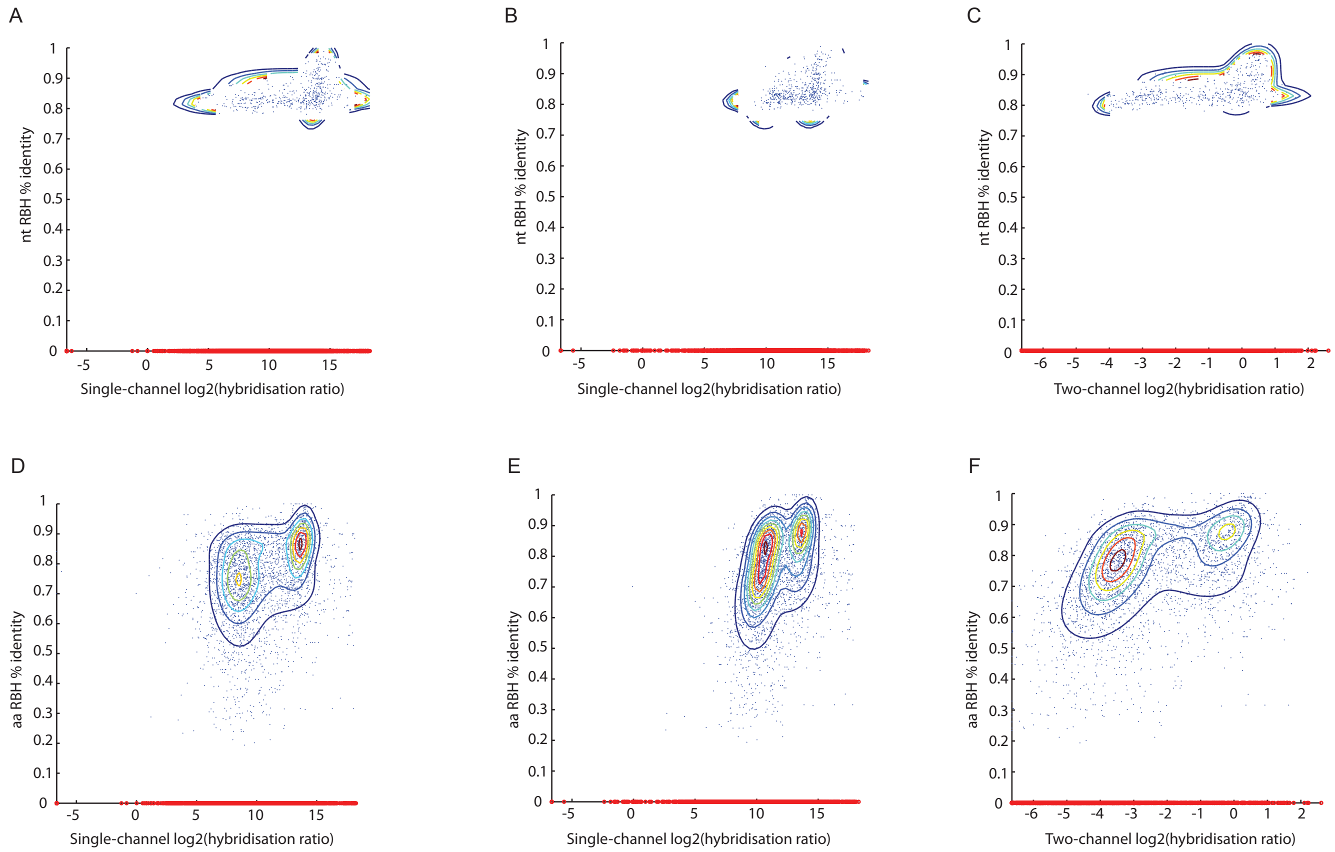

Supplementary Figure 2: Scatterplots of putative orthologue (RBH) nucleotide sequence identity against log-transformed hybridisation scores for *Dda*3937 gDNA with A) no reference gDNA; B) *Pba*1043 reference gDNA; and C) log-transformed hybridization ratio with *Pba*1043 reference gDNA, and of protein sequence identity against log-transformed hybridisation scores for *Dda*3937 gDNA with D) no reference gDNA; E) *Pba*1043 reference gDNA; and F) log-transformed hybridisation ratio with *Pba*1043 reference gDNA. Probes to *Pba*1043 sequences with no *Dda*3937 orthologue are indicated in red. In all cases, it is not possible to distinguish between 'present' and 'divergent' orthologues on the basis of hybridisation score or ratio, and probes to sequences with no orthologue span a range of hybridisation scores from extremely weak to very strong.
