# Supplementary material for: Microarray Comparative Genomic Hybridisation Analysis Incorporating Genomic Organisation, and Application to Enterobacterial Plant Pathogens
Source: PLoS Comput Biol. 2009 Aug 21;5(8):e1000473. doi: 10.1371/journal.pcbi.1000473 (PMC2718846; doi:10.1371/journal.pcbi.1000473)
Supplement: Figure S3 — Plot of predictions of the theshold and HMM methods as the threshold is varied in the aCGH experiment with Dda3937 against Pba1043 taking log transformed hybridisation ratios. (0.23 MB PDF) [file pcbi.1000473.s009.pdf]

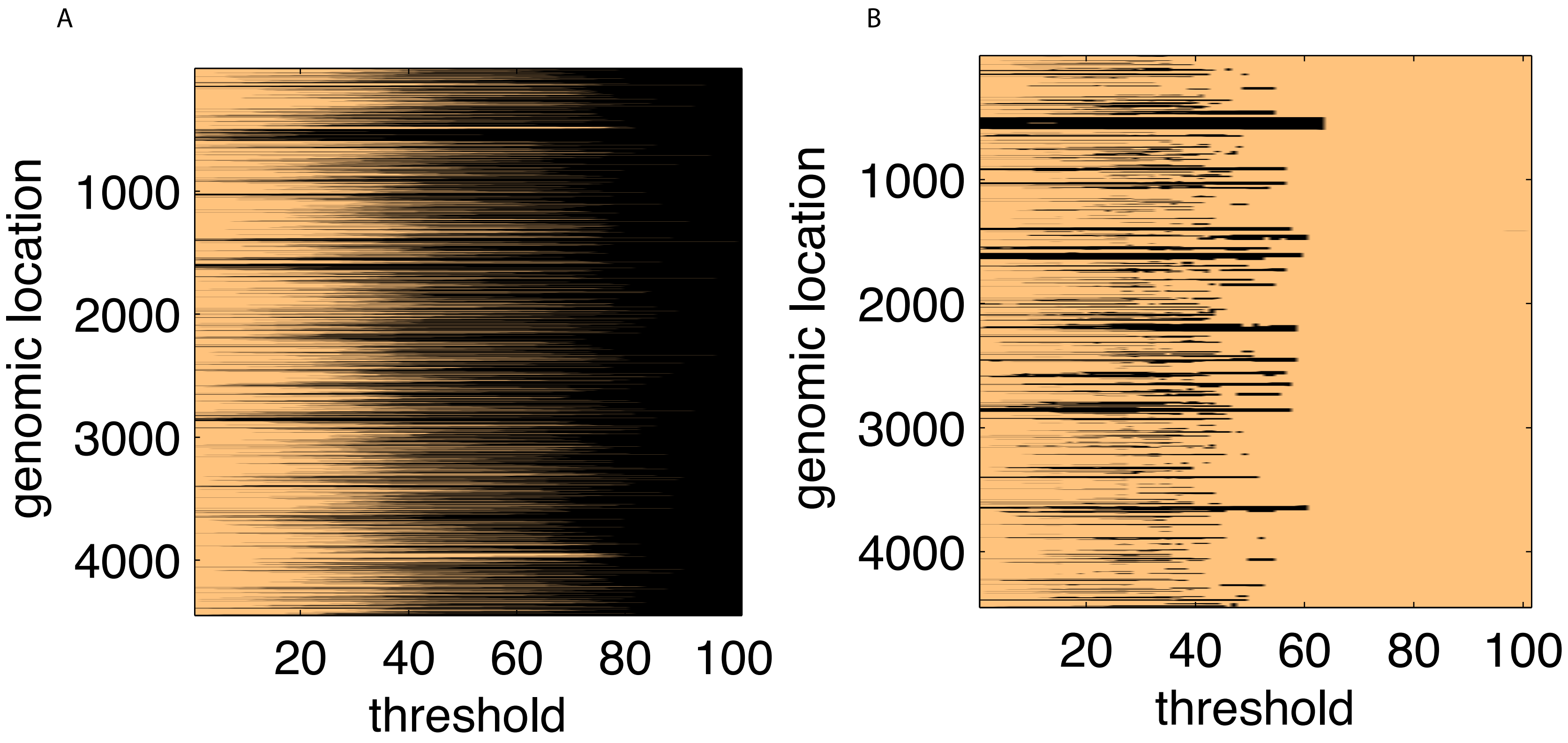

Supplementary Figure 3: Plot of the predictions of the threshold (A) and HMM (B) methods as the threshold is varied from the lowest to highest threshold value in the aCGH experiment with *Dda*3937 against a *Pba*1043 gDNA reference, taking log-transformed hybridisation ratios. Both methods predict a mixture of CDS with and without orthologues at the lowest threshold value. As the threshold increases from a minimum to a maximum, the proportion of CDS predicted to have no orthologue increases to 100% for the threshold method. At large threshold values, the HMM uniformly predicts the dominant state of the training data, which in this case is that all *Pba*1043 CDS have an orthologue in *Dda*3937.
