# Supplementary material for: Microarray Comparative Genomic Hybridisation Analysis Incorporating Genomic Organisation, and Application to Enterobacterial Plant Pathogens
Source: PLoS Comput Biol. 2009 Aug 21;5(8):e1000473. doi: 10.1371/journal.pcbi.1000473 (PMC2718846; doi:10.1371/journal.pcbi.1000473)
Supplement: Figure S4 — Plots of CDS from the reference organisms predicted to have or not to have a putative orthologue in the comparator organisms. (0.10 MB PDF) [file pcbi.1000473.s010.pdf]

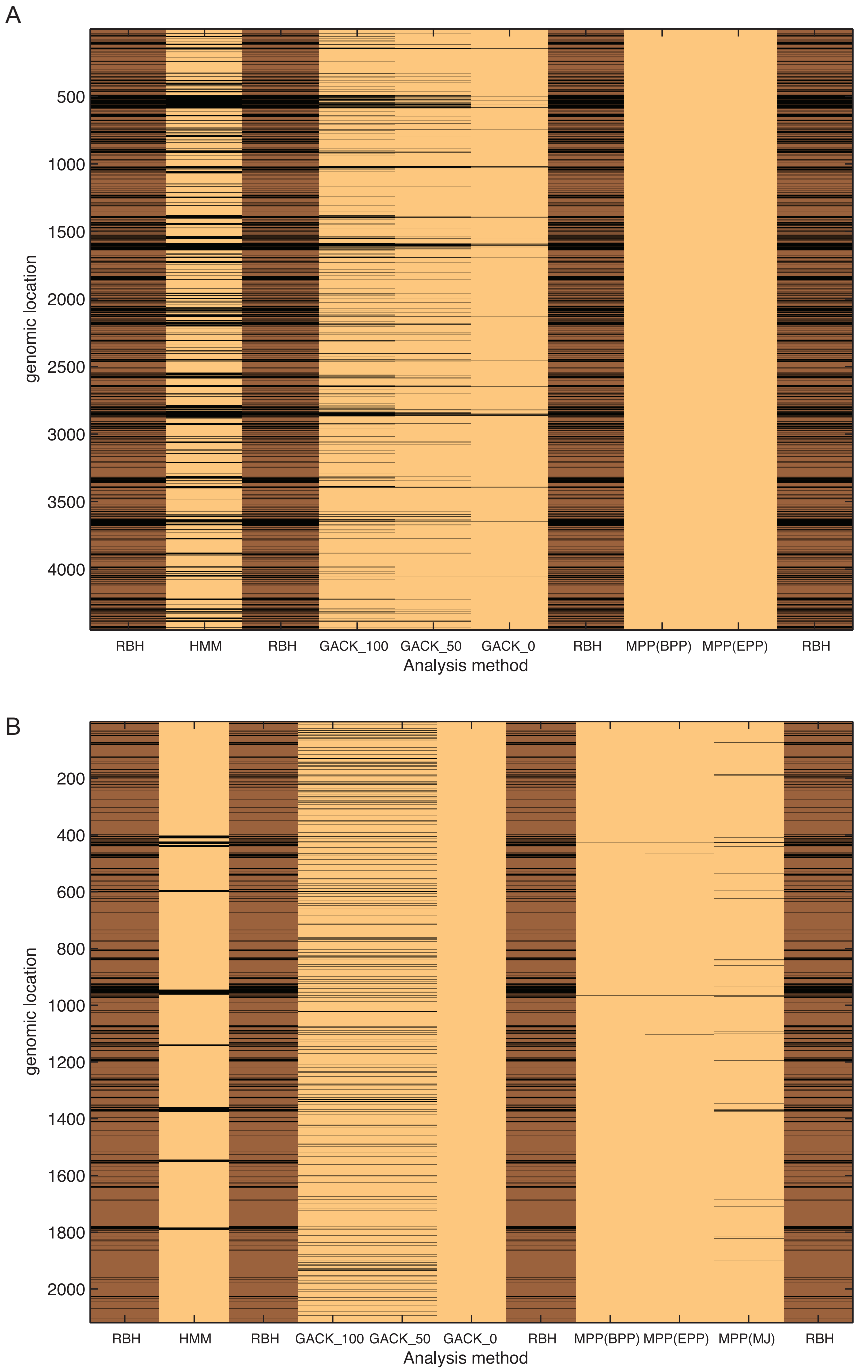

Supplementary Figure 4: Plots of the CDS from the reference organism predicted to have (copper) or not to have (black) a putative orthologue in the comparator organism for A) *Pba*1043:*Dda*3937 and B) *Lactococcus lactis* IL1403:MG1363 a CGH comparisons (the MPP MJ method failed to fit this data). In silico predictions derived from reciprocal best hit comparisons are labelled 'RBH' and indicated in dark copper, for comparison. Analytical methods are 'HMM': hidden Markov model described in the manuscript; GACK_100, GACK_50, GACK_0: GACK binary predictions with the EPP parameter set to 100%, 50% and 0%, respectively; MPP(BPP), MPP(EPP), MPP(MJ): MPP's BPP, EPP and MJ gene entry vector results, respectively. Qualitatively, the HMM method predicts larger contiguous islands with no putative orthologue than the other two methods, and also appears to be more successful at identifying the major genomic islands in the reference organism.
