# Supplementary material for: Microarray Comparative Genomic Hybridisation Analysis Incorporating Genomic Organisation, and Application to Enterobacterial Plant Pathogens
Source: PLoS Comput Biol. 2009 Aug 21;5(8):e1000473. doi: 10.1371/journal.pcbi.1000473 (PMC2718846; doi:10.1371/journal.pcbi.1000473)
Supplement: Figure S5 — Plot of the Pba1043 CDS with and without a predicted orthologue in Pba1039 and Pcc193. (0.09 MB PDF) [file pcbi.1000473.s011.pdf]

A

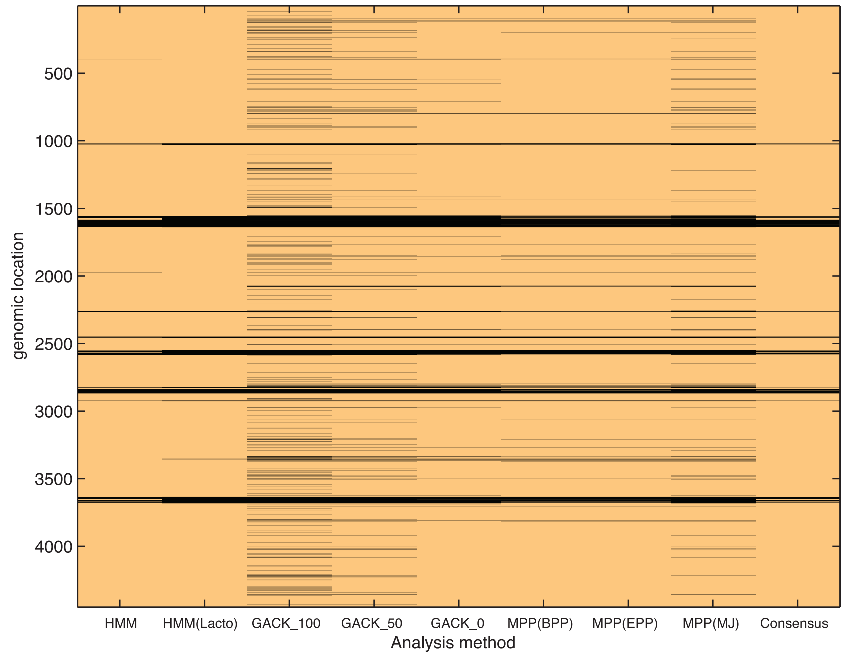

B

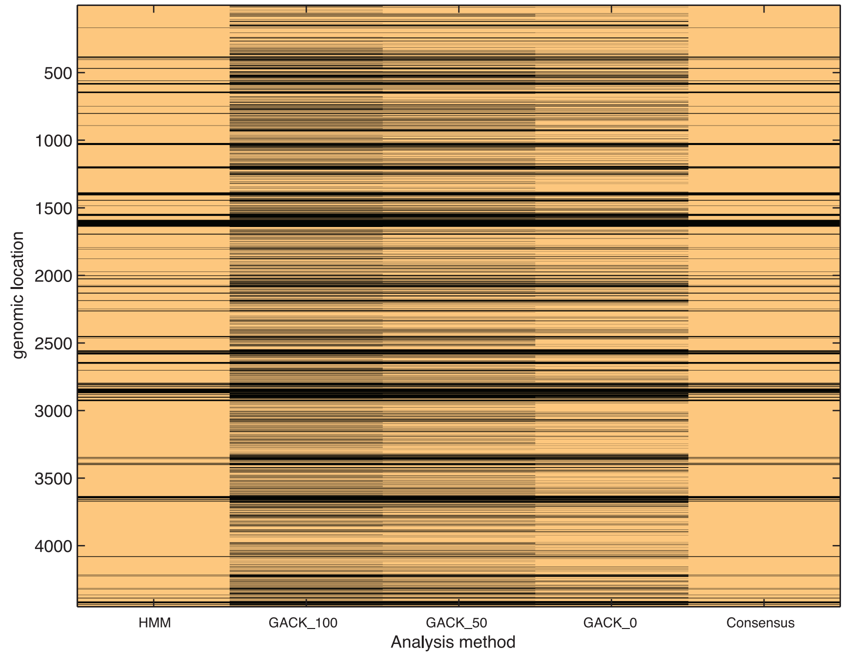

Supplementary Figure 5: Plot of the *Pba*1043 CDS with (copper) and without (black) a predicted orthologue in A) *Pba*1039 and B) *Pcc*193. The indicated analytical methods are HMM and HMM(lacto): hidden Markov models as described in the manuscript, trained on the *Pba*1043:*Dda*3937 and *L. lactis* comparisons, respectively; GACK_100, GACK_50, GACK_0: GACK with the EPP parameter set to 100%, 50% and 0%, respectively; MPP(BPP), MPP(EPP), MPP(MJ): MPP's BPP, EPP and MJ gene entry vector results, respectively. A consensus of *Pba*1043 CDS predicted to have no orthologue in each comparator organism is also indicated. All methods agree on the approximate location and size of the major genomic islands, but differ in the number and size of smaller islands. In particular, GACK appears to overpredict the number of *Pba*1043 CDS with no orthologue in *Pcc*193. For the *Pba*1043:*Pba*1039 comparison, the HMM derived from the Lactococcus comparison data appears to unite some neighbouring islands that are predicted by the HMM derived from the *Pba*1043:*Dda*3937 comparison; otherwise, their predictions are very similar.
